# Small molecule piperazinyl-benzimidazole antagonists of the gonadotropin-releasing hormone (GnRH) receptor†
†Electronic supplementary information (ESI) available: Detailed experimental procedures and analytical characterisation of all compounds. See DOI: 10.1039/c7md00320j


**DOI:** 10.1039/c7md00320j

**Published:** 2017-09-14

**Authors:** Richard Fjellaksel, Marc Boomgaren, Rune Sundset, Ira H. Haraldsen, Jørn H. Hansen, Patrick J. Riss

**Affiliations:** a Medical Imaging Group , Department of Clinical Medicine , UiT The Arctic University of Norway , 9037 Tromsø , Norway . Email: richard.fjellaksel@uit.no; b Drug Transport and Delivery Group , Department of Pharmacy , UiT The Arctic University of Norway , 9037 Tromsø , Norway; c Organic Chemistry Group , Department of Chemistry , UiT The Arctic University of Norway , 9037 Tromsø , Norway; d PET imaging center, division of diagnostics , UNN – University Hospital of North-Norway , 9038 Tromsø , Norway; e Department of neuropsychiatry and psychosomatic medicine , Oslo University Hospital , Oslo , Norway; f Realomics SFI, Department of Chemistry , University of Oslo , PO BOX 1033 , Oslo 0371 , Norway; g Norsk Medisinsk Syklotronsenter AS , Postboks 4950 Nydalen , 0424 Oslo , Norway

## Abstract

In this communication, we report the synthesis and characterization of a library of small molecule antagonists of the human gonadotropin releasing hormone receptor based upon the 2-(4-*tert*-butylphenyl)-4-piperazinyl-benzimidazole scaffold *via* Cu-catalysed azide alkyne cycloaddition. Our main purpose was to find a more soluble compound based on the WAY207024 lead with nanomolar potency to inhibit the GnRH receptor. A late stage diversification by the use of click chemistry was, furthermore developed to allow for expansion of the library in future optimisations. All compounds were tested in a functional assay to determine the individual potency of inhibiting stimulation of the receptor by the endogenous agonist GnRH. In conclusion, we found that compound **8a** showed improved solubility compared to WAY207024 and nanomolar affinity to GnRH receptor.

## Abstract 2

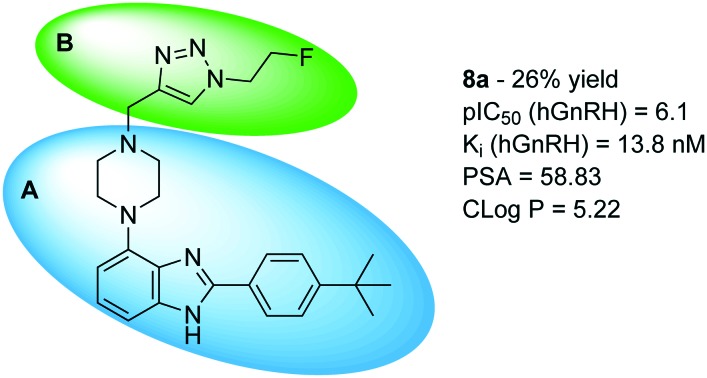
In this communication, we report the synthesis and characterization of a library of small molecule antagonists of the human gonadotropin releasing hormone receptor based upon the 2-(4-*tert*-butylphenyl)-4-piperazinyl-benzimidazole scaffold *via* Cu-catalysed azide alkyne cycloaddition.

## 

Gonadotropin-releasing hormone (GnRH) is a peptide hormone secreted from the hypothalamus into the hypophysial portal bloodstream. Once in circulation, the peptide acts as an endocrine signalling hormone mediating release of follicle stimulating hormone (FSH) and luteinizing hormone (LH) *via* gonadotropin-releasing hormone receptor (GnRHR) activation in the pituitary gland. LH and FSH directly regulate gender-specific production of androgens, estrogens, progesterone and inhibin in the gonads. The regulatory circuit is closed by the permeability of the blood–brain barrier to steroid hormones such as estrogen and testosterone, which form negative feedback loops. GnRH, LH and FSH are unlikely to permeate the blood–brain barrier, thus impeding any regulatory feedback on hormone balance, which substantiates the impact of testosterone and estrogen on neurochemical correlates of their respective effects on social behaviour, decision-making and ageing. The hypothalamic–pituitary–gonadal (HPG) signalling circuit is key to gender hormone homeostasis, which can lead to substantial implications in cognitive and behavioural traits.^1^–^4^

GnRH agonists are well-established pharmaceuticals used at low dose to stimulate hypofunctional GnRH in hypogonadism. At high dose, GnRH agonists deplete GnRH receptor function in the pituitary, limiting LH and FSH secretion and thereby the levels of androgens and estrogens in circulation and suppressing hormone production completely. GnRH receptor agonists play an important role in clinical care, *e.g.* in the treatment of hormone responsive cancer, reproductive diseases and for behavioral modification of sexual offenders *via* these mechanisms. However, GnRH agonists cannot address mild GnRH overfunction, which would require attenuation of the signaling circuit, rather than stimulation or depletion. To address this shortcoming, GnRH antagonists have attracted considerable attention in recent years specifically to treat diseases, which require some reduction of GnRH stimulation.^5^–^8^

We were interested in the development of a GnRH antagonist of moderate potency to address cognitive and behavioral correlates of GnRH-R attenuation. In our reasoning, a reversibly binding antagonist would reduce gonadal overstimulation by limiting the availability of binding sites by competition with the agonist. Thereby, the central effects of testosterone and estrogen would be buffered, while hormone homeostasis would be preserved. Small molecule antagonists of GnRHR are particularly interesting due to their direct, dose dependent inhibition of GnRH activity, lack of stimulatory side effects on the receptor and superior passive permeability into tissues relative to peptide agonists.

Several classes of small molecule antagonists have been developed previously. Pelletier and co-workers reported a 2-(4-*tert*-butylphenyl)-4-piperazinyl-benzimidazole-scaffold as a small molecule antagonist of the GnRHR with nanomolar inhibition potency, albeit low solubility was cited as a shortcoming by the authors.^9^,^10^ Further optimization lead to the discovery of the slightly more soluble WAY 207024 compound as shown in Fig. 1.^11^

**Fig. 1:**
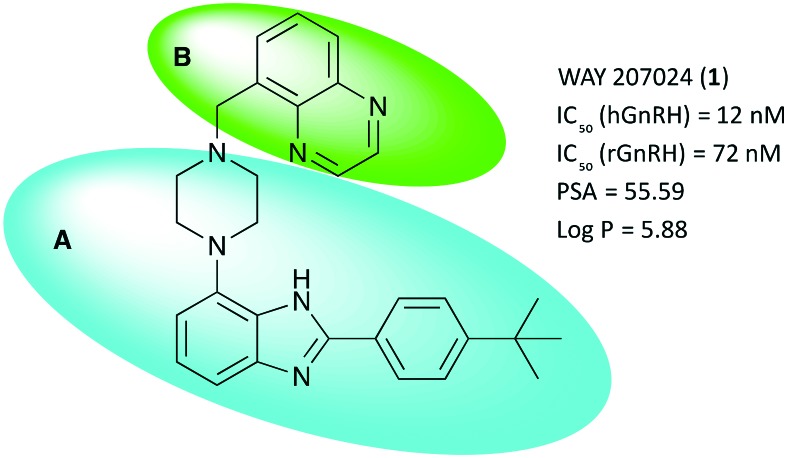
Key properties and molecular structure of WAY 207024, the lead compound for GnRH antagonist development. A (blue oval) depicts the scaffold body; B (green oval) depicts the moiety of interest for further structural optimization. IC_50_ – concentration to inhibit GnRH binding to the receptor by 50%. hGnRH – human GnRH receptor. rGnRH – rat GnRH receptor. PSA – polar surface area in Å^2^. cLog *P* – calculated octanol–water partitioning coefficient.

Based on these results, we have designed a novel library of potential antagonists addressing the following criteria:

a) A late stage diversification approach based on the 1,3-triazole motive to facilitate straightforward structural modification.

b) A moderate potency permitting competition of the ligand in circulation with endogenous GnRH for GnRHR binding sites, which would make GnRH activation subject to both GnRH concentration and the concentration of the inhibitor in tissue.

c) Improved solubility in aqueous media to improve bioavailability and pharmacokinetic profile of the lead.

Fig. 1 shows the title scaffold and some relevant properties discussed herein. As apparent from the high partitioning coefficient log *P*, the compound is highly lipophilic, which may hamper solubility in aqueous formulation as well as non-specific binding of the compound *in vivo*. In previous studies, it was found that the body of the scaffold (blue oval, A) is central to binding selectively the GnRH receptor, whereas structural modifications in the upper section of the lead (green oval, B) with planar, hydrogen bonding functional groups were found to be beneficial for modulating antagonist potency.

We surmised that the 1,3-functionalised triazole moiety would resemble well the planar geometry required for successful structural modification and simultaneously allow for introducing a broad spectrum of structural variations in the last synthetic stage using one robust reaction. Since the 1,3-functionalised triazole is obtained *via* the Cu-catalysed azide-alkyne cycloaddition reaction, we opted for working with a building block based on A to obtain an alkyne substrate for the reaction.

The overall design strategy in this communication was to generate triazoles from different functionalized azides that would be beneficial for the potency to inhibit the GnRH receptor while improving the aqueous solubility. We incorporated a variety of azides *via* click chemistry, including sugar moieties, which have been thoroughly demonstrated to effect favourable solubility characteristics and are discussed extensively in the literature.^12^–^15^ Furthermore, the reduced solubility of the alkyl and aromatic azides would also counteract our intention to improve aqueous solubility through reducing clog *P* (and hence, the bioavailability and pharmacokinetically desirable profile).

The desired intermediate (**7**) was synthesised in 19% overall yield over six steps on a multigram scale. In brief, 2,5-difluoronitrobenzene was converted to phenylene diamine **4** over three steps as described previously.^9^ Contrary to the published method, we found that the oxidative conversion of **4** into imidazole **6** proceeded smoothly in absence of a transition metal catalyst with air as the oxidant. The observation of **6b** as a minor by-product suggests a Cannizarro-like side reaction.^16^ The route is shown in Scheme 1.

**Scheme 1:**
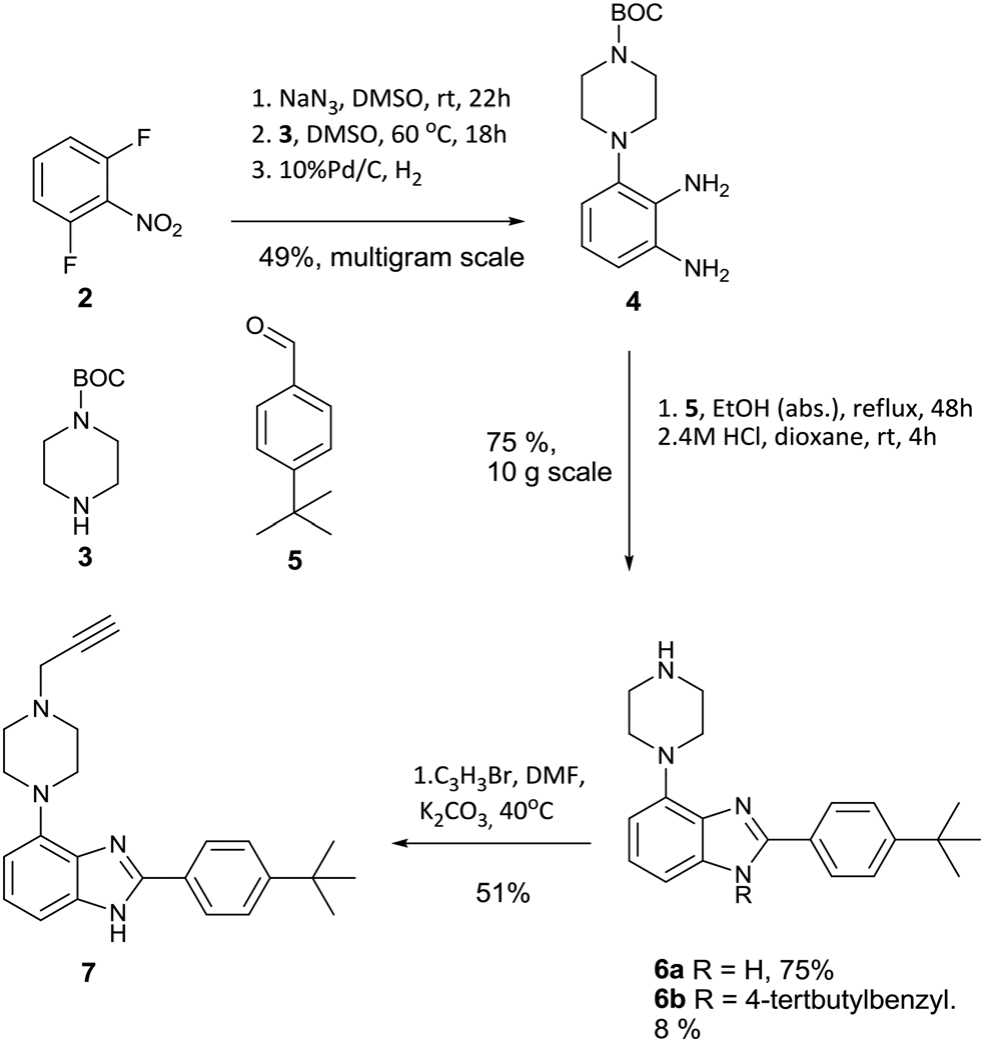
Synthetic route to alkyne intermediate **7**.

A further modification was made relative to literature to facilitate cleavage of the BOC protecting group. Substitution of the TFA used originally with HCl in dioxane gave a colourless solid instead of an oily product, which benefits the scalability of this route. Notably, compound **4** is fully converted into heterocyclic products, although the competing formation of **6b** in about 8% yield limits the overall yield of **6a** to 75% over two steps. Intermediate **7** was synthesized by alkylation with propargyl bromide in dry DMF using potassium carbonate as base in 51% yield to furnish the desired substrate **7** for azide alkyne cycloaddition.

With a robust and high yielding route to gram amounts of **7** in hand, further diversification using the copper-catalysed azide-alkyne cycloaddition (CuAAC) reaction with a range of commercially available azide substrates became possible. See Scheme 2 for an overview.

**Scheme 2:**
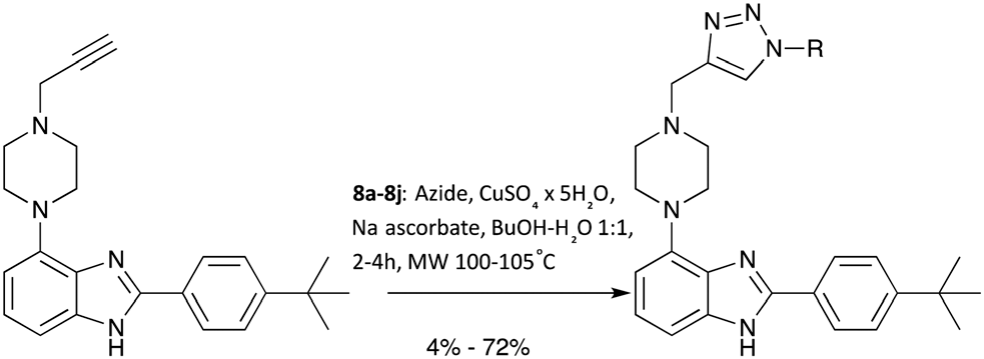
Diversification of the scaffold body using CuAAC.

Since products **8a–8j** were obtained in more than sufficient yields for biological testing following a published protocol, no attempts to optimise the reaction were made at this point.

Following synthesis and characterisation, inhibition of receptor activation by LHRH/GnRH was measured in division arrested cell lines from Multispan Inc. stably expressing functional hGnRH receptors. As described in the literature, the solubility of **1** and some of its derivatives is rather poor, which lead to a cut-off at 5 × 10^3^ nM for the maximum concentration in the cell culture assay. Compounds insoluble in DMSO at 10 mM were dropped from testing. Nonetheless, concentration dependent competition studies were performed *in vitro* in presence of 5 nM LnRH antagonist with the remainder of derivatives. Compounds showing a *p*IC_50_ > 5 were tested again over a wide range of concentrations (logarithmic, 0.1–10^3^ nM). A one-site competitive inhibition model was found to work best for fitting of the data curves. To evaluate the obtained inhibition potency values and to validate the assay, we included the well-described, commercially available GnRH antagonists T98475 (**8k**) and AG04557 (**8l**) as independent, positive controls and the metabolically stable peptide agonist buserelin as negative control. The lead compound WAY207024 (**1**) was included as a reference in the cell assay. All IC_50_ values obtained for each test compound in the concentration dependent screening were converted into *K*_i_ values using the equation of Cheng and Prusoff.^15^

As illustrated in Fig. 2, compounds derived from **1** using CuAAC were found to exhibit competitive antagonism against the endogeneous agonist GnRH. While alkyl piperazines **7a** and **7b** show only moderate potency, 21 and 43-fold lower than **1**, respectively, their clog *P* values are lower than that of **1**, which results in higher solubility (>100 nM). In line with our working hypothesis, the introduction of a planar triazole-core has a positive effect on potency. The 2-fluoroethyl triazole **8a** is equally potent as **1**, Interestingly the thermodynamic solubility assay showed that **8a** is 1.5 times more soluble than **1**, 3 μg ml^–1^ at pH 7.4 (phosphate buffered saline) and 2.098 mg mL^–1^ at pH 1.2 (simulated gastric fluid) which was indicated by the lower clog *P*-value. The introduction of an N-BOC-*Ala* functionalised triazole **8b** further improves polarity, albeit at the cost of a drop of one order of magnitude in potency. While these results may indicate that the triazole moiety is a useful pharmacophore, its vicinity was found to be much less tolerant to the introduction of pyranose moieties (**8c–f**), which was attempted to improve solubility. While these sugar derivatives lead to a major improvement of polarity as indicated by clog *P* values between 2 and 5, their potency to inhibit GnRH binding dropped out of the desired range. Masking the polyol as a tetraacetate (**8c**) helped with retaining potency, which does not bode well for making use of the solubility improvement by glycosylation. However, when varying the azide component to a synthetic glycoside to obtain **8g**, a viable compromise between solubility and inhibition potency was obtained. Derivative **8g** was measured to be soluble at pH 1.2, 1.2 mg ml^–1^ and <1 μg ml^–1^ at pH 7.4. With a potency of 38 nM, barely threefold lower than that of **1**, this hit may create an opening for further exploration using a library of diverse analogues of **8g**. Products **8h–j**, obtained from aromatic and heteroaromatic azides and **7**, did not lead to increased solubility or affinities compared to WAY207024.

**Fig. 2:**
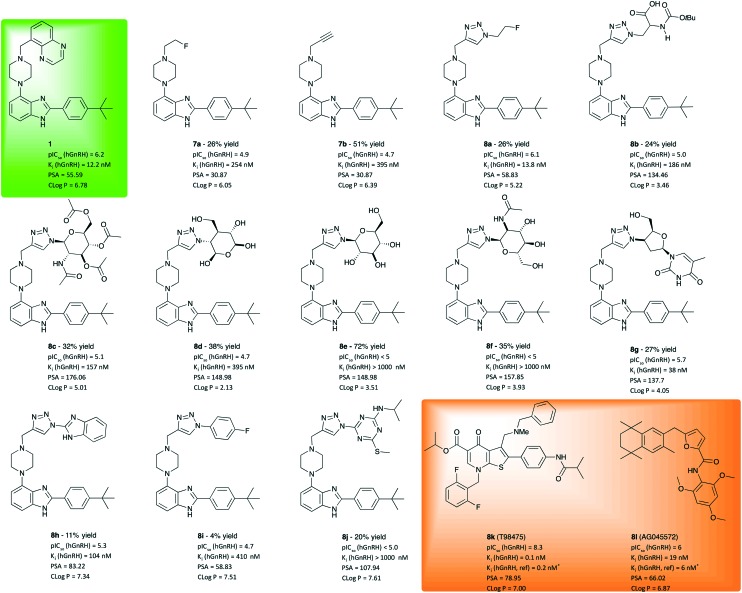
Investigated molecular entities, isolated yields, inhibition potency, inhibition constant and properties computed for evaluation. For comparison, test results of lead **1** (green box) and positive controls **8k** and **8l** should be considered. ^a^Reference value from literature.^17^,^18^ IC_50_ values are given as the geometric mean of two experiments, see ESI† for details. K_i_ was calculated from mean value using the Cheng–Prusoff equation (Ki = IC_50_/(1 + [L]/Kd)).^19^^c^Replicated twice with broader range of concentrations. ^d^Based on single experiment.

In conclusion, 18 analogues were synthesized using an approach of late-stage diversification *via* CuAAC and evaluated for potency to inhibit hGnRHR activation. While molecular diversity can easily be introduced into the scaffold body *via* this route few compounds with hGnRH inhibition potency in the desired range were identified indicating limited tolerance for structural modification in the vicinity of the triazole. The pyranose derivatives did not give the desired inhibition to the GnRH receptors neither did the less soluble compounds **8h** and **8i**. Nonetheless, compounds **8a** and **8g** emerge as highly promising candidates for further investigation in behavioural animal models as a couple of GnRH modulating antagonists.

## Conflicts of interest

The author declare no competing interests.

## Supplementary Material

Supplementary information

## References

[cit1] Meethal S., Smith M., Bowen R., Atwood C. (2005). Endocrine.

[cit2] Tobin V. A., Millar R. P., Canny B. J. (1997). Endocrinology.

[cit3] Zhang G., Li J., Purkayastha S., Tang Y., Zhang H., Yin Y., Li B., Liu G., Cai D. (2013). Nature.

[cit4] Eisenegger C., Naef M., Snozzi R., Heinrichs M., Fehr E. (2010). Nature.

[cit5] van Poppel H., Nilsson S. (2008). Urology.

[cit6] Gustofson R. L., Segars J. H., Larsen F. W. (2006). Hum. Reprod..

[cit7] Engel J. B., Schally A. V. (2007). Nat. Clin. Pract. Endocrinol. Metab..

[cit8] Briken P., Hill A., Berner W. (2003). J. Clin. Psychiatry.

[cit9] Pelletier J. C., Chengalvala M., Cottom J., Feingold I., Garrick L., Green D., Hauze D., Huselton C., Jetter J., Kao W., Kopf G. S., Lundquist J. T. T., Mann C., Mehlmann J., Rogers J., Shanno L., Wrobel J. (2008). Bioorg. Med. Chem..

[cit10] Hauze D. B., Chengalvala M. V., Cottom J. E., Feingold I. B., Garrick L., Green D. M., Huselton C., Kao W., Kees K., Lundquist Iv J. T., Mann C. W., Mehlmann J. F., Rogers J. F., Shanno L., Wrobel J., Pelletier J. C. (2009). Bioorg. Med. Chem. Lett..

[cit11] Pelletier J. C., Chengalvala M. V., Cottom J. E., Feingold I. B., Green D. M., Hauze D. B., Huselton C. A., Jetter J. W., Kopf G. S., Lundquist J. T., Magolda R. L., Mann C. W., Mehlmann J. F., Rogers J. F., Shanno L. K., Adams W. R., Tio C. O., Wrobel J. E. (2009). J. Med. Chem..

[cit12] Egleton R. D., Davis T. P. (2005). NeuroRx.

[cit13] Banerjee A., Maschauer S., Hübner H., Gmeiner P., Prante O. (2013). Bioorg. Med. Chem. Lett..

[cit14] Haubner R., Kuhnast B., Mang C., Weber W. A., Kessler H., Wester H.-J., Schwaiger M. (2004). Bioconjugate Chem..

[cit15] Schottelius M., Rau F., Reubi J. C., Schwaiger M., Wester H.-J. (2005). Bioconjugate Chem..

[cit16] Chebolu R., Kommi D. N., Kumar D., Bollineni N., Chakraborti A. K. (2012). J. Org. Chem..

[cit17] Iatsimirskaia E. A., Gregory M. L., Anderes K. L., Castillo R., Milgram K. E., Luthin D. R., Pathak V.V.
P.P., Christie L. C., Vazir H., Anderson M. B., May J. M. (2002). Pharm. Res..

[cit18] Cho N., Harada M., Imaeda T., Imada T., Matsumoto H., Hayase Y., Sasaki S., Furuya S., Suzuki N., Okubo S., Ogi K., Endo S., Onda H., Fujino M. (1998). J. Med. Chem..

[cit19] Cheng Y.-C., Prusoff W. H. (1973). Biochem. Pharmacol..

