# Supplementary material for: Small molecule piperazinyl-benzimidazole antagonists of the gonadotropin-releasing hormone (GnRH) receptor
Source: Medchemcomm. 2017 Sep 14;8(10):1965–9. doi: 10.1039/c7md00320j (PMC6072469; doi:10.1039/c7md00320j)
Supplement: Supplementary file 1 [file MD-008-C7MD00320J-s001.pdf]

Electronic Supplementary Material (ESI) for MedChemComm.
This journal is © The Royal Society of Chemistry 2017

Supporting information:

Small molecule piperazinyl-benzimidazole antagonists of the gonadotropin-releasing hormone (GnRH) receptor

Richard Fjellaksel,[a,b,c] Marc Boomgaren,[c] Rune Sundset,[a,d] Ira H. Haraldsen[e], Jørn H. Hansen,[c] Patrick J Riss[e,f,g]

[a]*Medical Imaging Group, Department of Clinical Medicine, UiT The Arctic University of Norway, 9037 Tromsø, Norway.*
[b]*Drug Transport and Delivery Group, Department of Pharmacy, UiT The Arctic University of Norway, 9037 Tromsø, Norway.*
[c]*Organic Chemistry Group, Department of Chemistry, UiT The Arctic University of Norway, 9037 Tromsø, Norway.*
[d]*PET imaging center, division of diagnostics, UNN – University Hospital of North-Norway, 9038 Tronsø, Norway.*
[e]*Department of neuropsychiatry and psychosomatic medicine, Oslo University Hospital, Oslo, Norway.*
[f]*Realomics SFI, Department of Chemistry, University of Oslo, PO BOX 1033, Oslo 0371, Norway.*
[g]*Norsk Medisinsk Syklotronsenter AS, Postboks 4950 Nydalen, 0424 Oslo*

General:
All solvents and reagents were obtained from Sigma Aldrich (Sigma-Aldrich Norway AS). Silica gel 60 TLC plates with fluroscence indicator (F254) from Merck were used. All reactions were conducted multiple times. Anhydrous conditions were conducted only if mentioned.

Instruments:
Microwave: Anton Parr Monowave 300®.
Flash Chromatography: Biotage SP1 systems ®.
HRMS: Thermo scientific LTQ Orbitrap XL + Electrospray ion source (ION-MAX) – The instrument was made at Thermo in Bremen, Germany
IR: Varian 7000e FT-IR spectrometer, Pike miracle ATR.
NMR: 400 MHz Bruker Avance III HD equipped with a 5 mm SmartProbe BB/1H (BB=19F, 31P-15N).
HPLC systems: Waters 2545 HPLC pump. Waters 2998 PDA detector, 200-500nm. Waters 2767 sample manager. XBridge ® prep C18 5µm OBD[tm] 19x250mm column.
UPLC systems: Acquity UPLC H class. Acquity column manager. Acquity PDA detector. Acquity UPLC ® BEH C18 1,7µm. 2,1x50nm column.

Determination of purity by UPLC:
Column at 50°C, the same method used for all analysis.
Method: 0,6ml/min. Initial 95% H_2O 0,1% TFA(Trifluoroacetic acid), 5% ACN(Acetonitrile) 0,1% TFA then linear change during 10 minutes to 5% H_2O 0,1% TFA, 95% Acetonitrile 0,1% TFA.

Methods used for HPLC purification:
Method A:
25ml/min. Initial 95% H_2O 0,1% TFA, 5% ACN 0,1% TFA then linear change during 15 minutes to 5% H_2O 0,1% TFA, 95% ACN 0,1% TFA.

Method B:
25ml/min. Initial 95% H_2O 0,1% TFA, 5% Acetonitrile 0,1% TFA, then linear change during 3 minutes to 70% H_2O 0,1% TFA, 30% ACN 0,1% TFA, then linear change during 7 minutes to 55% H_2O 0,1% TFA, 45% ACN 0,1% TFA.

Method C:
25ml/min. Initial 95% H_2O 0,1% TFA, 5% ACN 0,1% TFA, then linear change during 13 minutes to 5% H_2O 0,1% TFA, 95% ACN 0,1% TFA.

Method D:
25ml/min. Initial 95% H_2O 0,1% TFA, 5% ACN 0,1% TFA, then linear change during 4 minutes to 50% H_2O 0,1% TFA, 50% ACN 0,1% TFA, then linear change during 7 minutes to 5% H_2O 0,1% TFA, 95% ACN 0,1% TFA.

Method E:
25ml/min. Initial 95% H_2O 0,1% TFA, 5% ACN 0,1% TFA, then linear change during 10 minutes to 40% H_2O 0,1% TFA, 60% ACN 0,1% TFA.

Method F:
25ml/min. Initial 95% H_2O 0,1% TFA, 5% ACN 0,1% TFA, then linear change during 10 minutes to 50% H_2O 0,1% TFA, 50% ACN 0,1% TFA, then linear change during 2.1 minutes to 5% H_2O 0,1% TFA, 95% ACN 0,1% TFA.

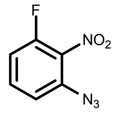

1-Azido-3-fluoro-2-nitrobenzene
4,7 g (29 mmol, 1.0 equiv.) 2,6-difluoronitrobenzene was added to a solution containing 1,9 g (29 mmol, 1.0 equiv.) of NaN_3 (sodium azide) and 15 ml DMSO. The solution was stirred for 25 hours. It was then extracted with 300 ml ethyl acetate and washed with 300 ml water for 3 times. The solution was dried over anhydrous Na_2SO_4. Then filtered and evaporated. Yellow orange oil (4,4 g 83% yield). ^{1}H-NMR (400 MHz, $CDCl_3$), δ in ppm = 7,49 (m), 7,08 (d, J = 8,3 Hz), 7,03(t), This is consistent with literature data.[1]

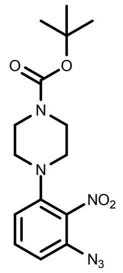

***tert*-Butyl 4-(3-azido-2-nitrophenyl)piperazine-1-carboxylate**
4,3 g (24 mmol) 1-azido-3-fluoro-2-nitrobenzene was dissolved in 20 ml DMSO and transferred to a solution containing 4,9 g (26 mmol, 1,1 equiv.) 1-Boc-piperazine, 5,3 ml (31 mmol, 1,3 equiv.) DIPEA and additional 18 ml DMSO to the total DMSO volume of 38 ml. The solution was stirred for 24 hours at 60°C, then cooled down to room temperature. The batch was divided in three parts due to the large amount of solvent. Each part was extracted with 800 ml ethyl acetate and washed one time with 200 ml diluted HCl solution (pH 3) and then 3 times more with distilled water. Brine was used if the organic layer did not separate well from the water layer. The organic layer was dried over anhydrous sodium sulfate and filtered before the solvent was concentrated under reduced pressure. The residue was dissolved in DCM, dry-mounted on celite and chromatographed by silica gel 40-63µm (VWR chemicals) (15:85 ethylacetate:pentan; R_f = 0.23). The collected fractions were concentrated under reduced pressure and dried to provide yellow-orange crystals. (5,1 g, 61 % yield).
^{1}H-NMR (400 MHz $CDCl_3$), δ in ppm = 7,43 (t, 1H), 7,00(d, J = 8.2 Hz 1H), 6,96(d, J = 8.2 Hz 1H), 3,50(m, 4H), 2,92(m, 4H), 1,49(s, 9H). This is consistent with literature data.[1]

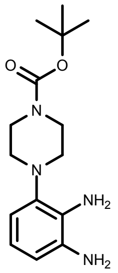

***tert*-Butyl 4-(2,3-diaminophenyl)piperazine-1-carboxylate (4)**
10 ml MeOH was added carefully to 0,125 g 10% palladium on carbon under nitrogen gas. 1,0 g (3.0 mmol) *tert*-butyl 4-(3-azido-2-nitrophenyl)piperazine-1-carboxylate was added to the solution. The solution was hydrogenated in a Parr-apparatus for 3 hours at 150 psi. Then filtered through celite. The solvent was evaporated after filtration and dried under vacuum to provide dark brown solids. (832 mg, 97% yield)
^{1}H NMR (400 MHz, $CDCl_3$), δ in ppm = 6.68 (t, 1H), 6.59 (d, J = 8.8 Hz, 1H), 6.55 (d, J = 7.6 Hz, 1H), 3.56 (br s, 8H), 2.83 (s, 4H), 1.49 (s, 9H). This is consistent with literature data.[1]

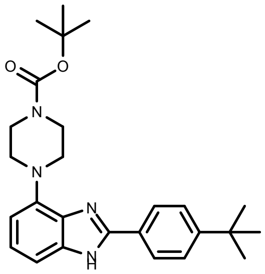

***tert*-Butyl 4-(2-(4-*tert*-butylphenyl)-1H-benzo[d]imidazol(-4-yl)piperazine-1-carboxylate**
0,3 g (1 mmol) of *tert*-butyl 4-(2,3-diaminophenyl)piperazine-1-carboxylate (**4**) was dissolved in absolute ethanol (10 ml). 0,175 ml (0,1 mmol, 1.0 equiv.) 4-*tert*-butylbenzaldehyde was added to the reaction mixture. The reaction mixture was refluxed for 24 hours at 80°C in an oil bath. The solvent was evaporated and the solids chromatographed on Biotage SP1 flash chromatography systems using a Biotage snap kp-sil 50 g column. 40 ml/min, equilibration 5% ethyl acetate in heptane for 375 ml, then 5%-40% ethyl acetate in heptane 1500 ml and 40%-95% ethyl acetate in heptane 375 ml. The fractions were collected and evaporated to provide a white powder. (684 mg, 78% yield)
^{1}H-NMR (400 MHz DMSO-d_6), δ in ppm = 12,73(s, 1H), 8,07(d, J = 8.2Hz, 2H), 7,56(d, J = 8.1 Hz 2H), 7,06(d, J = 3.7 Hz, 2H), 6,53(t, J = 4.4 Hz, 1H), 3,57(br s, 4H), 3,51(br s, 4H), 1,44(s, 9H), 1,33(s, 9H). MS (HRMS (ESI):m/z Found 435,2753 $[M+H]^+$ Calculated 435,2755. This is consistent with literature data.[1]

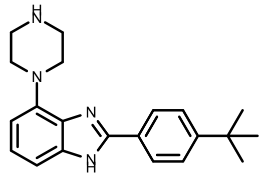

2-(4-*tert*-Butylphenyl)-4-(piperazin-1-yl)-1H-benzo[d]imidazole (6a)

655mg (1,5 mmol) of *tert*-Butyl 4-(2-(4-*tert*-butylphenyl)-1H-benzo[d]imidazol(-4-yl)piperazine-1-carboxylate was dissolved in 10ml 4M HCl in dioxane. The reaction was left for 5 hours. HCl and dioxane were removed by reduced pressure. Then chromatographed on Biotage SP1 flash chromatography systems by a Biotage snap ultra C18rp 12g column. 15 ml/min. Initial 5% acetonitrile and 95% H_2O, then linear change during 20 minutes to 5% H_20, 95% acetonitrile. The solvents were evaporated to obtain the product as white powder. (489 mg, 96 % yield) Purity: 97,2 % ^{1}H-NMR (400 MHz DMSO-d_6), δ in ppm = 9,67 (br s 1H), 8,49(d, *J* = 8.3 Hz, 2H), 7,71(d, *J* = 8.2 Hz, 2H), 7,53(d, *J* = 8.1 Hz, 1H), 7,48(t, *J* = 7.9 Hz, 1H), 7,11(d, 7.6Hz, 1H), 3,38(m, 8H), 1,33(s, 9H). ^{13}C-NMR (100 MHz DMSO-d6), δ in ppm=156.5, 148.9, 139.5, 132.9, 129.2, 126.7, 126.1, 125.4, 120.2, 114.0, 108.4, 47.8, 42.7, 35.1, 30.8 IR (Neat): 2955, 2689, 2466, 1622, 1459, 1383 HRMS(ESI): m/z Found 335,2236 [M+H]$^+$. Calculated 335,2236. This is consistent with literature data.[1]

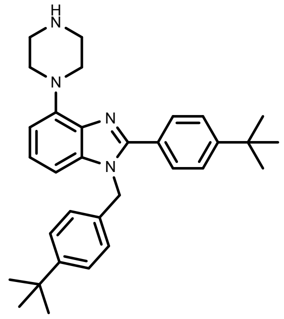

1-(4-*tert*-Butylbenzyl)-2-(4-*tert*-butylphenyl)-4-(piperazin-1-yl)-1H-benzo[d]imidazole (6b)

1,4 g of *tert*-butyl 4-(1-(4-*tert*-butylbenzyl)-2-(4-*tert*-butylphenyl)-1H-benzo[d]imidazol-4-yl)piperazine-1-carboxylate **(5b)** was dissolved in 25 ml 4M HCl in dioxane. The reaction was left for 5 hours. The solvents were evaporated to obtain the product as white powder. (48 mg, 58 % yield). Purity: 97.2 %.

^{1}H-NMR (400 MHz, $CDCl_3$), δ in ppm = 9.70 (br s 1H), 7.64 (d, *J* = 8.0 Hz, 2H), 7.47 (d, *J* = 8.0 Hz, 2H), 7.33 (d, *J* = 8.0 Hz, 1H), 7.13 (t, *J* = 7.9 Hz 1H), 7.02 (d, *J* = 7.9 Hz, 1H), 6.87 (d, *J* = 8.1 Hz, 1H), 6.66 (d, *J* = 7.8 Hz, 1H) 5.41((s, 2H), 3.92(s, 4H), 3.49(s, 4H), 1.32(s, 9H), 1.26(s, 9H) ^{13}C-NMR (100 MHz, $CDCl_3$) δ in ppm=153.2, 125.3, 150.8, 141.8, 137.6, 135.3, 133.5, 129.2, 127.3, 123.7, 108.2, 105.1, 48.4, 47.1, 43.8, 35.0, 34.7, 31.5, 31.4 IR (Neat):2957, 2858, 2488, 1701, 1202 HRMS (ESI) m/z: Found 481.3334 [M+H]$^+$. Calculated: 481.3331.

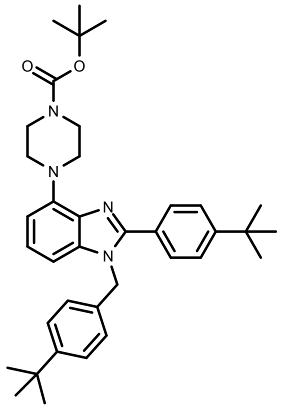

***tert*-Butyl 4-(1-(4-tert-butylbenzyl)-2-(4-*tert*-butylphenyl)-1H-benzo[d]imidazol-4-yl)piperazine-1-carboxylate (5b)**

0,3 g (1 mmol, 1.0 equiv.) of *tert*-butyl 4-(2,3-diaminophenyl)piperazine-1-carboxylate **(4)** was dissolved in absolute ethanol (10 ml). 0,16 ml (1 mmol, 1.0 equiv.) of *tert*-butylbenzaldehyde was added and the reaction mixture was refluxed for 27 hours. The reaction mixture was evaporated and chromatographed on Biotage SP1 flash chromatography systems by a Biotage snap kp-sil 50 g column. . Method: 40 ml/min, initial 5% ethyl acetate and 95% heptane, then linear change during 37.5 minutes to 60 % heptane 40 % ethyl acetate. Then linear change during 9.4 minutes to 5% heptane, 95% ethyl acetate. The solvents were evaporated to obtain the product as white powder. (77 mg 13% yield). Purity: 95,8 %. ^{1}H-NMR (400 MHz , $CDCl_3$), δ in ppm = 7.66 (d, *J* = 8.2 Hz, 2H), 7.46 (d, *J* = 8.2 Hz, 2H), 7.32 (d, *J* = 8.1 Hz, 2H), 7.11 (t, *J* = 7.9 Hz. 1H), 7.03 (d, *J* = 8.1 Hz, 2H), 6.81 (d, *J* = 8.0 Hz, 1H), 6.65 (d, *J* = 7.8 Hz, 1H).5,40(s, 2H), 3,72(m, 4H), 3,58(m, 4H), 1,50(s, 9H), 1,33(s, 9H), 1,30(s, 9H) ^{13}C-NMR (100 MHz , $CDCl_3$), δ in ppm=155.0, 153.0, 151.8, 150.7, 143,6, 137.6, 135.5, 133.7, 129.2, 127.6, 126.0, 125.8, 125.8, 123.6, 108.0, 104.0, 79.8, 50.1, 48.3, 35.0, 34.7, 31.5, 31.4, 28.6 IR (Neat): 2966, 2854, 1692, 1421, 1241 HRMS (ESI) m/z: Found 581,3866 [M+H]$^+$. Calculated 581,3856.

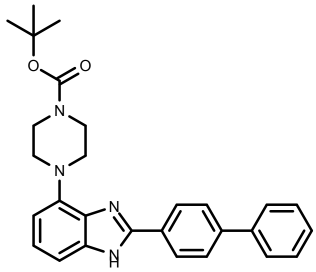

tert-Butyl 4-(2-(biphenyl-4-yl)-1H-benzo[d]imidazol-4-yl)piperazine-1-carboxylate (5c)

0,2 g (0,7 mmol, 1.0 equiv.) of *tert*-butyl 4-(2,3-diaminophenyl)piperazine-1-carboxylate (4) was dissolved in 10 ml absolute ethanol. 0,125 g (0,7 mmol, 1.0 equiv.) of 4-phenylbenzaldehyde was added to the solution. The reaction mixture was refluxed for 48 hours. Monitored by TLC. The reaction mixture was evaporated and chromatographed on Biotage SP1 flash chromatography systems by a Biotage snap kp-sil 50 g column. Method: 40 ml/min, initial 5% ethyl acetate and 95% heptane, linear change during 37.5 minutes to 60 % heptane 40 % ethyl acetate. Linear change during 9.4 minutes to and 5 % heptane, 95 % ethyl acetate. The solvents were evaporated to obtain the product as white powder. (217 mg, 70 % yield). Purity: 95.6 ^{1}H-NMR (400 MHz DMSO-d_6), δ in ppm = 12.87(s, 1H), 8.25 (d, *J* = 7.9 Hz, 2H), 7.86 (d, *J* = 8.0 Hz, 2H), 7.76 (d, *J* = 7.6 Hz, 2H), 7.51 (t, *J* = 7.51 Hz, 2H), 7.45 – 7.35 (m, 1H), 7.09 (d, *J* = 4.3 Hz, 2H), 6.55 (t, 1H), 3.58 (br s, 4H), 3.54 (br s, 4H). 1.43(s, 9H). ^{13}C-NMR (100 MHz DMSO-d_6), δ in ppm = 153.9, 148.0, 142.6, 140.9, 139.3, 136.2, 135.5, 129.2, 129.0, 127.8, 127.2, 127.1, 126.8, 126.7, 123.4, 106.7, 103.9, 78.9, 49.1, 28.1 IR (neat): 1695, 1416, 1239, 1164 HRMS (ESI) m/z: Found 455.2441 [M+H]$^{+}$. Calculated: 455.2442.

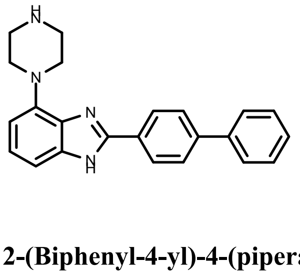

2-(Biphenyl-4-yl)-4-(piperazin-1-yl)-1H-benzo[d]imidazole (6c)

217 mg was dissolved in 4 ml 4M HCl in dioxane and the reaction was left for 5 hours and monitored by TLC. Then chromatographed on Biotage SP1 flash chromatography systems by a Biotage snap ultra C18rp 12 g column. 15 ml/min. Initial 5% acetonitrile and 95% H_2O, then linear change during 20 minutes to 5% H_20, 95% acetonitrile. The solvents were removed by evaporation and gave a white powder. (152 mg, 90 % yield). Purity: 99,5 %. ^{1}H-NMR (400 MHz DMSO-d6), δ in ppm = 9.63 (br s 1H), 8.63 (d, *J* = 8.2 Hz, 2H), 8.03 (d, *J* = 8.1 Hz, 2H), 7.85 (d, *J* = 7.6 Hz, 2H), 7.59 – 7.40 (m, 5H), 7.14 (d, *J* = 7.7 Hz, 1H) 3,43(br s, 8H). ^{13}C-NMR (100 MHz DMSO-d6), δ in ppm = 149.04, 144.80, 139.99, 138.87, 133.59, 130.24, 129.63, 129.14, 127.70, 127.50, 127.20, 126.20, 122.48, 114.33, 108.79, 43.19 IR (neat): 2623, 1623, 1611, 1433, 1376 HRMS (ESI) m/z: Found 355.1914 [M+H]$^{+}$. Calculated 355.1923.

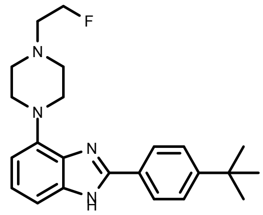

2-(4-(_tert_-Butylphenyl)-4-(4-(2-fluoroethyl)piperazin-1-yl)-1H-benzo[d]imidazole (7a)

40 mg (0.1 mmol, 1 eq) of 2-(4-*tert*-butylphenyl)-4-(piperazin-1-yl)-1H-benzo[d]imidazole (6a) was dissolved in 8 ml acetonitrile, 27 mg K_2CO_3 (1.0 equiv., 0,1 mmol) and 31 mg (1.2 equiv., 0,2 mmol) of 2-fluoroethyl tosylate was added to the reaction mixture. It was then heated to 60°C and left for 48 hours stirring. The solvent was evaporated, purified by HPLC (method F) and then lyophilized to give a white powder. (12 mg, 26% yield) Purity: 96.7 %. ^{1}H-NMR (400 MHz Methanol-d_4), δ in ppm = 8.08 (d, *J* = 8.1 Hz, 2H), 7.69 (d, *J* = 8.0 Hz, 2H), 7.40 (d, *J* = 5.7 Hz, 2H), 7.03 (d, *J* = 6.5 Hz, 1H), 5.00(m, 2H), 3.92(br s, 4H), 3.73(Br s, 4H), 1.40(s, 9H). ^{13}C-NMR (100MHz Methanol-d_4), δ in ppm = 157.0, 151.9, 141.0, 136.5, 128.6, 127.5, 126.6, 124.8, 113.1, 109.2, 80.0, 78.3, 58.0, 57.8, 53.7, 36.0, 31.5 IR (neat): 2960, 2824, 1672, 1591, 1436, 1237 HRMS (ESI) m/z; Found 381.2439 [M+H]$^{+}$. Calculated 381.2449.

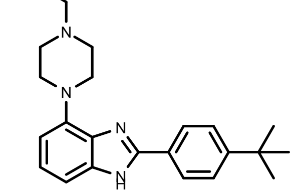

2-(4-(_tert_-Butyl)phenyl)-4-(4-(prop-2-yn-1-yl)piperazin-1-yl)-1H-benzo[d]imidazole (7b)

500 mg (1,4 mmol , 1.0 equiv.) of *tert*-butyl 4-(2-(4-*tert*-butylphenyl)-1H-benzo[d]imidazol(-4-yl)piperazine **(6a)** was dissolved in 25 ml dry DMF. 466 mg (3,4 mmol, 2,5 equiv.) of K_2CO_3 and 0,15 ml (1,4 mmol, l,1 equiv.) added to the solution. The reaction mixture was stirred for 48h at 40°C. The reaction mixture was evaporated and chromatographed on Biotage SP1 flash chromatography systems by a Biotage snap ultra C18 rp 12 g column. 15 ml/min. Initial 5% acetonitrile and 95% H_2O, then linear change during 20 minutes to 5% H_20, 95% acetonitrile. The solvents were evaporated to obtain the product as white powder. (254 mg 51 % yield). Purity: 97,4 %. ^{1}H-NMR (400 MHz DMSO-*d6*), δ in ppm = 12,69 (s, 1H), 8.06 (d, *J* = 8.3 Hz, 2H), 7.55 (d, *J* = 8.4 Hz, 2H), 7.03 (s, 1H), 6.50 (d, *J* = 6.7 Hz, 1H), 3,58(Br s, 4H), 3,20(s, 1H) 2,70(Br s, 4H), 1,33(s, 9H).) ^{13}C-NMR (100MHz DMSO-*d6*), δ in ppm = 152.1, 148.1, 142.7, 136.1, 135.3, 127.6, 126.0, 125.6, 123.1, 106.3, 103.4, 79.4, 75.8, 51.2, 48.9, 46.2, 34.5, 31.0 IR (Neat): 3298, 2960, 2822, 1590, 1435, 1239 HRMS (ESI) m/z; Found 373,2399 $[M+H]^+$. Calculated 373.2398.

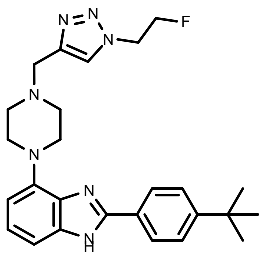

2-(4-*tert*-Butylphenyl)-4-(4-((1-(2-fluoroethyl)-1H-1,2,3-triazol-4-yl)methyl)piperazin-1-yl)-1H-benzo[d]imidazole (8a)

40 mg (1 eq) of 2-(4-(*tert*-butyl)phenyl)-4-(4-(prop-2-yn-1-yl)piperazin-1-yl)-1H-benzo[d]imidazole **(6)** was added to a solution of Cu(II)sulphate pentahydrate (0,2 equiv.) and sodium ascorbate (0,4 equiv.) in 5 ml 1:1 distilled H_2O and *tert*-butanol. 10 mg of 2-fluoroethyl azide (28 mg/ml solution) added. The reaction mixture heated and stirred by microwave for 2 hours, 100°C. The solvents were then evaporated, dissolved in 2 ml of methanol with some drops of DMSO and purified by HPLC (Method C). The product was lyophilized to obtain a white powder. (13 mg, 26% yield). Purity: 95.8 % ^{1}H-NMR (400 MHz Methanol-d_4), δ in ppm = 8.30 (s, 1H), 8.07(d, *J* = 7.9 Hz, 2H), 7.73(d, *J* = 8.0 Hz, 2H), 7,46(s, 2H), 7,14(s,1H), 4,79(s,2H), 4,64(s,2H), 3,67(br-s,8H), 1,40(s,9H)). ^{13}C NMR (100 MHz, Methanol-d_4) δ in ppm = 156.8, 150.3, 139.1, 136.2, 134.0, 127.7, 127.3, 126.4, 126.2, 121.6, 113.4, 108.4, 82.2 (^{1}JCF 169 Hz), 80.5(^{1}JCF 169 Hz), 51.4, 50.8, 50.6 (^{2}JCF 38 Hz), 50.2 (^{2}JCF 38 Hz), 34.8, 30.0 IR (neat): 2966, 1665, 1198, 1181, 1127 HRMS ESI: m/z 462,2783 [M+H]+ Calculated: 462,2781.

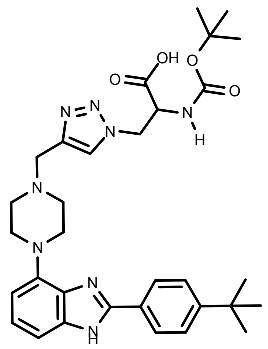

2-(*tert*-Butoxycarbonylamino)-3-(4-((4-(2-(4-*tert*-butylphenyl)-1H-benzo[d]imidazol-4-yl)piperazin-1-yl)methyl)-1H-1,2,3-triazol-1-yl)propanoic acid (8b)

50 mg (1 eq, 0,1 mmol) of 2-(4-(*tert*-butyl)phenyl)-4-(4-(prop-2-yn-1-yl)piperazin-1-yl)-1H-benzo[d]imidazole **(6)** was added to a solution of 3,4 mg of Cu(II)sulphate pentahydrate (0,2 equiv.) and 12 mg of sodium ascorbate (0,4 equiv.) in 5 ml 1:1 distilled H_2O and *tert*-butanol. 52 mg (1 equiv., 0,1 mmol) of boc-3-azido-Ala-OH dicycloammonium salt was added. The reaction was done in a microwave for 2 hours at 100°C. The solvents were then evaporated, dissolved in 2ml methanol and purified by HPLC (Method A). The product was lyophilized to obtain a white powder. (19 mg, 24% yield). Purity: 95.6 % ^{1}H-NMR (400MHz Methanol-d_4), δ in ppm = 8.23(s, 1H), 8.06 (d, *J* = 8.4 Hz, 2H), 7.71 (d, *J* = 8.6 Hz, 2H), 7.44 (d, *J* = 4.3 Hz, 2H), 7.10 (t, 1H)., 4.95(m, 1H), 4.77(m,1H), 4.63(br-s,3H), 3.62(Br s, 8H), 1.38(s,9H) ^{13}C NMR (100 MHz, Methanol-d_4) δ in ppm = 172.1, 158.0, 157.7, 151.7, 140.5, 137.3, 135.5, 129.0, 127.8, 127.5, 123.3, 114.6, 109.7, 81.0, 55.2, 52.6, 52.3, 51.6, 36.1, 31.4, 28.6 IR (neat): 2968, 1664, 1183 HRMS ESI m/z: Found 603.3413 $[M+H]^+$ Calculated: 603.3407.

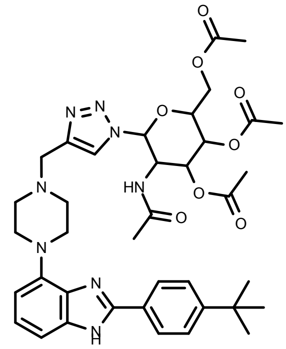

5-Acetamido-2-(acetoxymethyl)-6-(4-((4-(2-(4-*tert*-butylphenyl)-1H-benzo[d]imidazol-4-yl)piperazin-1-yl)methyl)-1H-1,2,3-triazol-1-yl)tetrahydro-2H-pyran-3,4-diyl diacetate (8c) 60 mg (1 eq, 0,2 mmol) of 2-(4-(*tert*-butyl)phenyl)-4-(4-(prop-2-yn-1-yl)piperazin-1-yl)-1H-benzo[d]imidazole **(7b)** was added to a solution of 5 mg of Cu(II)sulphate pentahydrate (0,2 equiv.) and 18 mg of sodium ascorbate (0,4 equiv.) in 5ml 1:1 distilled H_2O and *tert*-butanol. 60 mg (1 equiv., 0,16 mmol) of 2-acetamido-2-deoxy-B-D-glucopyranosol azide 3,4,6 triacetate was then added. The reaction was done in a microwave for 2 hours at 100°C. The solvents were then evaporated, dissolved in 2 ml methanol and purified by HPLC (Method E). The product was lyophilized to obtain a white powder. (38.1 mg 32% yield). Purity: 95.0 % ^{1}H-NMR (400MHz Methanol-d_4), δ in ppm = 8.57(s, 1H), 8.07(d, J = 9.0 Hz, 2H), 7.71(d, J = 4.1 Hz, 2H), 7.43(d, J = 3.6 Hz, 2H), 7.09(t, J = 3.8 Hz, 1H), 6.08(d, J = 9.8 Hz, 1H), 5.44(t, J = 10.6 Hz, 1H), 5.25(t, J = 9.7 Hz, 1H), 4.71 – 4.51(m, 3H), 4.35(dd, J = 12.7, 5.0 Hz, 1H), 4.26 – 4.07(m, 2H), 3.62(br s, 8H), 2.05(s, 3H), 2.03(s, 3H), 1.97(s, 3H), 1.71(s, 3H), 1.40(s, 9H)^{13}C NMR (100 MHz, Methanol-d_4) δ in ppm = 173.5, 172.1, 171.6, 171.2, 157.7, 151.7, 140.6, 137.9, 135.9, 130.3, 128.9, 127.7, 127.2, 127.0, 123.7, 114.2, 109.6, 87.7, 76.2, 73.5, 69.5, 63.1, IR (neat): 2962, 1667, 1200 HRMS ESI m/z: Found 745.3668 [M+H]$^+$ Calculated: 745.3668

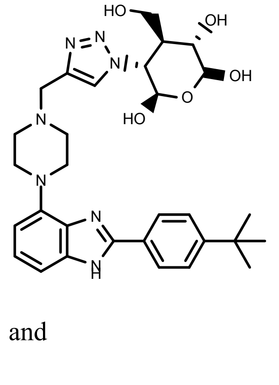

and

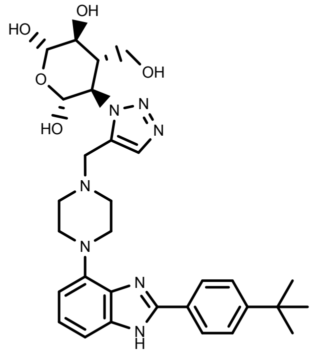

Regioisomeric mixture of (2S,3S,4S)-5-(4-((4-(2-(4-t*ert*-butylphenyl)-1H-benzo[d]imidazol-4-yl)piperazin-1-yl)methyl)-1H-1,2,3-triazol-1-yl)-4-(hydroxymethyl)tetrahydro-2H-pyran-2,3,6-triol (8d) and (2S,3S,4S)-5-(5-((4-(2-(4-tert-butylphenyl)-1H-benzo[d]imidazol-4-yl)piperazin-1-yl)methyl)-1H-1,2,3-triazol-1-yl)-4-(hydroxymethyl)tetrahydro-2H-pyran-2,3,6-triol (8d)

20 mg (1.0 equiv., 0,05 mmol) of 2-(4-(*tert*-butyl)phenyl)-4-(4-(prop-2-yn-1-yl)piperazin-1-yl)-1H-benzo[d]imidazole **(7b)** was added to a solution of 1,7 mg of Cu(II)sulphate pentahydrate (0,2 equiv.) and 6 mg of sodium ascorbate (0,4 equiv.) in 2,5 ml 1:1 destilled H_2O and *tert*-butanol. 11 mg (1.0 equiv., 0,05 mmol) of 2-azido-2-deoxy-D-glucose was added. The reaction was done in a microwave for 2 hours at 100°C. The solvents were then evaporated, dissolved in 2 ml methanol and purified by HPLC (Method B). The product was lyophilized to obtain a white powder. (12 mg, 38% yield). Regioisomeric triazole ratio 1:1.4 Purity = 99.2 % ^{1}H-NMR (400MHz, Methanol-d_4), δ in ppm = 8.42(d, 0.45H), 8.29(s, 0.64H), 8.07 (d, J = 7.8 Hz, 2H), 7.73 (d, J = 8.0 Hz, 2H), 7.47 (s, 2H), 7.14 (s, 1H), 5.33 (s, 0,53H), 5.16 (d, J = 7.5 Hz, 0,87H, 4.65(s, 2H), 4.23(m, 2H), 3.69(m, 11H), 1.40(s, 9H) ^{13}C NMR (100 MHz, Methanol-d_4) δ in ppm = 158.1, 151.7, 140.5, 137.0, 136.7, 135.5, 129.4, 129.0, 127.8, 127.6, 123.1, 114.8, 109.8, 96.2, 92.6, 78.3, 75.6, 73.4, 72.4, 72.1, 72.0, 69.9, 67.1, 62.6, 62.4, 52.7, 51.7, 36.1, 31.4 IR (neat): 2960, 1667, 1461, 1184, 1129 HRMS ESI m/z: Found 578.3082 [M+H]$^+$ Calculated 578.3091.

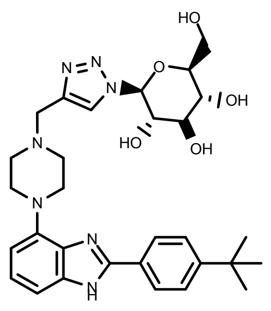

(6S)-2-(4-((4-(2-(4-*tert*-butylphenyl)-1H-benzo[d]imidazol-4-yl)piperazin-1-yl)methyl)-1H-1,2,3-triazol-1-yl)-6-

(hydroxymethyl)tetrahydro-2H-pyran-3,4,5-triol (8e)

40 mg (1 eq, 0,1 mmol) of 2-(4-(*tert*-butyl)phenyl)-4-(4-(prop-2-yn-1-yl)piperazin-1-yl)-1H-benzo[d]imidazole **(7b)** was added to a solution of 3,4 mg of Cu(II)sulphate pentahydrate (0,2 equiv.) and 12 mg of sodium ascorbate (0,4 equiv.) in 5 ml 1:1 distilled H_2O and *tert*-butanol. 22 mg (1.0 equiv., 0,1 mmol) of 1-azido-1-deoxy-β-D-glycopyranoside added. The reaction was done in a microwave for 2 hours at 100°C. The solvents were evaporated, dissolved in 2 ml methanol and purified by HPLC (Method A). The product was lyophilized to obtain a white powder. (45 mg, 72% yield). Purity: 96.6 % ^{1}H-NMR (400MHz, Methanol-d_4), δ in ppm = 8.50(s, 1H), 8.07(d, *J* = 8.8 Hz, 2H), 7.73(d, *J* = 8.7 Hz, 2H), 7.46(d, *J* = 4.1 Hz, 2H), 7.12 (t, *J* = 4.4 Hz, 1H), 5.71 (d, *J* = 9.2 Hz, 1H), 4.68(s, 2H), 3.96 – 3.87(m, 2H), 3.82 – 3.41 (m, 12H), 1.40(s, 9H), ^{13}C NMR (100 MHz, Methanol-d_4) δ in ppm = 157.9, 151.7, 140.6, 137.5, 135.6, 128.9, 127.7, 127.5, 127.4, 123.4, 114.4, 109.7, 89.7, 81.2, 78.4, 74.1, 71.0, 62.3, 52.7, 51.6, 36.1, 31.4 IR (neat): 3254, 2955, 1668, 1195, 1128 HRMS ESI m/z: Found 578.3083 [M+H]$^+$ Calculated 578.3085.

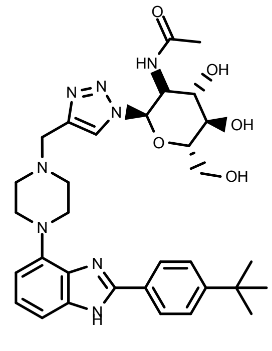

N-((2R,3S,4S,5R,6S)-2-(4-((4-(2-(4-*tert*-butylphenyl)-1H-benzo[d]imidazol-4-yl)piperazin-1-yl)methyl)-1H-1,2,3-triazol-1-yl)-4,5-dihydroxy-6-(hydroxymethyl)tetrahydro-2H-pyran-3-yl)acetamide (8f)

20 mg (1.0 equiv., 0,1 mmol) of 2-(4-(*tert*-butyl)phenyl)-4-(4-(prop-2-yn-1-yl)piperazin-1-yl)-1H-benzo[d]imidazole **(7b)** was added to a solution of 1,7 mg of Cu(II)sulphate pentahydrate (0,2 equiv.) and 6 mg of sodium ascorbate (0,4 equiv.) in 2,5 ml 1:1 destilled H_2O and *tert*-butanol 13 mg (1 equiv., 0,1 mmol) of 2-acetamido-2-deoxy-β-D-glycopyranoside azide added. The reaction was done in a microwave for 2 hours at 100°C. The solvents were evaporated, dissolved in 2 ml methanol and purified by HPLC (Method B). The product was lyophilized to obtain a white powder. (12 mg, 35% yield). Purity: 98.7 % ^{1}H-NMR (400MHz Methanol-d_4), δ in ppm=8.58(s, 1H), 8.07 (d, *J* = 8.1 Hz, 2H), 7.73 (d, *J* = 8.0 Hz, 2H), 7.46 (s, 2H), 7.13 (s, 1H), 5.80 (d, *J* = 9.9 Hz, 1H), 4.65 (s, 2H), 4.25 (t, *J* = 9.9 Hz, 1H), 3.94 (d, *J* = 11.9 Hz, 1H), 3.82 (d, *J* = 12.0 Hz, 2H), 3.64(m, 8H), 1.74 (s, 3H), 1.41(s, 9H)). ^{13}C NMR (100 MHz, Methanol-d_4) δ in ppm=173.9, 157.4, 151.8, 140.7, 137.4, 136.3, 128.7, 127.7, 127.0, 126.9, 124.2, 113.8, 109.6, 88.8, 81.3, 75.4, 71.3, 62.2, 57.4, 52.4, 51.6, 36.1, 31.5, 22.6 IR (neat): 2966, 1668, 1463, 1200, 1128 HRMS ESI m/z: Found 619.3350 [M+H]$^+$ Calculated 619.3351.

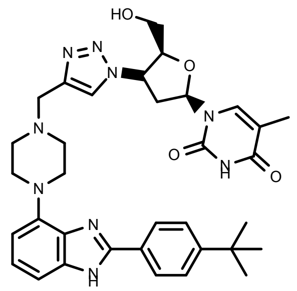

1-((2R,4R,5S)-4-(4-((4-(2-(4-*tert*-butylphenyl)-1H-benzo[d]imidazol-4-yl)piperazin-1-yl)methyl)-1H-1,2,3-triazol-1-yl)-5-(hydroxymethyl)tetrahydrofuran-2-yl)-5-methylpyrimidine-2,4(1H,3H)-dione (8g)

40 mg (1 eq, 0,1 mmol) of 2-(4-(*tert*-butyl)phenyl)-4-(4-(prop-2-yn-1-yl)piperazin-1-yl)-1H-benzo[d]imidazole **(7b)** was added to a solution of 3,4 mg of Cu(II)sulphate pentahydrate (0,2 equiv.) and 12 mg of sodium ascorbate (0,4 equiv.) in 5 ml 1:1 distilled H_2O and *tert*-butanol. 24 mg (1.0 equiv., 0,1 mmol) of Zidovudine® was added. The reaction was done in a microwave for 2 hours at 100°C. The solvents were then evaporated, dissolved in 2 ml methanol and purified by HPLC (Method B). The product was lyophilized to obtain a white powder. (19 mg, 27 % yield). Purity: 97.0 % ^{1}H-NMR (400MHz, Methanol-d_4), δ in ppm = 8.38(s, 1H), 8.06 (d, *J* = 8.1 Hz, 2H), 7.92 (s, 1H), 7.71 (d, *J* = 8.2 Hz, 2H), 7.43 (d, *J* = 4.2 Hz, 2H), 7.09 (t, *J* = 4.6 Hz, 1H), 6.53 (t, *J* = 6.6 Hz, 1H), 5.51(m, 1H), 6.65(s, 2H), 4.41(s, 1H), 3.91(m, 1H), 3.81(m, 2H), 3.67(Br s, 8H), 2.92(m, 1H), 2.77(m, 2H), 1.91(s, 3H), 1.40(s,9H)). ^{13}C NMR (100 MHz, Methanol-d_4) δ in ppm = 164.9, 155.9, 150.9, 150.4, 139.5, 136.7, 136.3, 134.8, 127.3, 126.5, 126.2, 125.5, 123.0, 112.2, 110.4, 107.9, 85.4, 85.0, 61.0, 60.4, 51.4, 50.3, 37.7, 34.6, 30.1, 11.1 IR (neat): 1669, 1461, 1200, 1128 HRMS ESI m/z: Found 640.3354 [M+H]$^+$ Calculated 640.3360.

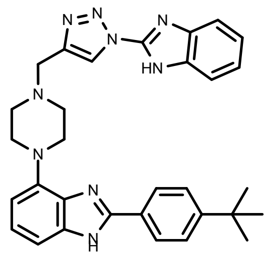

4-(4-((1-(1H-benzo[d]imidazol-2-yl)-1H-1,2,3-triazol-4-yl)methyl)piperazin-1-yl)-2-(4-*tert*-butylphenyl)-1H-benzo[d]imidazole (8h)

70 mg (1.0, 0,2 mmol) of 2-(4-(*tert*-butyl)phenyl)-4-(4-(prop-2-yn-1-yl)piperazin-1-yl)-1H-benzo[d]imidazole **(6)** added to a solution of 6 mg of Cu(II)sulphate pentahydrate (0,2 equiv.) and 15 mg of sodium ascorbate (0,4 equiv.) in 8,75 ml 1:1 destilled H_2O and *tert*-butanol. 30 mg (1.0 equiv., 0,2 mmol) of 2-azido-1-H-benzimidazole was added. The reaction was done in a microwave for 4 hours at 105°C. The solvents were evaporated, dissolved in 2 ml methanol and purified by HPLC (Method A). The product was lyophilized to obtain a white powder. (11 mg, 11% yield). Purity: 97.7 % ^{1}H-NMR (400MHz, Methanol-d_4), δ in ppm = 9.02 (s, 1H), 8.08 (d, J = 8.2 Hz, 2H), 7.74 (d, J = 8.3 Hz, 2H), 7.67 (dd, J = 6.1, 3.2 Hz, 2H), 7.48 (d, J = 3.3 Hz, 2H), 7.37 (dd, J = 6.2, 3.1 Hz, 2H), 7.16 (dd, J = 5.4, 3.3 Hz, 1H), 4.81(s, 2H), 3.75(m, 8H), 1.41(s,9H) ^{13}C NMR (100 MHz, Methanol-d_4) δ in ppm =158.0, 151.7, 143.9, 140.6, 138.8, 135.6, 129.0, 127.7, 127.5, 126.3, 124.9, 123.3, 116.7, 114.5, 109.7, 53.0, 51.4, 36.1, 31.4 IR (neat): 2969, 1668, 1465, 1198, 1130 HRMS ESI m/z: Found: 532.2931 [M+H]+ Calculated: 532.2937

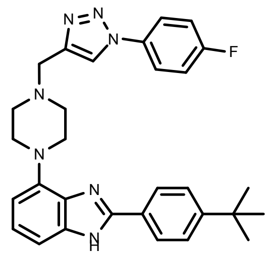

2-(4-*tert*-Butylphenyl)-4-(4-((1-(4-fluorophenyl)-1H-1,2,3-triazol-4-yl)methyl)piperazin-1-yl)-1H-benzo[d]imidazole (8i)

70 mg (1.0 equiv., 0,2 mmol) of 2-(4-(*tert*-butyl)phenyl)-4-(4-(prop-2-yn-1-yl)piperazin-1-yl)-1H-benzo[d]imidazole **(7b)** added to a solution of 6 mg of Cu(II)sulphate pentahydrate (0,2 equiv.) and 15 mg of sodium ascorbate (0,4 equiv.) in 8,75 ml 1:1 destilled H_2O and *tert*-butanol. 22µl (1.0 equiv., 0,2 mmol) of 2-azido-1-H-benzimidazole added. The reaction was done in a microwave for 2 hours at 100°C. The solvents were then evaporated, dissolved in 2 ml 1:1 Methanol:DMSO and purified by HPLC (Method A). The product was lyophilized to obtain a white powder. (4 mg, 4% yield). Purity: 99.0 % ^{1}H-NMR (400MHz, Methanol-d_4), δ in ppm = 8.78 (s, 1H), 8.08 (d, J = 8.3 Hz, 2H), 7.93 (dd, J = 8.9, 4.5 Hz, 2H), 7.73 (d, J = 8.3 Hz, 2H), 7.47 (d, J = 3.4 Hz, 2H), 7.38 (t, J = 8.6 Hz, 2H), 7.15 (dd, J = 5.2, 3.4 Hz, 1H), 4.73(s, 2H), 3.73(br s, 8H), 1.40(s,9H) ^{13}C NMR (100 MHz, Methanol-d_4) δ in ppm = 165.5 (^{1}JCF 247 Hz), 163.0(^{1}JCF 247 Hz) , 158.1, 151.7, 140.6, 138.4, 135.5, 134.5, 129.0, 127.8, 127.6, 126.6, 124.1(^{3}JCF 8 Hz), 124.0(^{3}JCF 8 Hz), 123.1, 118.0(^{2}JCF 23 Hz), 117.8(^{2}JCF 23 Hz), 114.7, 109.8, 52.8, 51.7, 36.1, 31.4 IR (neat): 2966, 1666, 1518, 1184, 1129 HRMS ESI m/z: Found 510.2784 $[M+H]^+$ Calculated 510.2787.

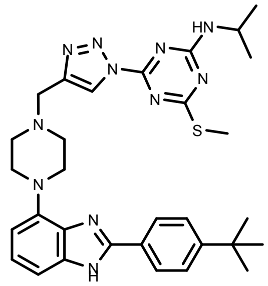

and

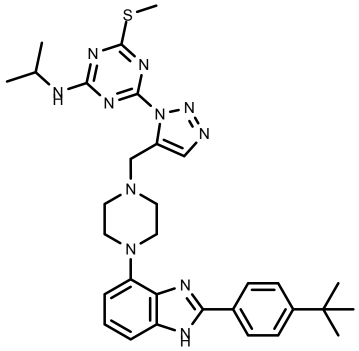

Regioisomeric mixture of 4-(4-((4-(2-(4-*tert*-butylphenyl)-1H-benzo[d]imidazol-4-yl)piperazin-1-yl)methyl)-1H-1,2,3-triazol-1-yl)-N-isopropyl-6-(methylthio)-1,3,5-triazin-2-amine

(8j) and 4-(5-((4-(2-(4-*tert*-butylphenyl)-1H-benzo[d]imidazol-4-yl)piperazin-1-yl)methyl)-1H-1,2,3-triazol-1-yl)-N-isopropyl-6-(methylthio)-1,3,5-triazin-2-amine (8j)
60 mg (1.0 equiv., 0,2 mmol) of 2-(4-(*tert*-butyl)phenyl)-4-(4-(prop-2-yn-1-yl)piperazin-1-yl)-1H-benzo[d]imidazole **(7b)** added to a solution of 6 mg of Cu(II)sulphate pentahydrate (0,2 equiv.) and 18 mg of sodium ascorbate (0,4 equiv.) in 8,75 ml 1:1 destilled H_2O and *tert*-butanol. 36 mg (1.0 equiv., 0,2 mmol) of Aziprotryne ® added. The reaction was done in a microwave for 2 hours at 100°C. The solvents were then evaporated, dissolved in 2 ml 1:1 methanol:DMSO and purified by HPLC (Method D). The product was lyophilized to obtain a white powder. Regioisomeric triazole ratio 1:1.3. (19 mg, 20% yield). Purity: 95.1 % ^{1}H-NMR (400MHz Methanol-d_4), δ in ppm = 9.02(s, 0.43H), 8.99(s, 0.57H), 8.05 (d, J = 8.1 Hz, 2H), 7.71 (d, J = 8.2 Hz, 2H), 7.45 (d, J = 4.2 Hz, 2H), 7.14 (t, J = 2.1 Hz, 1H)., 4.73(s, 2H), 4.30(m, 1H), 3.68(br s, 8H), 2.56(s, 1,5H), 2.53(s, 1.5H), 1.37(s, 9H), 1.25(m, 6H) ^{13}C NMR (100 MHz, Methanol-d_4) δ in ppm = 185.4, 184.2, 165.6, 165.0, 162.9, 162.6, 160.4, 160.1, 158.3, 158.3, 151.6, 140.4, 138.2, 135.3, 135.2, 129.1, 127.8, 127.8, 127.7, 127.2, 127.1, 122.7, 115.1, 109.9, 52.9, 51.5, 44.5, 44.2, 36.2, 31.4, 22.5, 22.3, 13.6, 13.6 IR (neat): 2971, 1667, 1582, 1191, 1129 HRMS ESI m/z: Found 598.3185 $[M+H]^+$ Calculated 598.3189.

Protocol for stimulating Gonadotropin-releasing hormone receptor (GnRHR) using luteinizing hormone releasing hormone

Catalog Number: DC1283
Lot Number: DC1283-092215
Quantity: 1 vial (4 x 106) frozen cells
Freeze Medium: Sigma FreezingMedium (C-6164)
Host cell: HEK293T
Transfection: Expression vector containing full-length human GNRHR cDNA (GenBank Accession Number NM_000406) with FLAG tag sequence at N-terminus
Recommended Storage: Liquid nitrogen upon receiving
Propagation Medium: DMEM + 10% FBS
Stability: 1 – 2 days after thawing

Seed directly into assay microplates after thawing cells and run assays immediately on the same day.

Run a control experiment side-by-side with the division-arrested cells to ensure your assay system is working, such as isproterenol for cAMP assay and ionomycin for Ca++ assay on parental cells.

HEK-293T, 1321N1	Ca++	cAMP (Gαi-coupled)	cAMP (Gαs-coupled)
DMEM (Corning Cellgro 10- 013-CV): 90%; _FBS (Seradigm Catalog #1500-500): 10%	Hanks' Balanced Salt Solution (HBSS) with 20 mM HEPES, pH 7.4	PBS +/- IBMX (TBD by titration)	1 mM IBMX in PBS
Plating cell density for 384- well microplate for Ca++ assay immediately<br><br>5-15,000 cells/well		Plating cell density for 384- well microplate for cAMP assay immediately<br><br>3,000 -12,000 cells/well	Forskolin concentration for cAMP assay (Gαi-coupled)<br>5-15 µM without IBMX; or<br>1-5 µM with 1 mM IBMX

Assay preparations, pretreatment of cells and seeding on 384 well plates.

Thawing Medium was prepared by mixing commercially available stock solutions of DMEM (Corning Cellgro 10-013-CV) and Fetal Bovine Serum, FBS (Seradigm Catalog #1500-500) in a volume ratio of 9:1.

Vials containing 4 x 10^6 frozen cells were transferred from liquid nitrogen to a 37ºC water bath and gently warmed while submerged under water. Immediately after the frozen cells are thawed, the cryovial was removed from the water bath, dried and cleaned with 70% alcohol. Using a 2 mL serological pipette, the cell suspension was transferred to a 15-mL conical centrifuge tube containing 5 mL DMEM pre-warmed to r.t.. The mixture was triturated gently with the same 2 mL serological pipette without causing any air bubbles.
The 15-mL tube was spun at 200x g for 5 minutes at r.t. the supernatant was removed and cells were resuspended in 11 mL of DMEM complete to obtain a cell concentration of 360000 cells/ml. Based on previous titration of cell densitites, 9000 cells were seeded per well and the plate was incubated over night at 37 °C/CO_2. The next day cells are nicely attached and stretched reaching ~90-100 % confluency.

Buffer preparation:
1. Loading buffer from MD is prepared. Dissolve 1 vial of dye in 10 mL buffer from kit.
2. HBBS/Hepes (1x with Ca/Mg)/20 mM Hepes, pH 7.4 with 0.1 % BSA to be used for dilution of the agonist peptide
3. 60 mL HBBS/20 mM Hepes to be added to test compounds

Preparing the cells
The day of the assay the seeding medium was carefully decanted from each well by tilting the plate. 25 µl loading buffer containing the Ca^{2+} sensing dye was added to each well. Subsequently, 20 µL of HBBS/Hepes were added to each well containing dye. Plates were incubated at 37 °C for 45 min, after which 5 µL antagonist solution (diluted in HBBS/HEPES) were dispensed into each well using a Hamilton 384 well head followed by incubation at r.t. for 15 min. After 15 min agonist was added on the FLIPR platform and the fluorescence signal was recorded.

Run FLIPR method:
Settings:
First sequence: 2 s, 30 sample. (Baseline)
Second sequence: 2 s, 120 sample. (Agonist stimulation)
12.5 µL addiiton, after sample 30.
Addition height: 35
Addition speed: 20 µl/sec
Compound concentration: 5x, add 12.5 µl of each
One minute baseline stabilisation, 240 s measurement

Compound plate preparation:
Agonist stock solution: Luteinizing hormone releasing hormone, 10 mM in water
Mw: LHRH (GnRH) 1183 g/mol.
Dissolve 5 mg in 423 µL of water, store at -80 °C until use.
Reported EC50 = 5.1 x10^{-9} M to 7.8x10^{-8} M,

Prepare a full plate with only agonist at experimental EC50 dose as reported before on FLIPR platform:
EC50 = 258 nM. 5x stock = 258 nM x 5 = 1290 nM.
Dilute 3,225 µL in 25 mL HBSS/Hepes buffer with 0.1 % BSA

Test compounds (antagonists)
Sample to be analysed (>95% pure by HPLC/microanalysis, fully characterised).
DMSO for cell culture (reference free, anhydrous) stored in desiccator 1: Dimethyl sulfoxide (Hybri-Max™, sterile-filtered, BioReagent, suitable for hybridoma, ≥99.7%, D2650-5X10ML, Sigma-Aldrich)
Sample preparation:
A - Solubility of the sample is assessed with an aliquot of the material and DMSO prior to sample preparation.
B - 10 ±1 mg of each neat, homogeneous sample are weighed into a labelled 1.5 mL flip-top Eppendorff tube.
C – The amount of DMSO required for a 10 mM stock concentration is calculated using Formula (I)

$$(1)\ V_{DMSO} = \frac{m(test\,compound)}{M(test\,compound)} \times 1000\mu L\, mol^{-1}$$

D – This exact volume in microliters is added to the vial, the vial is closed and vortexed for 15 seconds in triplicate.
F – The vial is inspected visually. (Clouding, floating solids or phase separation render the sample useless.)
G - All vials are stored in a labelled sample rack and frozen at -20 °C until plating.

Stock concentration is 10mM.
Using an Echo nanovolume acoustic dispenser, eleven dilutions of each test compound are prepared on dry plates plates by dispensing the appropriate volume of a 10 mM stock solution of each compound in 100 % DMSO for cell culture, followed by 50 µL of assay buffer into each well to obtain 10x concentrated stock of each test compound. Add 50 µL buffer to wells
Aliquot of 5 uL of the obtained dilutions are added directly to cells using a Hamilton robot and preincubated 15 min before agonist are added to each well using FLIPR to obtain the final concentrations depicted in Table s1:

**Stock:**	**10 mM**	**Echo prepared curves**	V_{total} **= 62,5 uL**		**final dmso (%) in well**
**dilution**	**c in nM**	**c in M**	**c in nM final**	**LOG(c), [c] = M**	
1:200	50000	0,0005	4000	-4,301029996	0,5
1:600	16667	0,0001665	1333,333333	-4,77815125	0,1665
1:2000	5000	0,0000555	400	-5,301029996	0,055
1:6000	1667	0,0000185	133,3333333	-5,77815125	0,018
1:20000	500	0,000006	40	-6,301029996	0,006
1:60000	167	0,000002	13,33333333	-6,77815125	0,002
1:200000	50	0,0000005	4	-7,301029996	0,0005
1:600000	17	2,275E-07	1,333333333	-7,77815125	0,02
1:2000000	5	7,39375E-08	0,4	-8,301029996	0,0065
1:6000000	1,7	2,275E-08	0,133333333	-8,77815125	0,002
1:20000000	0,50	5,6875E-09	0,04	-9,301029996	0,00065

Readout signal test:
LOW: Cells, no agonist, only add buffer using FLIPR
HIGH: Cells, agonist stimulation
Compound doses: 11 point dose, 15 compounds + 1 DMSO series total of 352 wells per plate.

Thermodynamic solubility assay

Analysis performed at Analiza, Cleveland Ohio.

Apparatus Agilent G6538 QTOF Mass Spectrometer equipped with dual Electrospray Ionization with Mass Hunter data software package Agilent 1290 Infinity II UHPLC system equipped with photodiode array detector, binary pump, vacuum degasser, column heater, thermostat and validated data acquisition software Standard Laboratory equipment (e.g. Pyrex volumetric flasks, graduated cylinders and bottles, sonicator, vortexer, digital timer, calibrated analytical balance, pipettes and tips

Test Compounds: were supplied as dry powder in vials
Consumables: Sodium Chloride (Cat # 21618) was purchased from Affymetrix, Cleveland, OH. HPLC grade water (Cat # EM-WX0004-1), Omnisolv Acetonitrile and (Cat #EM-AX0142-1, DimethylSulfoxide (Cat #D1258) was purchased from Spectrum Chemical, New Brunswick, NJ. DeepWell 96 well plates (Cat #AWLS-219002) were purchased from Arctic White, Bethlehem, PA. Whatman Mini UniPrep syringe-less filter device with 0.45µM PTFE filter membrane (Cat #US203NPUORG), Formic Acid (Cat# 0961-100mL) and Round bottom 96 well plates (Cat# 29444-104) were purchased from VWR, Radnor, PA. Benzthiazide (Cat # B7149) and Alprenolol (Cat# A0360000) were purchased from Sigma-Aldrich, St. Louis, MO. Hydrochloric acid,36%, (Cat # 124635000) and Phosphate Buffered Saline, 10X PBS solution, (Cat # BP399-500) were purchased from Fisher Scientific, Fair Lawn, New Jersey.

Preparation of Calibration Standards for LC-MS Analysis:
Separate test compound was weighed into 4mL vials for calibration curves. Samples were received as dry powders in vials containing approximately 5mg of material each. Upon arrival at Analiza the vials were intact. DimethylSulfoxide (DMSO) was added to each sample such that the final concentration was 10mM and vortexed for 5-10 seconds. After vortexing, all compounds appeared to be fully dissolved. Each test compound was transferred to a polypropylene 96 well plate for robotic preparation of calibration standards.

Each test article DMSO stock solution was serially diluted using a Hamilton STARlet liquid handler with 50-50 LC-MS water 0.1% Formic Acid and LC-MS acetonitrile 0.1% Formic Acid to final concentrations as described below in Table 1 and plated in a polypropylene 96 well plate sealed with foil seal for analysis. A standard calibration curve was prepared for each compound from these dilutions.

Ambient Temperature Test compounds were received as dry powders in vials containing approximately 5mg of material each. Upon arrival at Analiza the vials were intact. Each sample was weighed into a Whatman vial and the appropriate Buffer (450uL) was added to each vial for a dose concentration of approximately 2mg/mL . Following 24 hour incubation on a rotary shaker (200 RPM) at ambient temperature (22.6-25.0°C), the samples were filtered in a 96 well polypropylene plate. The plate was sealed with a pierceable heat seal and analyzed by LC-QTOFMS.

Quantitation: 1.) LC-QTOFMS: a. Each sample was weighed into a whatman vial and the appropriate buffer (450uL) was added to each vial for a dose concentration of approximately 2mg/mL incubated for 24 hours and filtered b. The filtrates were assayed undiluted or diluted 100X, 1000X, and/or 10000x with a 50:50 solution of mobile phase and eluted using a fast generic gradient program. c. TOFMS Data was acquired using Agilent 6538 Ultra High Accuracy TOF MS in extended dynamic range (m/z 100-1000) using generic MS conditions. d. A standard calibration curve was prepared for each compound spanning the range of the assay e. Following data acquisition, exact mass extraction and peak integration were performed using MassHunter Software (Agilent Technologies). f. The filtrates were quantified with respect to this calibration curve.

Quality: Two on-board reference standards were prepared, incubated, and assayed in triplicate in each media specified with the University of Tromsø compounds, and all the results were found to be within the acceptable range.

References

1. Pelletier, J. C.; Chengalvala, M.; Cottom, J.; Feingold, I.; Garrick, L.; Green, D.; Hauze, D.; Huselton, C.; Jetter, J.; Kao, W.; Kopf, G. S.; Lundquist, J. T. t.; Mann, C.; Mehlmann, J.; Rogers, J.; Shanno, L.; Wrobel, J., 2-phenyl-4-piperazinylbenzimidazoles: orally active inhibitors of the gonadotropin releasing hormone (GnRH) receptor. *Bioorganic & medicinal chemistry* **2008,** *16* (13), 6617-40.

1-Azido-3-fluoro-2-nitrobenzene (2)

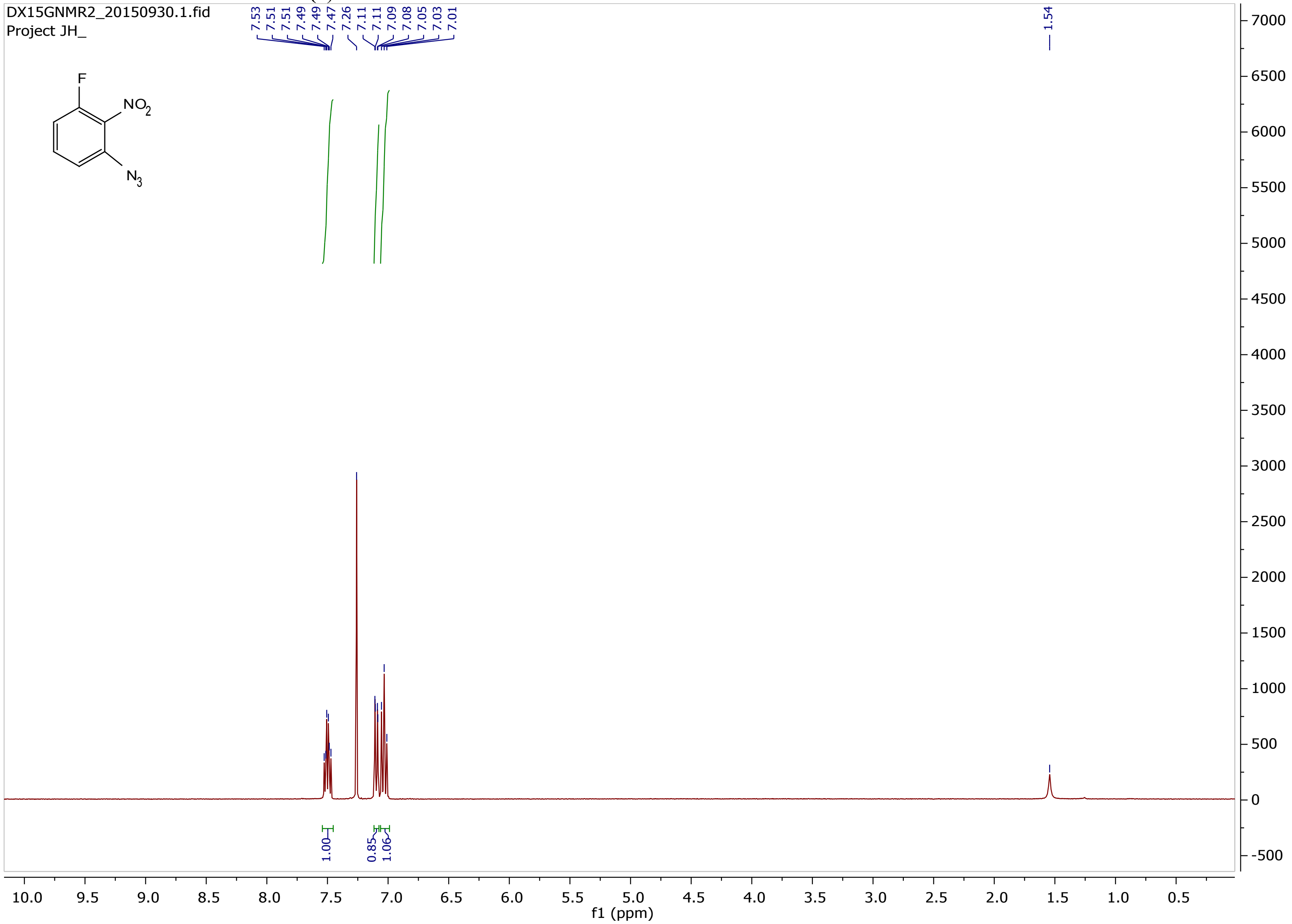

tert-Butyl 4-(3-azido-2-nitrophenyl)piperazine-1-carboxylate

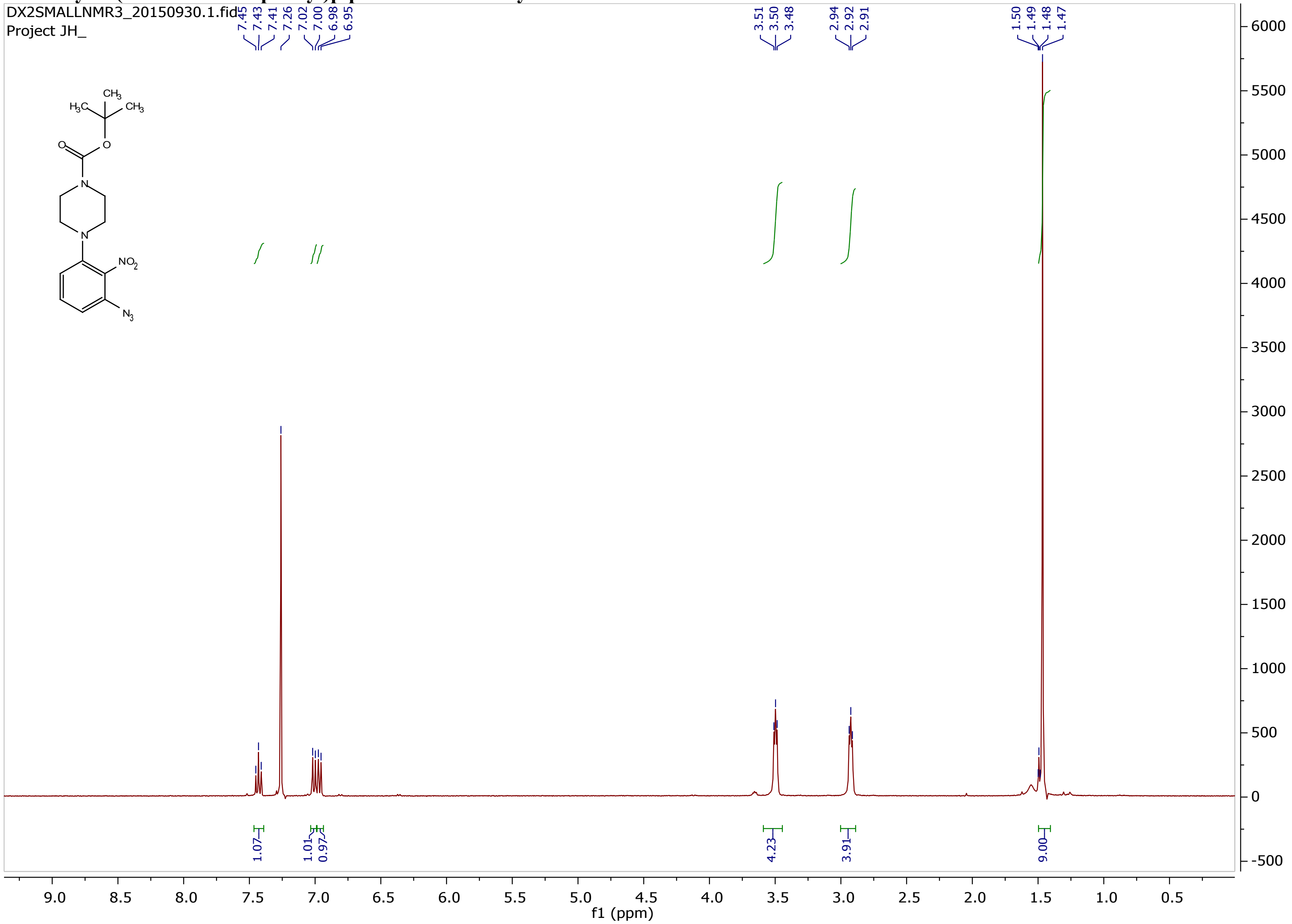

***tert*-Butyl 4-(2,3-diaminophenyl)piperazine-1-carboxylate (4)**

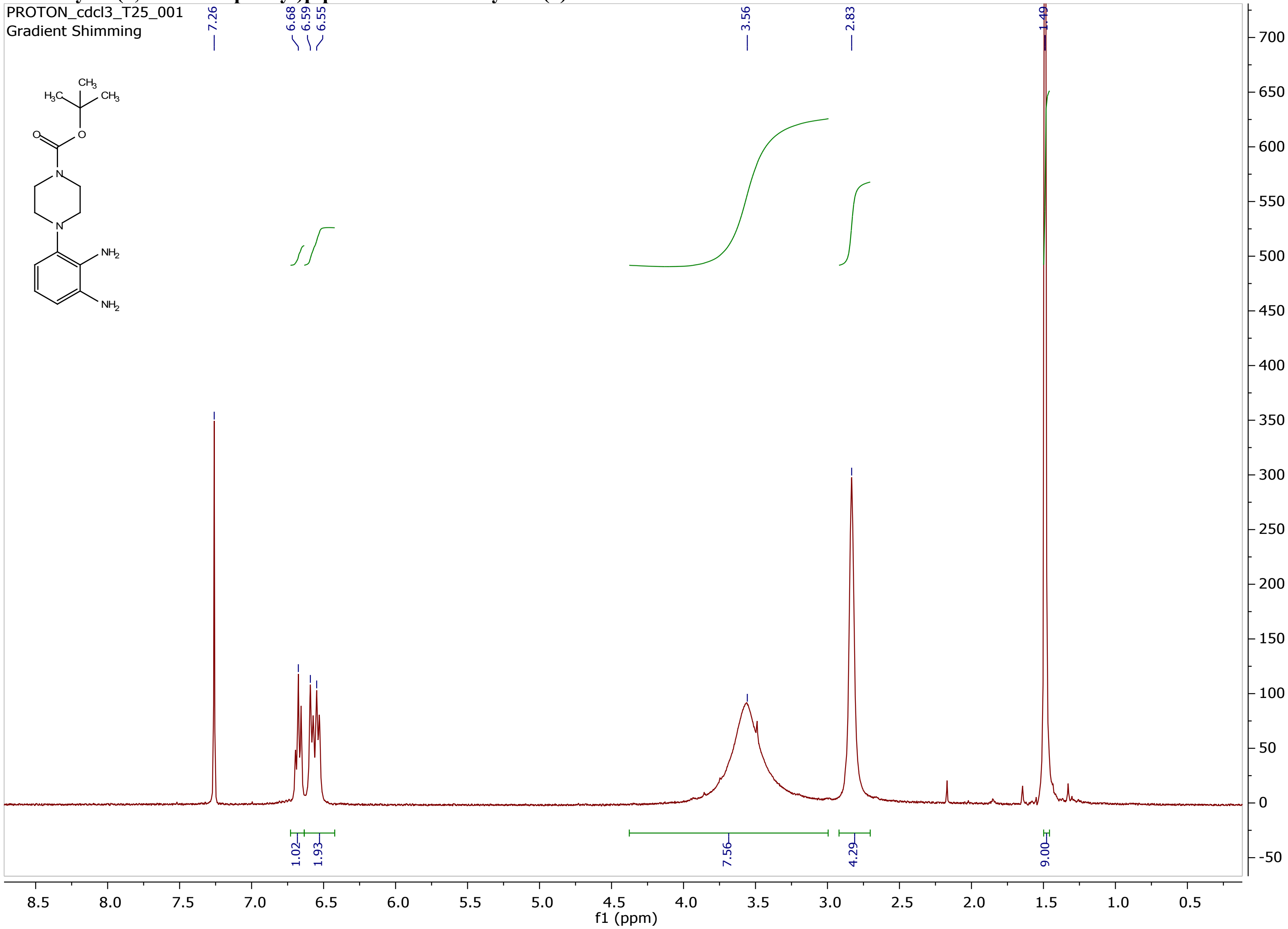

***tert*-Butyl 4-(2-(4-*tert*-butylphenyl)-1H-benzo[d]imidazol(-4-yl)piperazine-1-carboxylate**

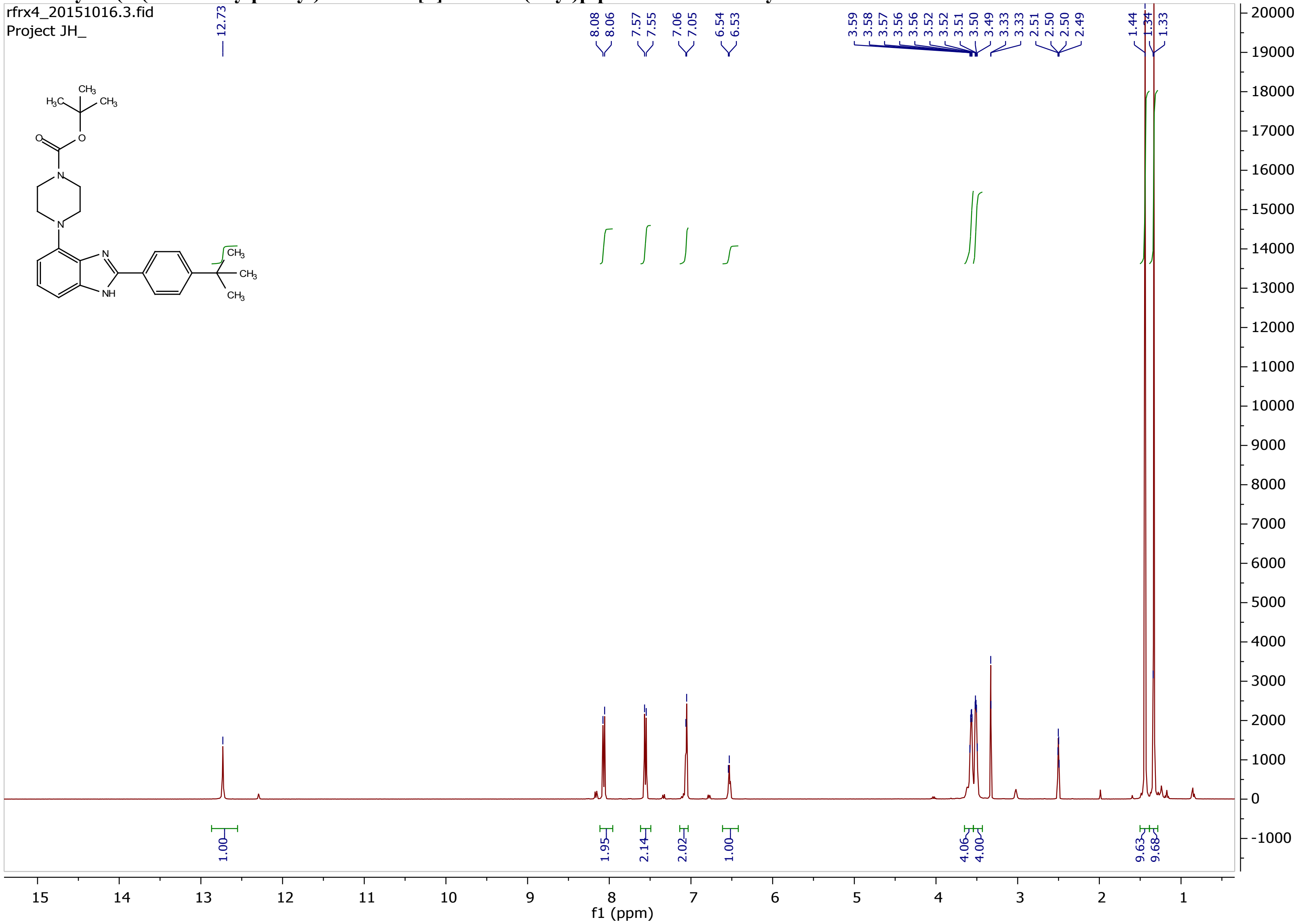

2-(4-*tert*-Butylphenyl)-4-(piperazin-1-yl)-1H-benzo[d]imidazole (6a)

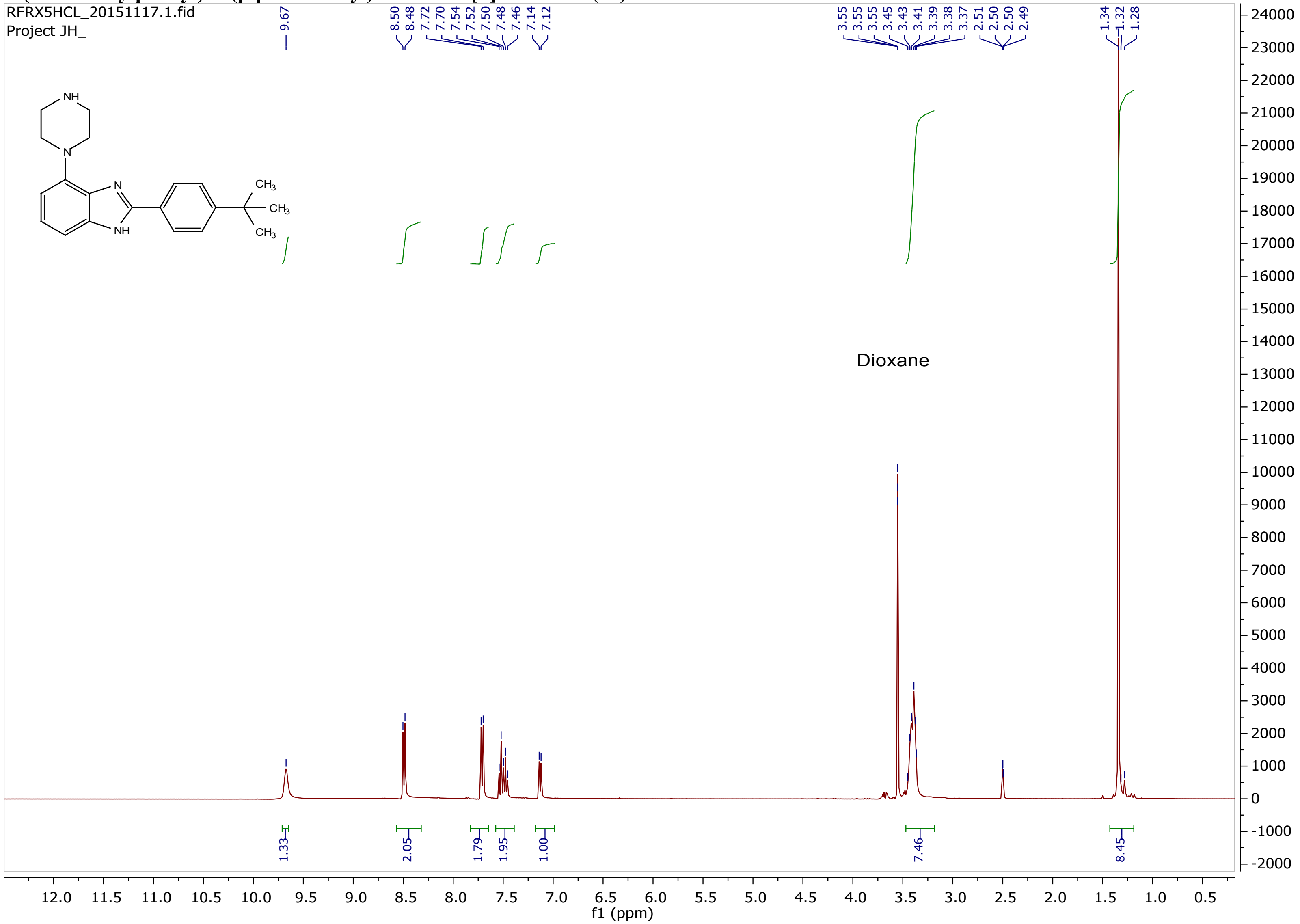

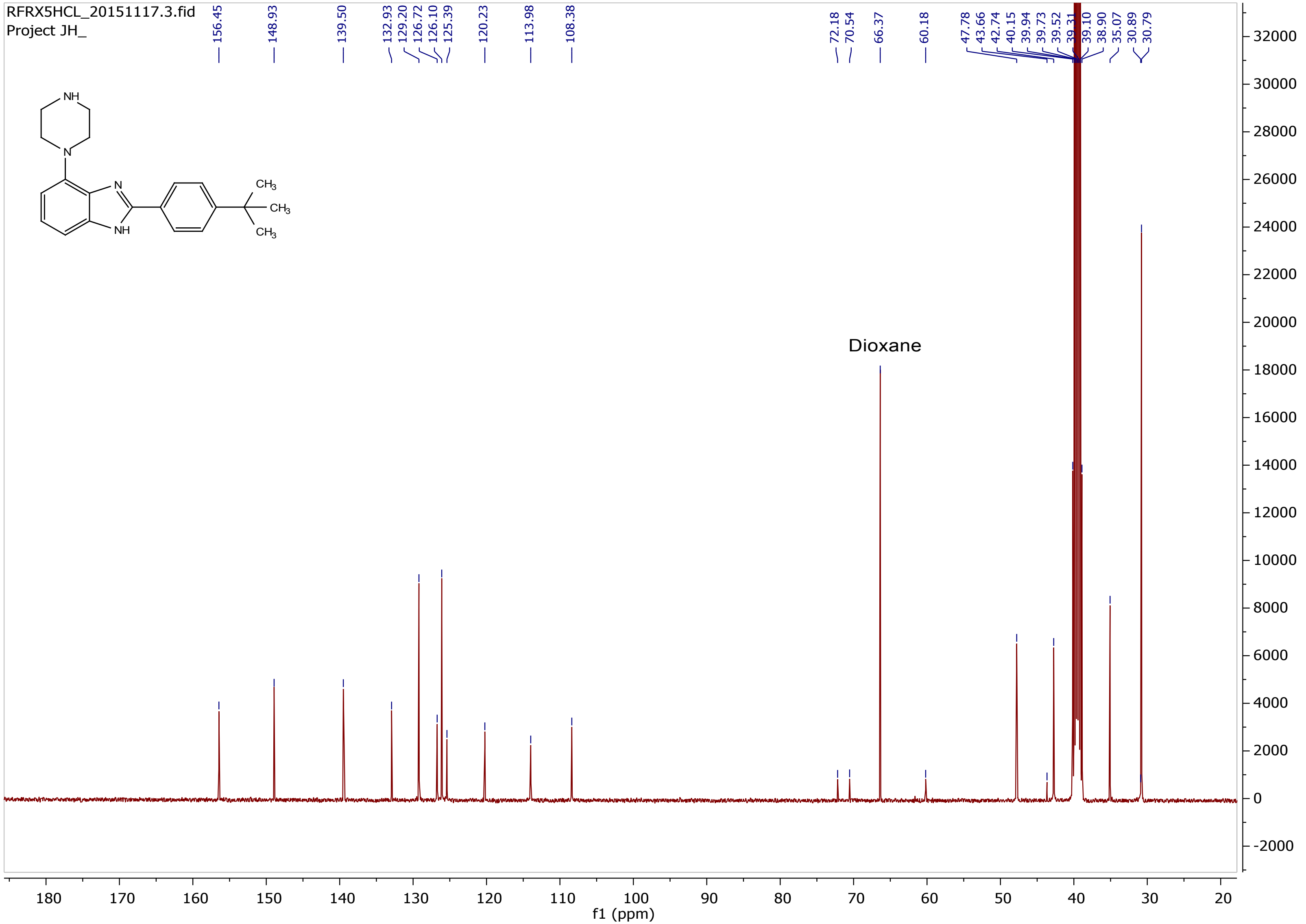

RFRX5HCL_20151117.3.fid
Project JH_
156.45
148.93
139.50
132.93
129.20
126.72
126.10
125.39
120.23
113.98
108.38
72.18
70.54
66.37
60.18
47.78
43.66
42.74
40.15
39.94
39.73
39.52
39.31
39.10
38.90
35.07
30.89
30.79
NH
N
N
NH
CH3
CH3
CH3
Dioxane
f1 (ppm)

1-(4-*tert*-Butylbenzyl)-2-(4-*tert*-butylphenyl)-4-(piperazin-1-yl)-1H-benzo[d]imidazole (6b)

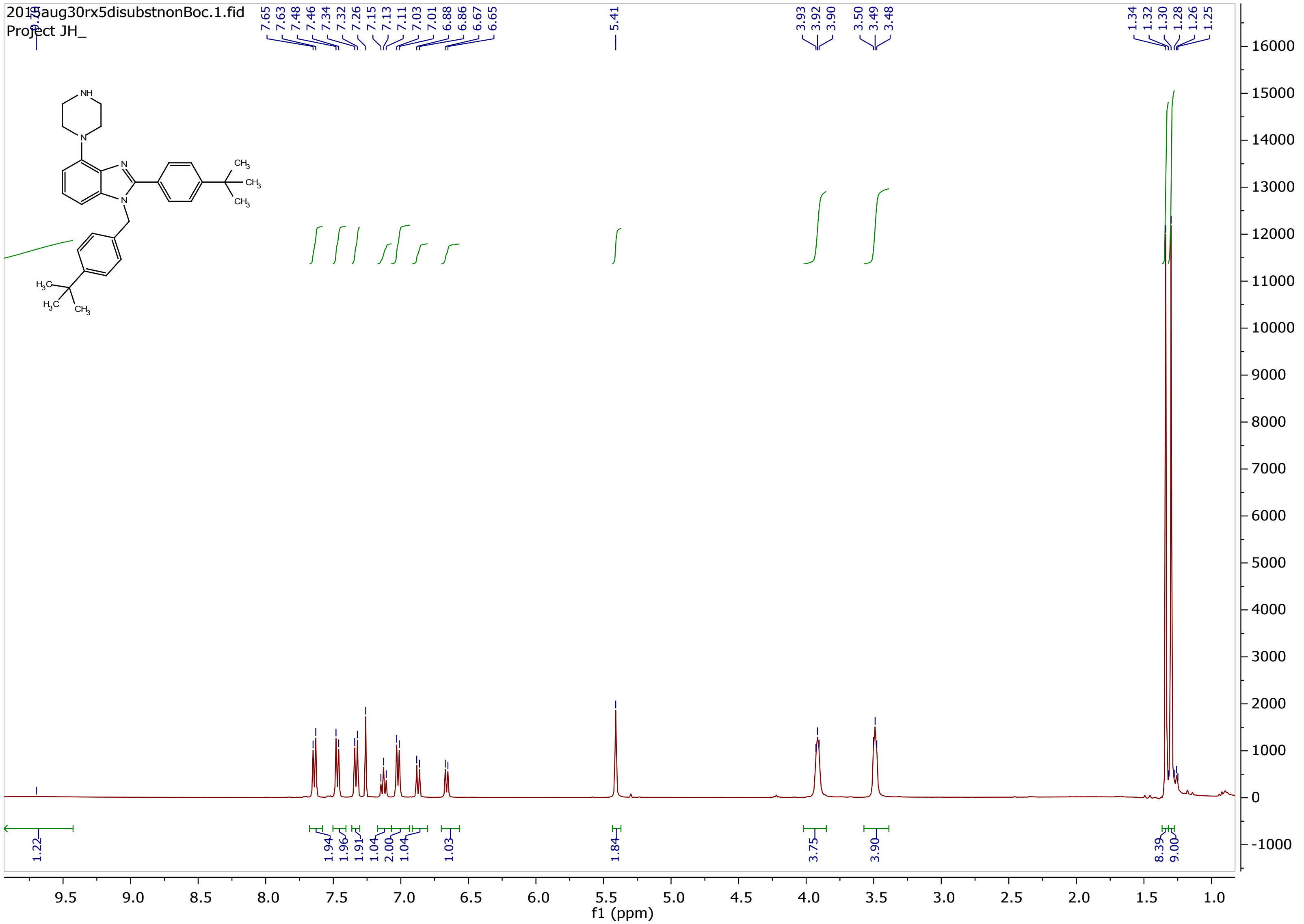

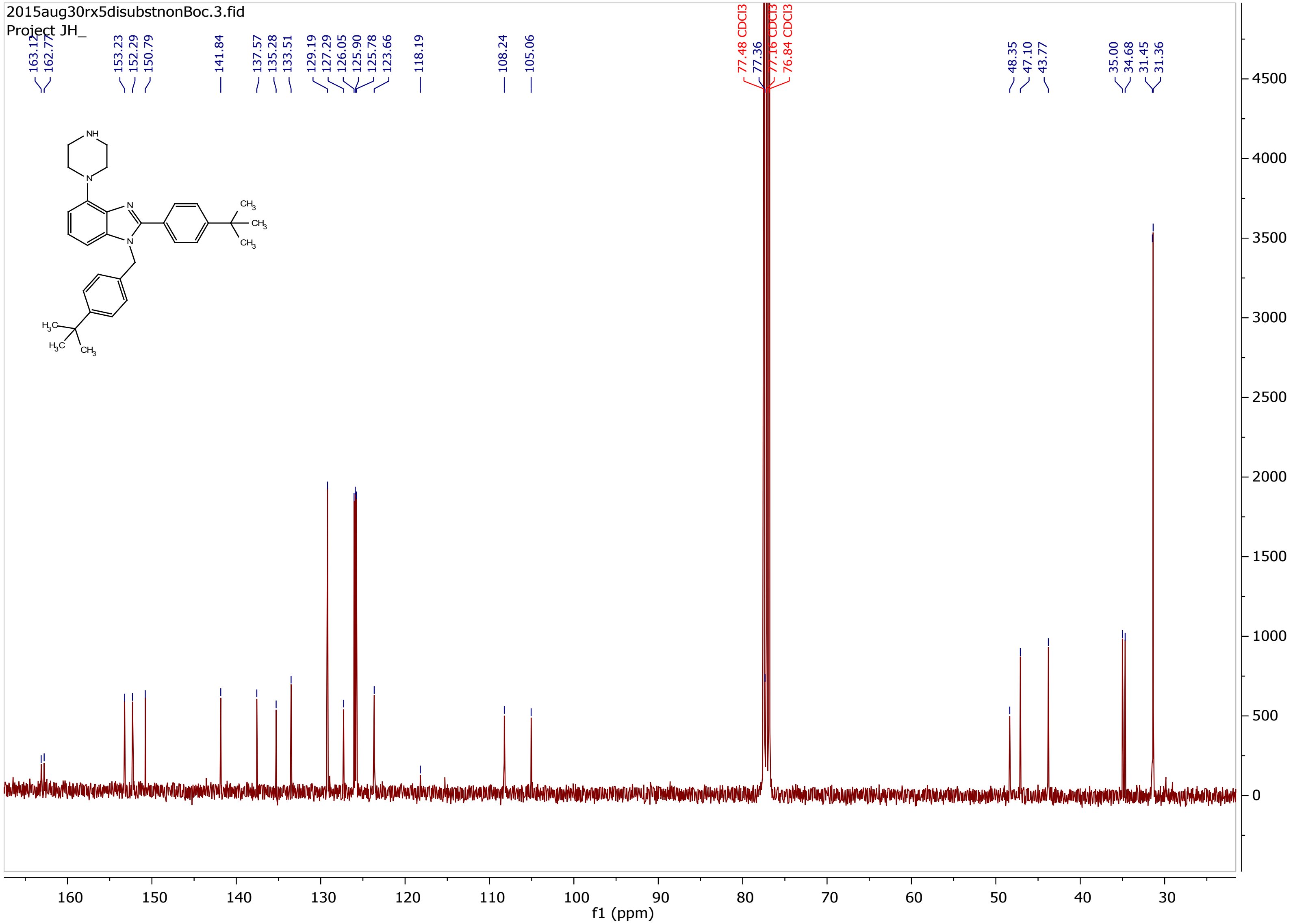
2015aug30rx5disubstnonBoc.3.fid
Project JH_
163.12
162.77
153.23
152.29
150.79
141.84
137.57
135.28
133.51
129.19
127.29
126.05
125.90
125.78
123.66
118.19
108.24
105.06
77.48 CDCl3
77.36
77.16 CDCl3
76.84 CDCl3
48.35
47.10
43.77
35.00
34.68
31.45
31.36
NH
N
N
N
CH3
CH3
CH3
H3C
H3C
CH3
f1 (ppm)

tert-Butyl 4-(1-(4-_tert_-butylbenzyl)-2-(4-_tert_-butylphenyl)-1H-benzo[d]imidazol-4-yl)piperazine-1-carboxylate (5b)

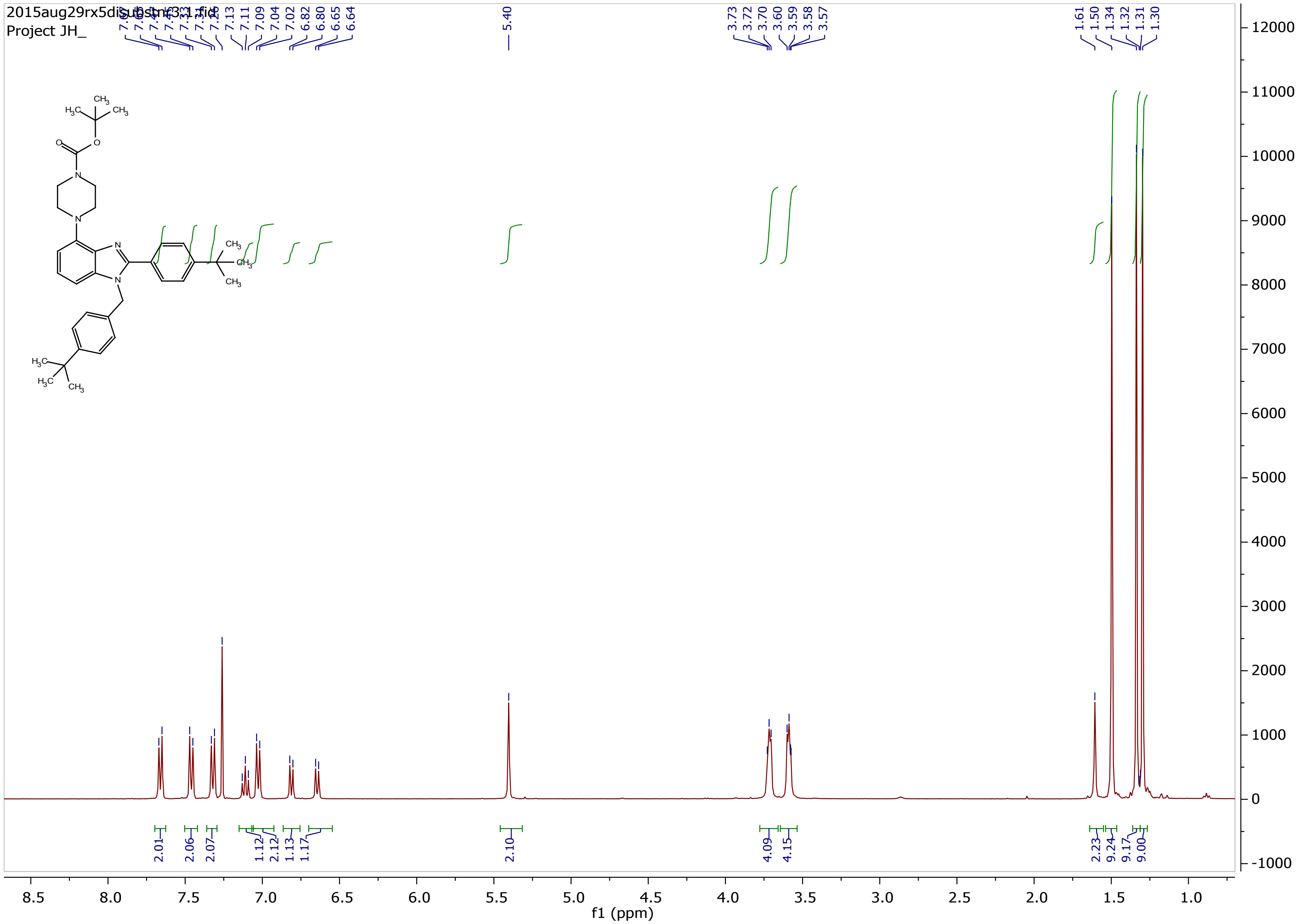

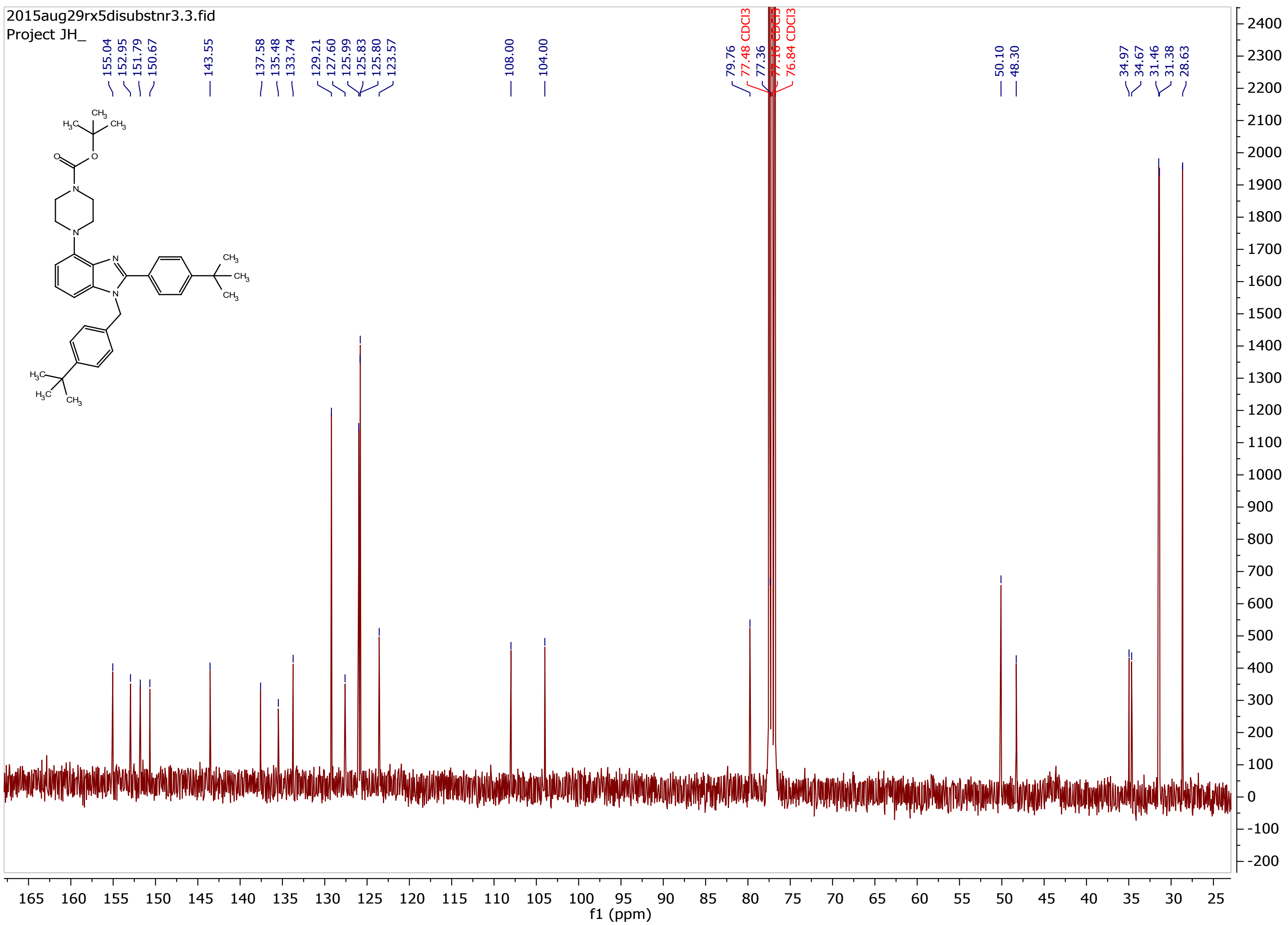
2015aug29rx5disubstnr3.3.fid
Project JH_
155.04
152.95
151.79
150.67
143.55
137.58
135.48
133.74
129.21
127.60
125.99
125.83
125.80
123.57
108.00
104.00
79.76
77.48 CDCl3
77.36
77.16 CDCl3
76.84 CDCl3
50.10
48.30
34.97
34.67
31.46
31.38
28.63
f1 (ppm)

tert-Butyl 4-(2-(biphenyl-4-yl)-1H-benzo[d]imidazol-4-yl)piperazine-1-carboxylate (5c)

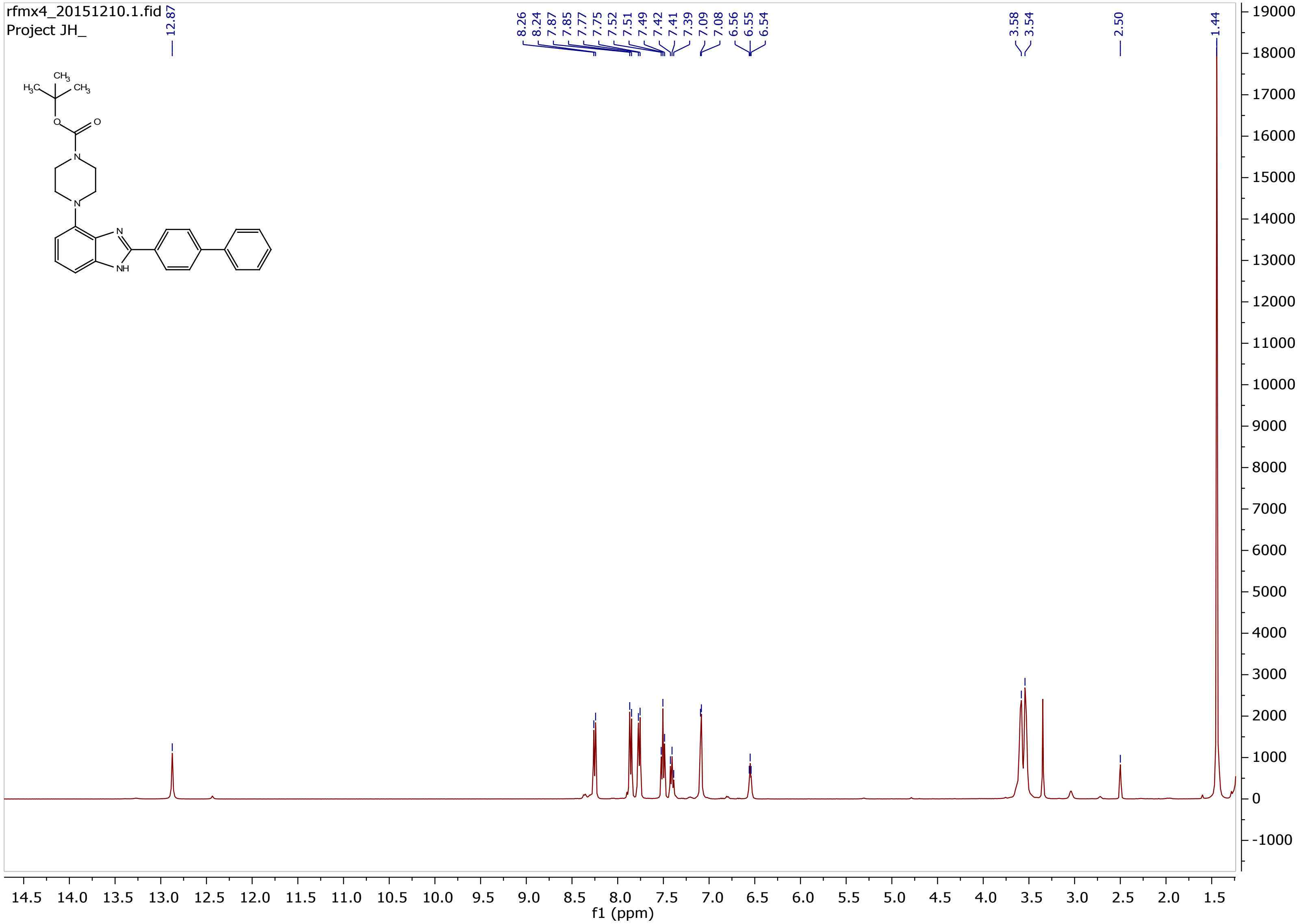

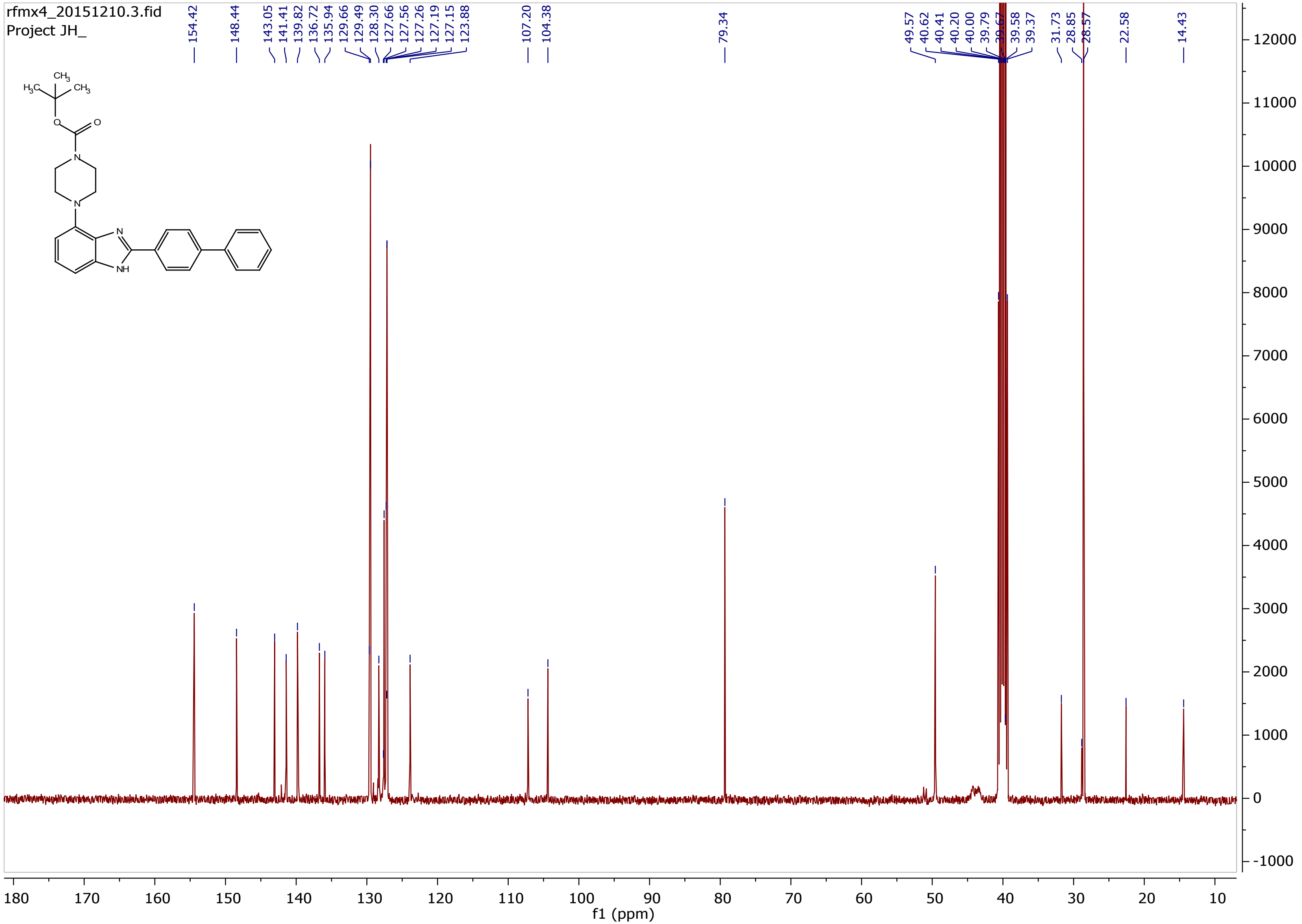
rfmx4_20151210.3.fid
Project JH_
154.42
148.44
143.05
141.41
139.82
136.72
135.94
129.66
129.49
128.30
127.66
127.56
127.26
127.19
127.15
123.88
107.20
104.38
79.34
49.57
40.62
40.41
40.20
40.00
39.79
39.67
39.58
39.37
31.73
28.85
28.57
22.58
14.43
f1 (ppm)

2-(Biphenyl-4-yl)-4-(piperazin-1-yl)-1H-benzo[d]imidazole (6c)

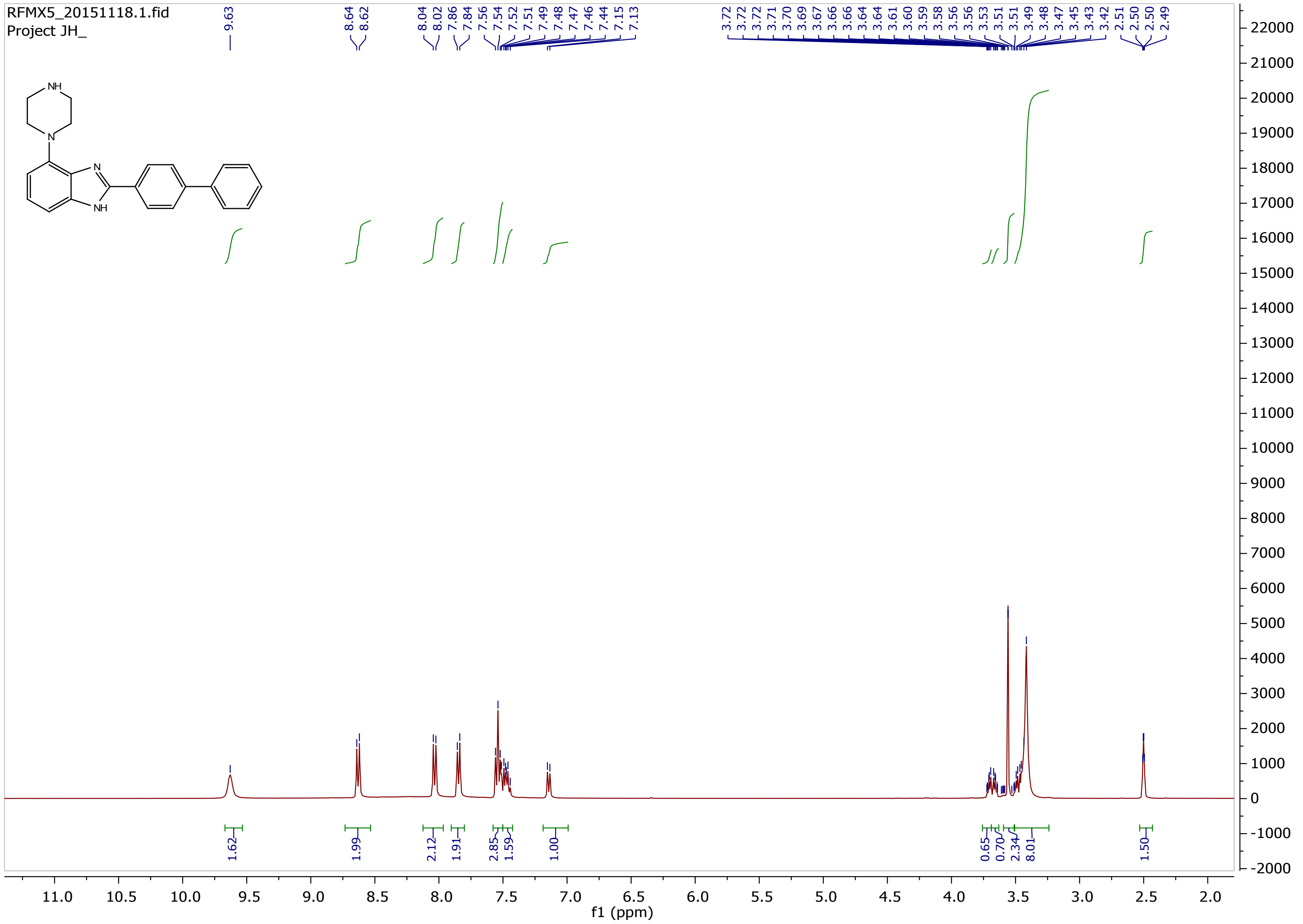

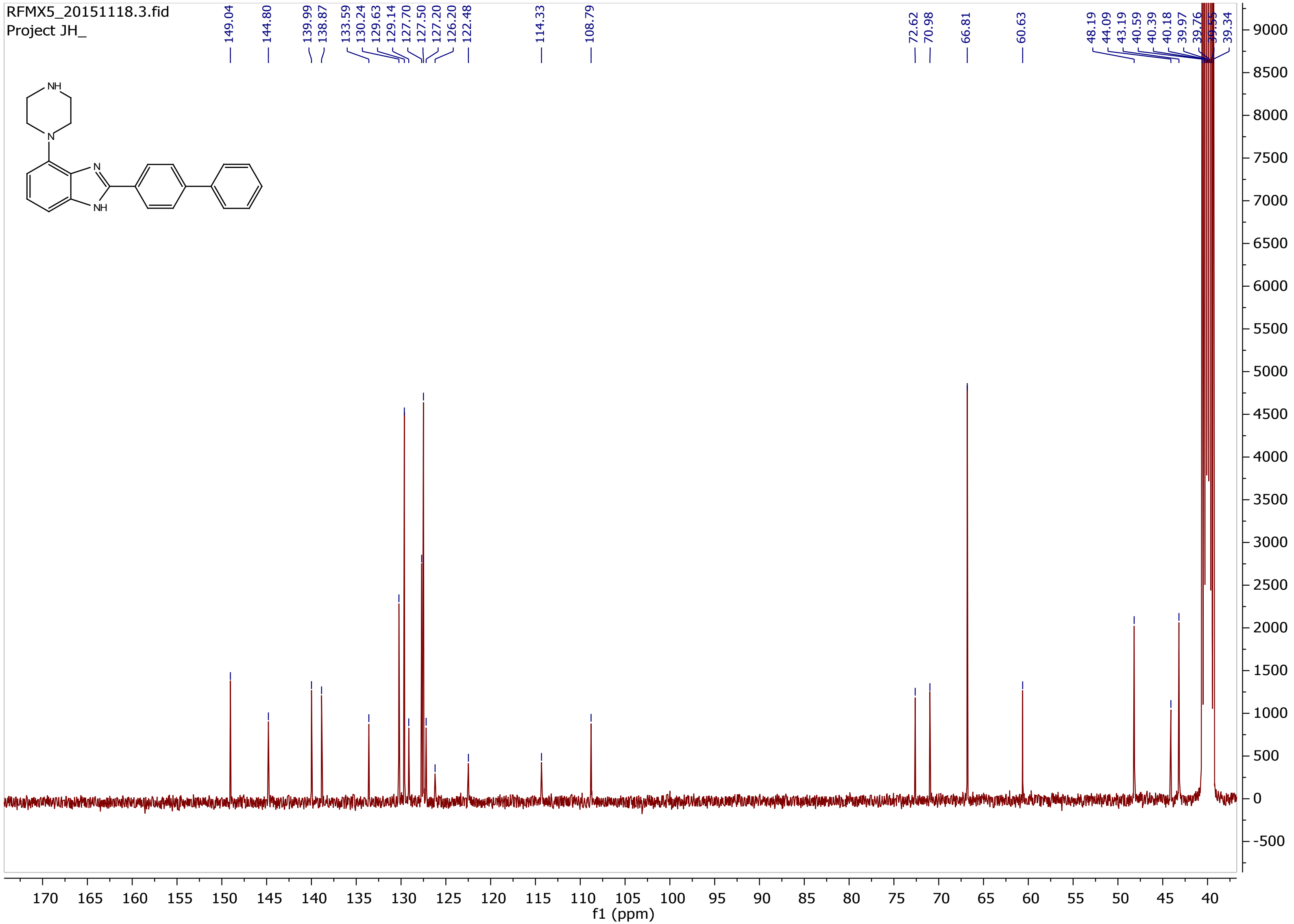
RFMX5_20151118.3.fid
Project JH_
149.04
144.80
139.99
138.87
133.59
130.24
129.63
129.14
127.70
127.50
127.20
126.20
122.48
114.33
108.79
72.62
70.98
66.81
60.63
48.19
44.09
43.19
40.59
40.39
40.18
39.97
39.76
39.34
f1 (ppm)

2-(4-(*tert*-Butylphenyl)-4-(4-(2-fluoroethyl)piperazin-1-yl)-1H-benzo[d]imidazole (7a)

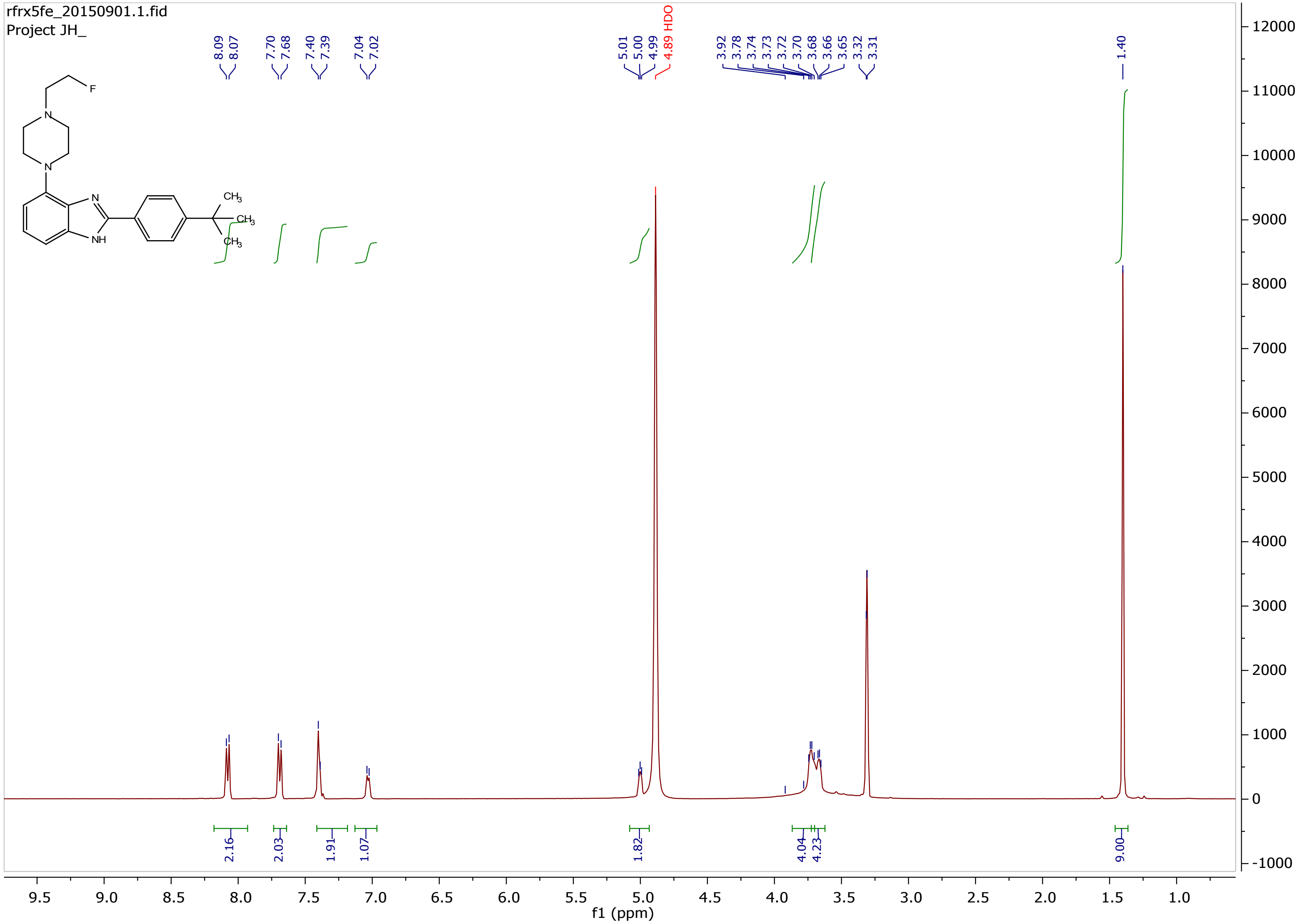

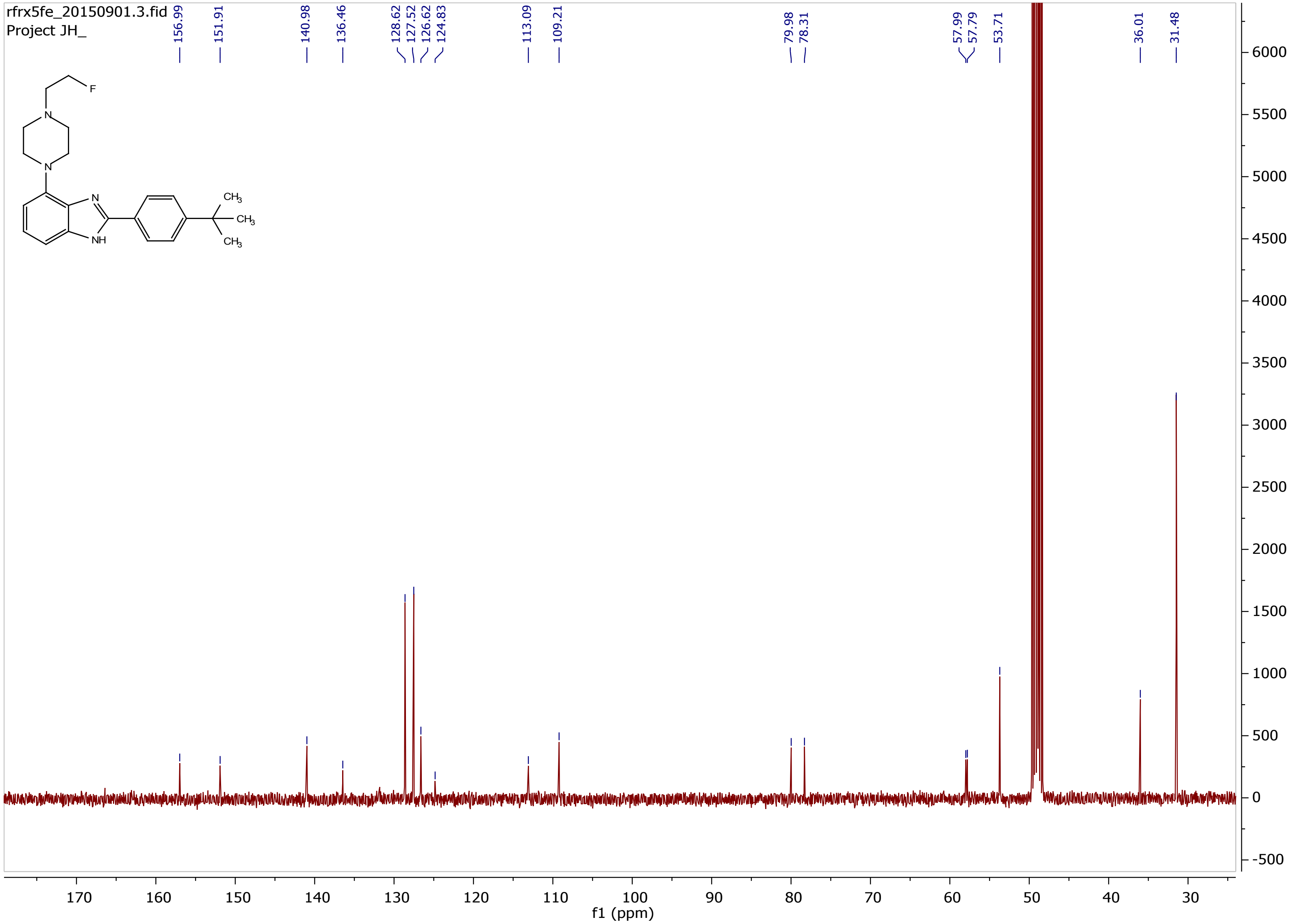
rfrx5fe_20150901.3.fid
Project JH_
156.99
151.91
140.98
136.46
128.62
127.52
126.62
124.83
113.09
109.21
79.98
78.31
57.99
57.79
53.71
36.01
31.48
F
N
N
N
NH
CH3
CH3
CH3
f1 (ppm)

2-(4-(*tert*-Butyl)phenyl)-4-(4-(prop-2-yn-1-yl)piperazin-1-yl)-1H-benzo[d]imidazole (7b)

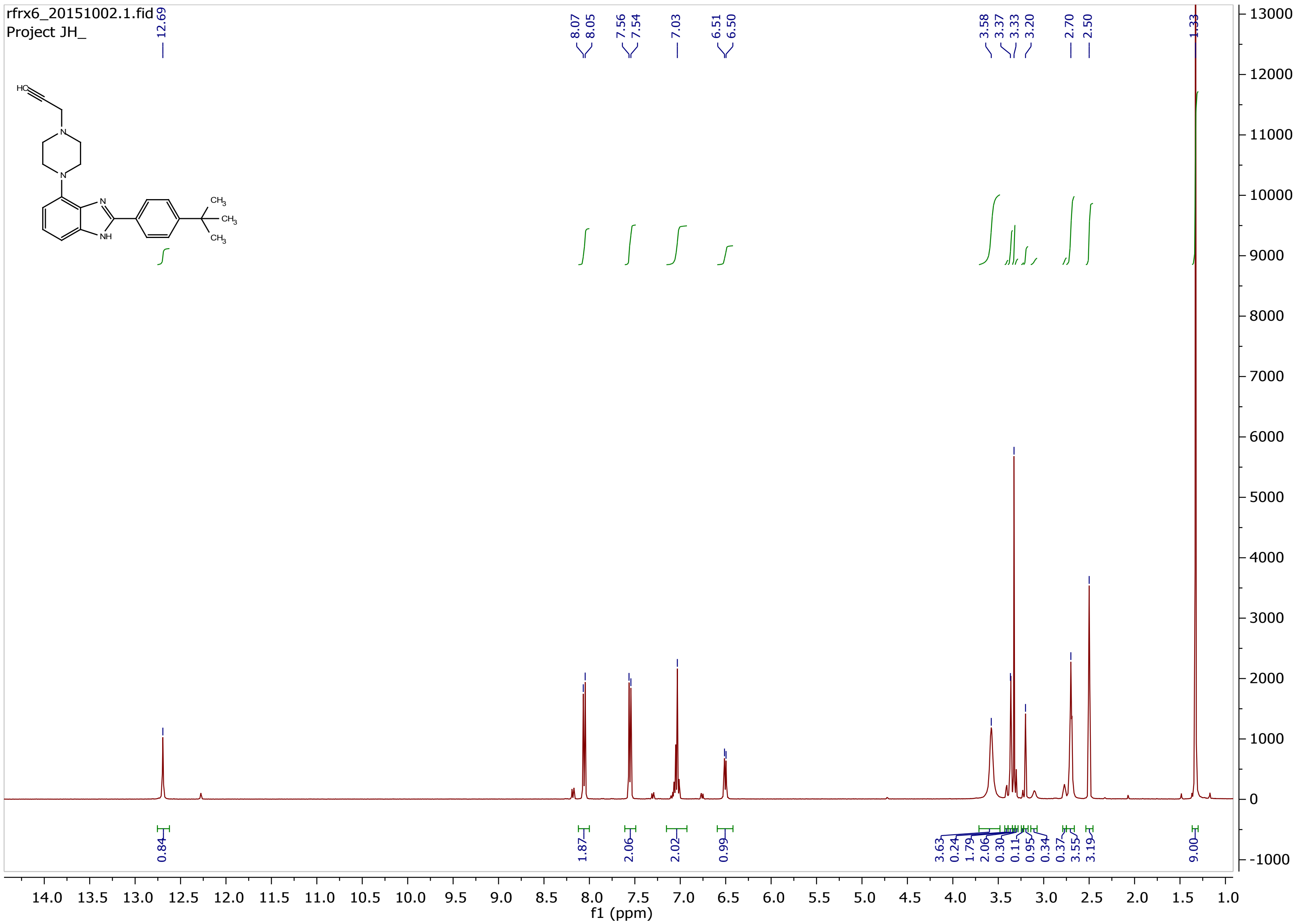

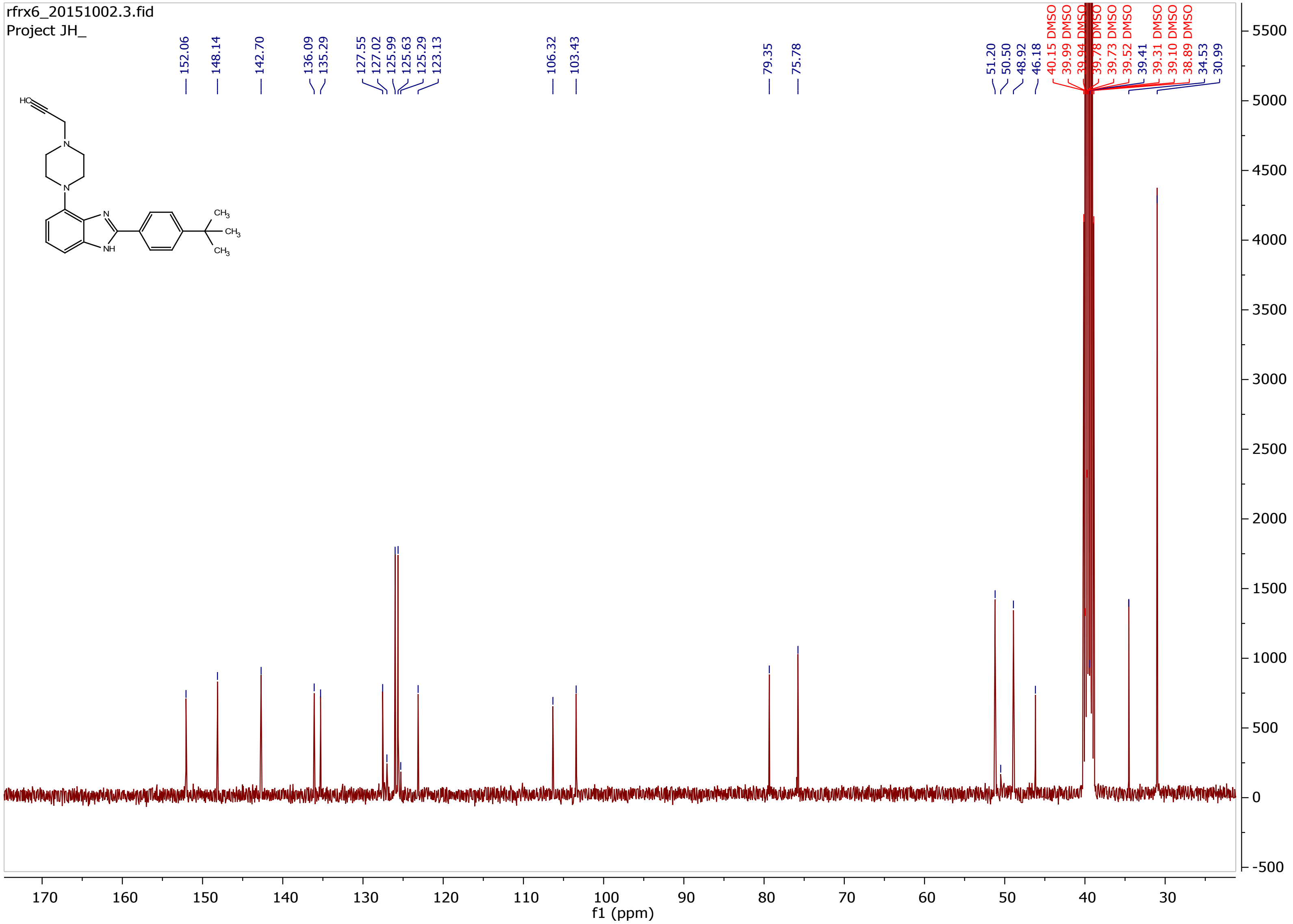
rfrx6_20151002.3.fid
Project JH_
152.06
148.14
142.70
136.09
135.29
127.55
127.02
125.99
125.63
125.29
123.13
106.32
103.43
79.35
75.78
51.20
50.50
48.92
46.18
40.15 DMSO
39.99 DMSO
39.94 DMSO
39.78 DMSO
39.73 DMSO
39.52 DMSO
39.41
39.31 DMSO
39.10 DMSO
38.89 DMSO
34.53
30.99
f1 (ppm)

5-Acetamido-2-(acetoxymethyl)-6-(4-((4-(2-(4-*tert*-butylphenyl)-1H-benzo[d]imidazol-4-yl)piperazin-1-yl)methyl)-1H-1,2,3-triazol-1-yl)tetrahydro-2H-pyran-3,4-diyl diacetate (8c)

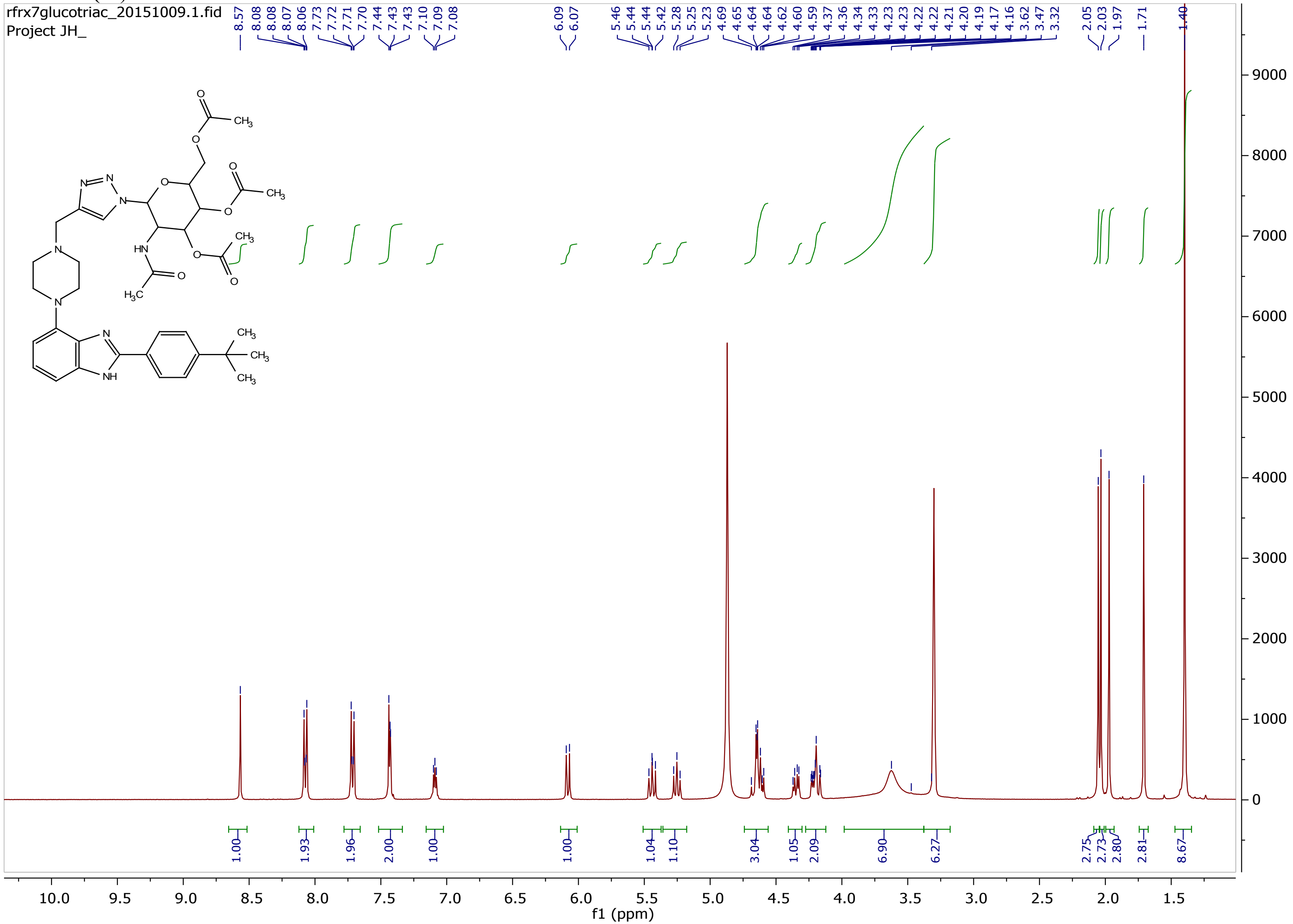

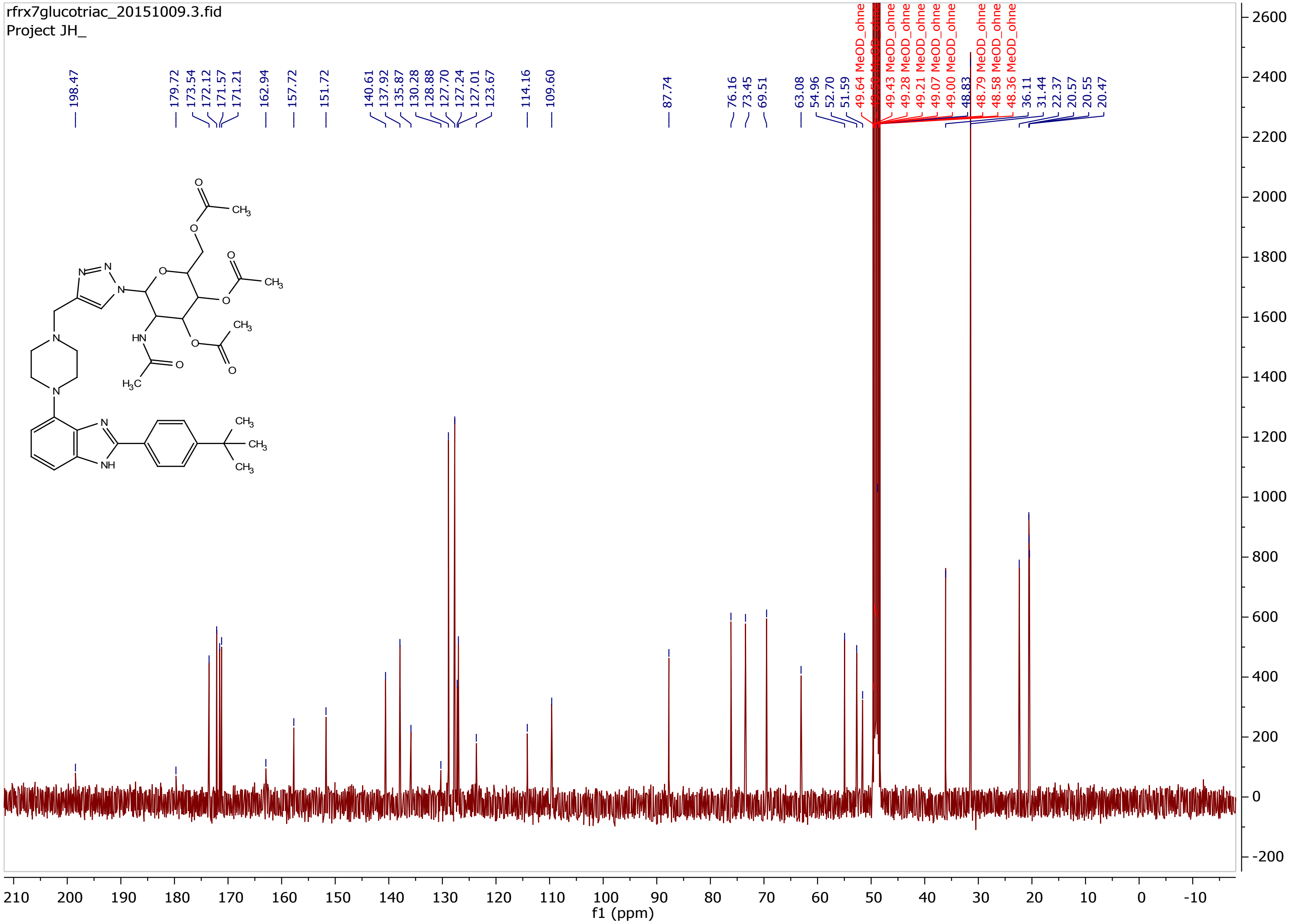
rfrx7glucotriac_20151009.3.fid
Project JH_
198.47
179.72
173.54
172.12
171.57
171.21
162.94
157.72
151.72
140.61
137.92
135.87
130.28
128.88
127.70
127.24
127.01
123.67
114.16
109.60
87.74
76.16
73.45
69.51
63.08
54.96
52.70
51.59
49.64 MeOD_ohne
49.43 MeOD_ohne
49.28 MeOD_ohne
49.21 MeOD_ohne
49.07 MeOD_ohne
49.00 MeOD_ohne
48.83
48.79 MeOD_ohne
48.58 MeOD_ohne
48.36 MeOD_ohne
36.11
31.44
22.37
20.57
20.55
20.47
f1 (ppm)

2-(*tert*-Butoxycarbonylamino)-3-(4-((4-(2-(4-*tert*-butylphenyl)-1H-benzo[d]imidazol-4-yl)piperazin-1-yl)methyl)-1H-1,2,3-triazol-1-yl)propanoic acid (8b)

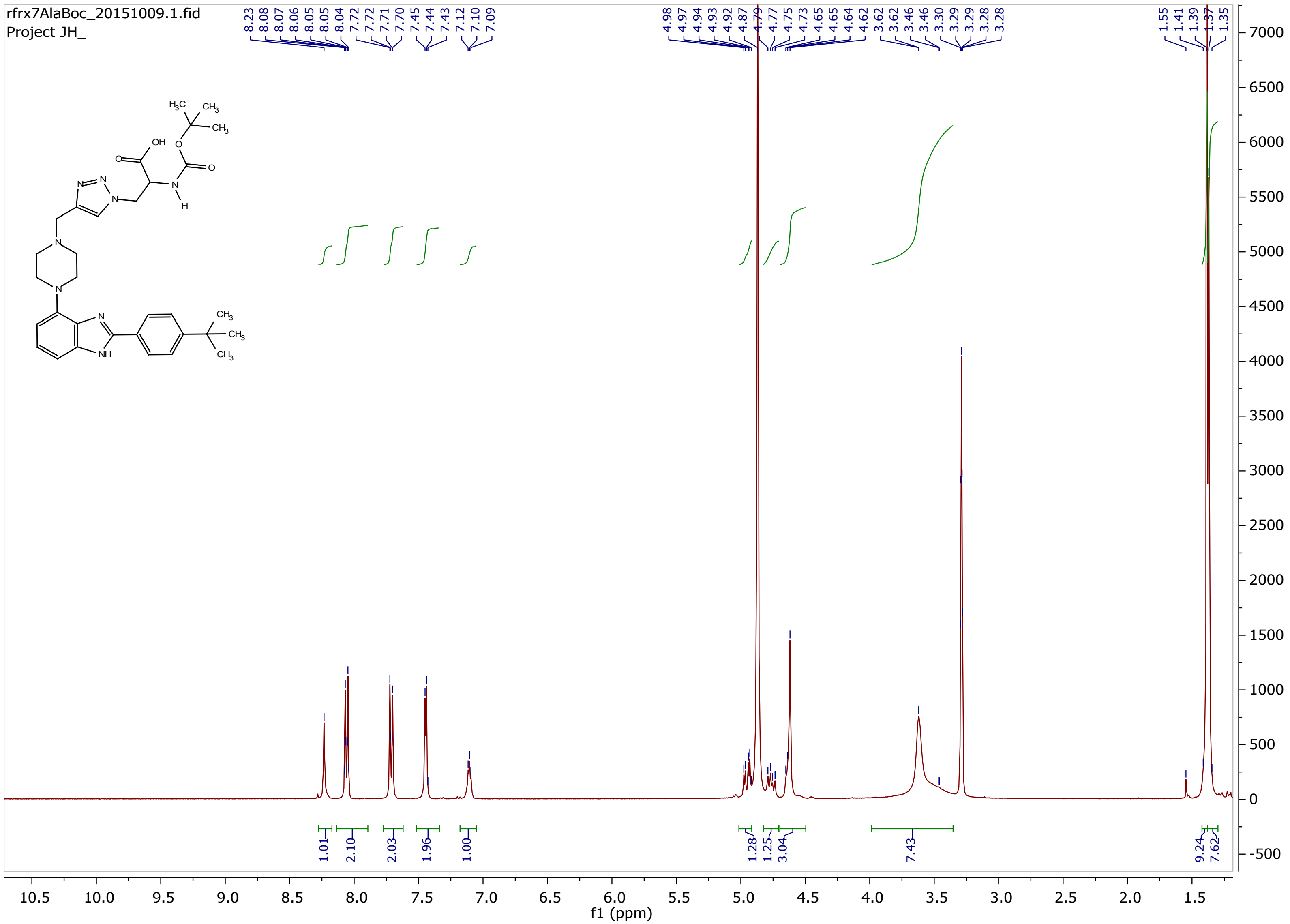

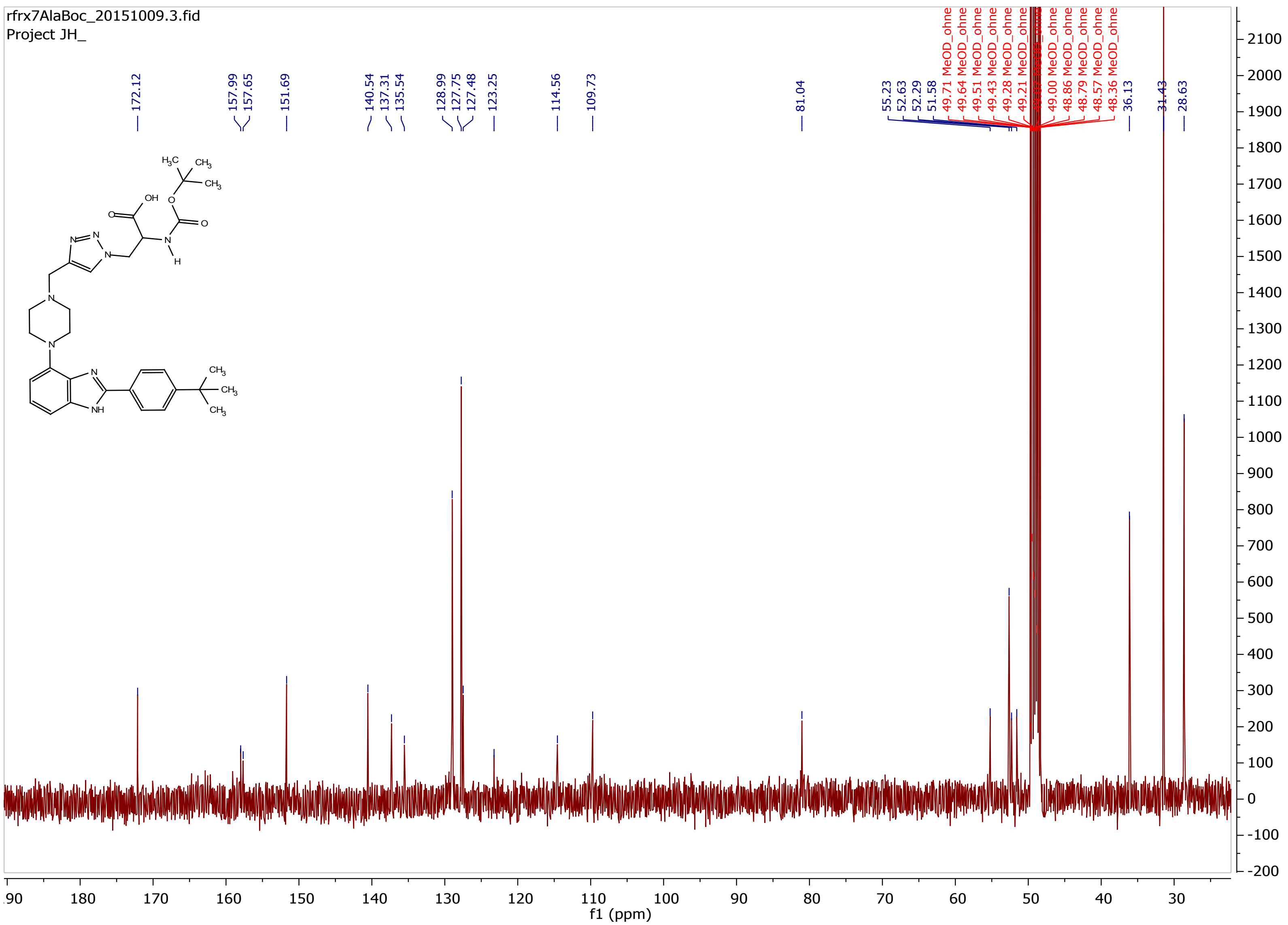

rfrx7AlaBoc_20151009.3.fid
Project JH_
172.12
157.99
157.65
151.69
140.54
137.31
135.54
128.99
127.75
127.48
123.25
114.56
109.73
81.04
55.23
52.63
52.29
51.58
49.71 MeOD_ohne
49.64 MeOD_ohne
49.51 MeOD_ohne
49.43 MeOD_ohne
49.28 MeOD_ohne
49.21 MeOD_ohne
49.00 MeOD_ohne
48.86 MeOD_ohne
48.79 MeOD_ohne
48.57 MeOD_ohne
48.36 MeOD_ohne
36.13
31.43
28.63
f1 (ppm)

2-(4-*tert*-Butylphenyl)-4-(4-((1-(2-fluoroethyl)-1H-1,2,3-triazol-4-yl)methyl)piperazin-1-yl)-1H-benzo[d]imidazole (8a)

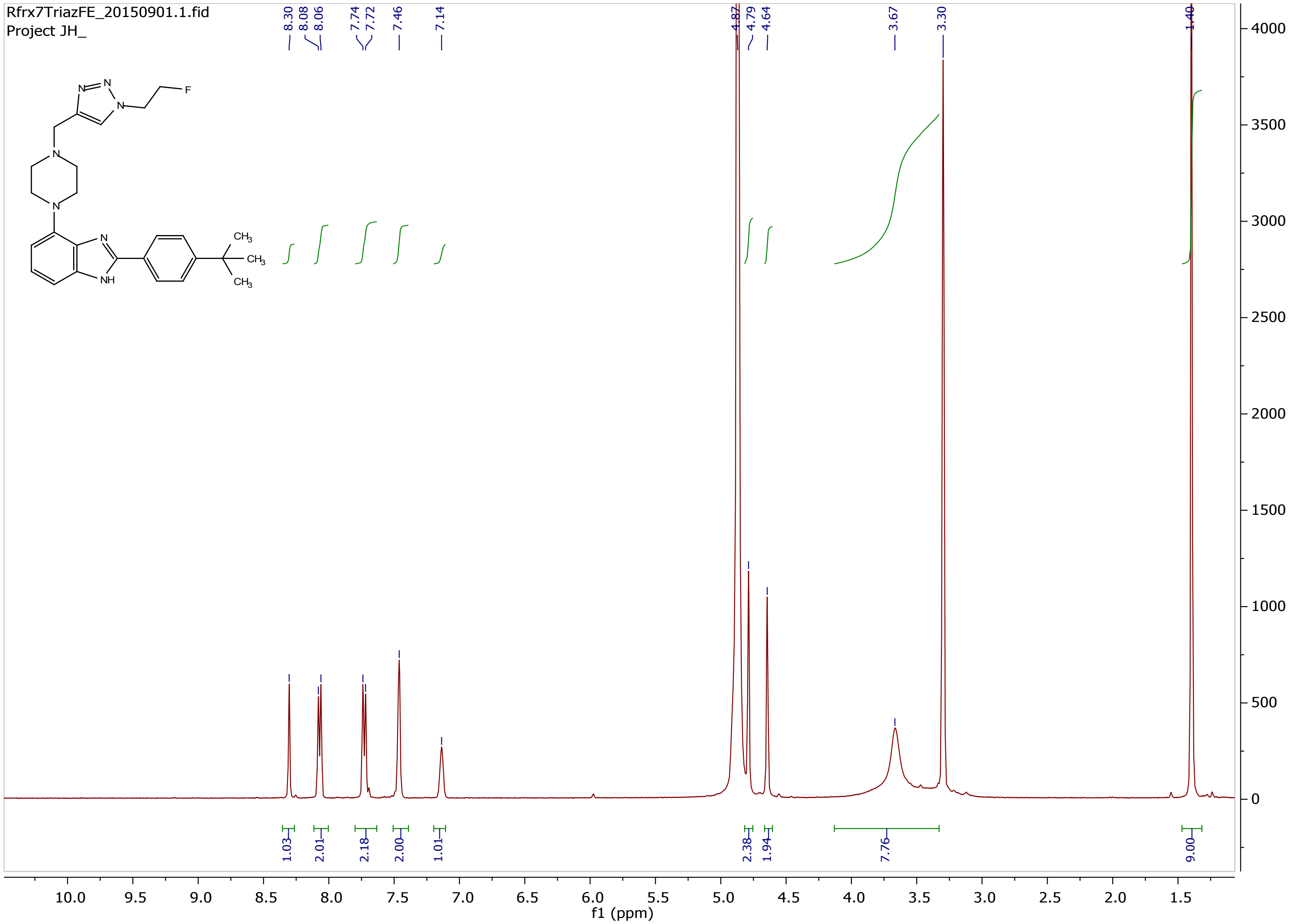

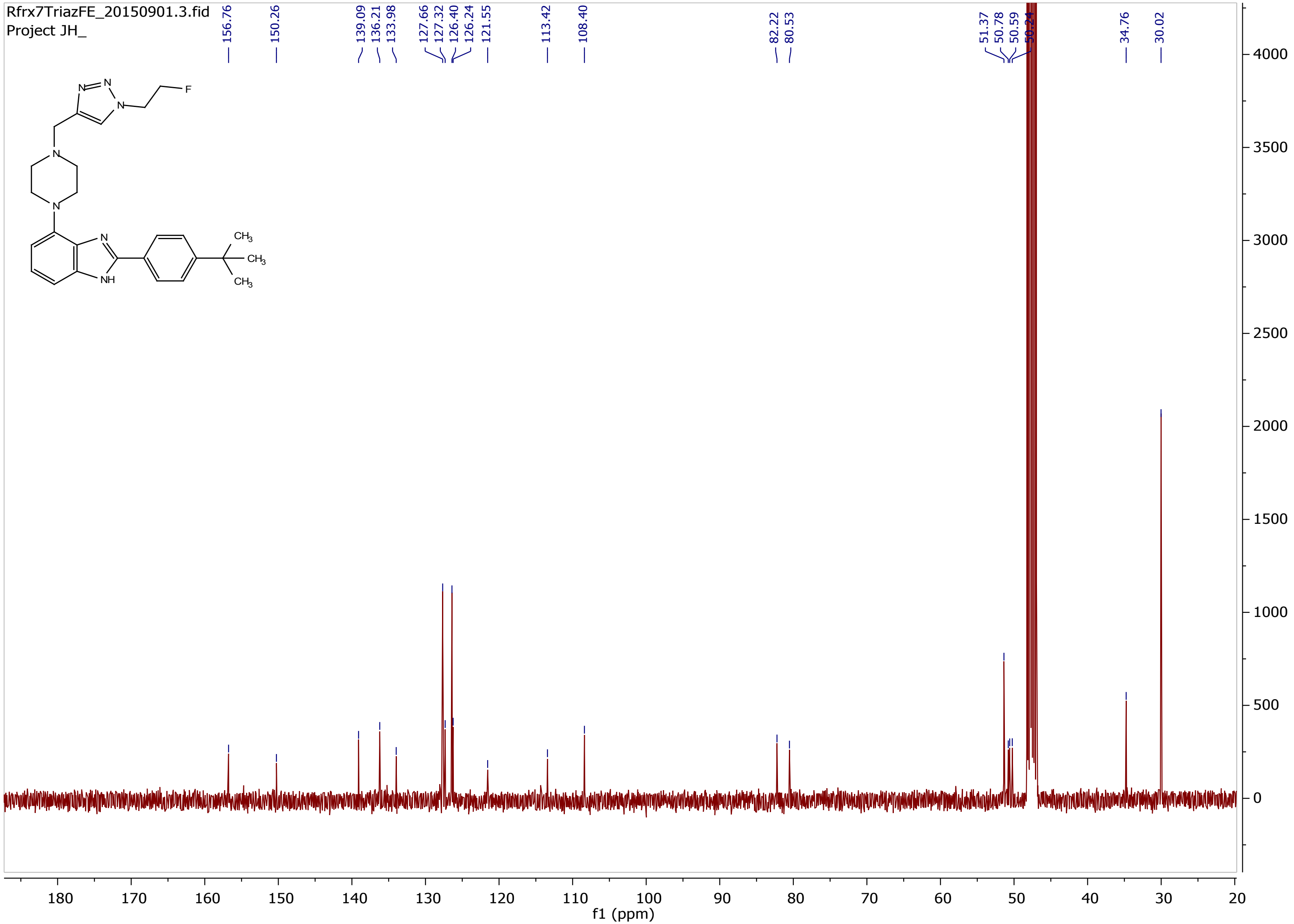

Rfrx7TriazFE_20150901.3.fid
Project JH_
156.76
150.26
139.09
136.21
133.98
127.66
127.32
126.40
126.24
121.55
113.42
108.40
82.22
80.53
51.37
50.78
50.59
50.24
34.76
30.02
4000
3500
3000
2500
2000
1500
1000
500
0
180 170 160 150 140 130 120 110 100 90 80 70 60 50 40 30 20
f1 (ppm)

1-((2R,4R,5S)-4-(4-((4-(2-(4-*tert*-Butylphenyl)-1H-benzo[d]imidazol-4-yl)piperazin-1-yl)methyl)-1H-1,2,3-triazol-1-yl)-5-(hydroxymethyl)tetrahydrofuran-2-yl)-5-methylpyrimidine-2,4(1H,3H)-dione (8g)

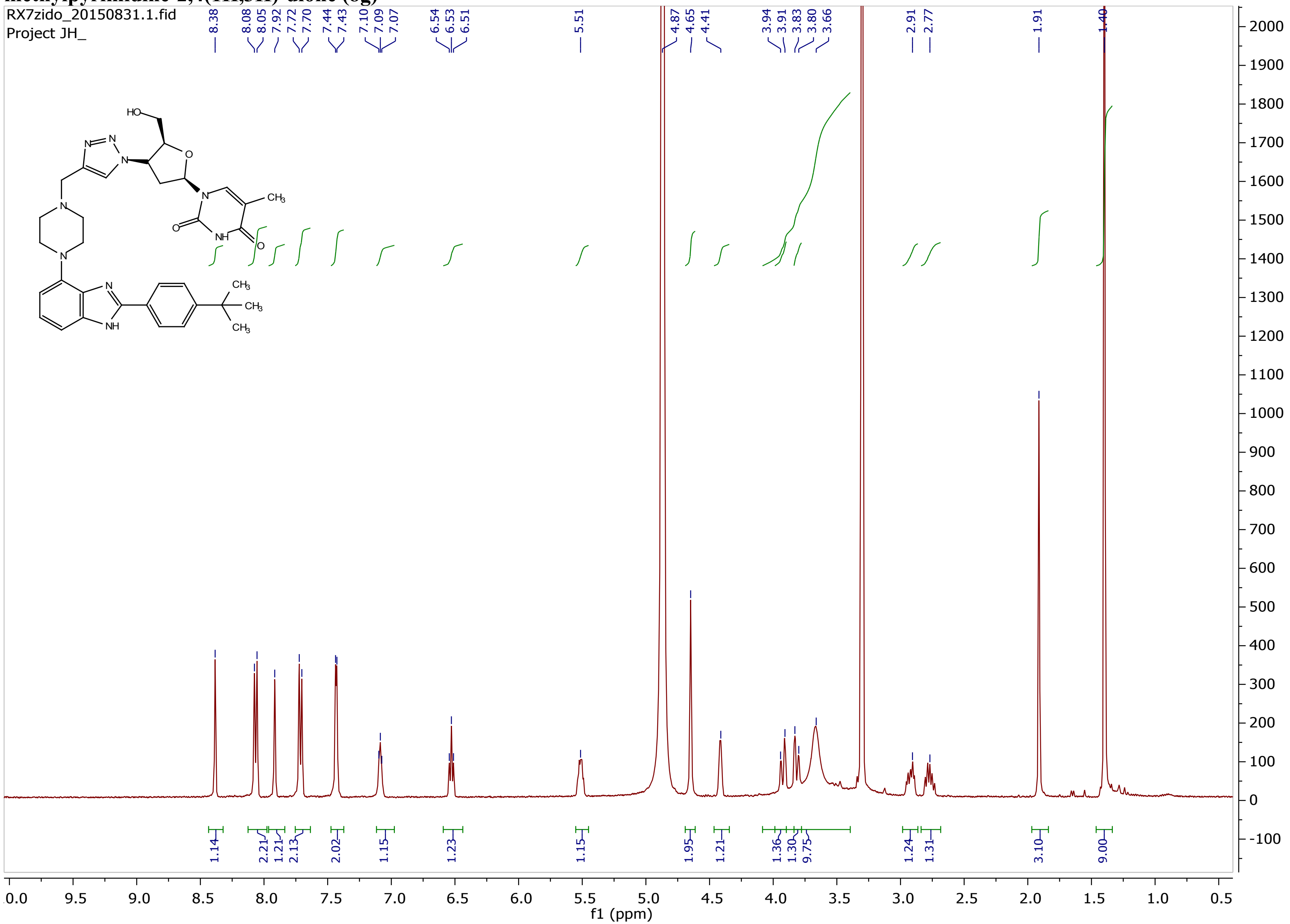

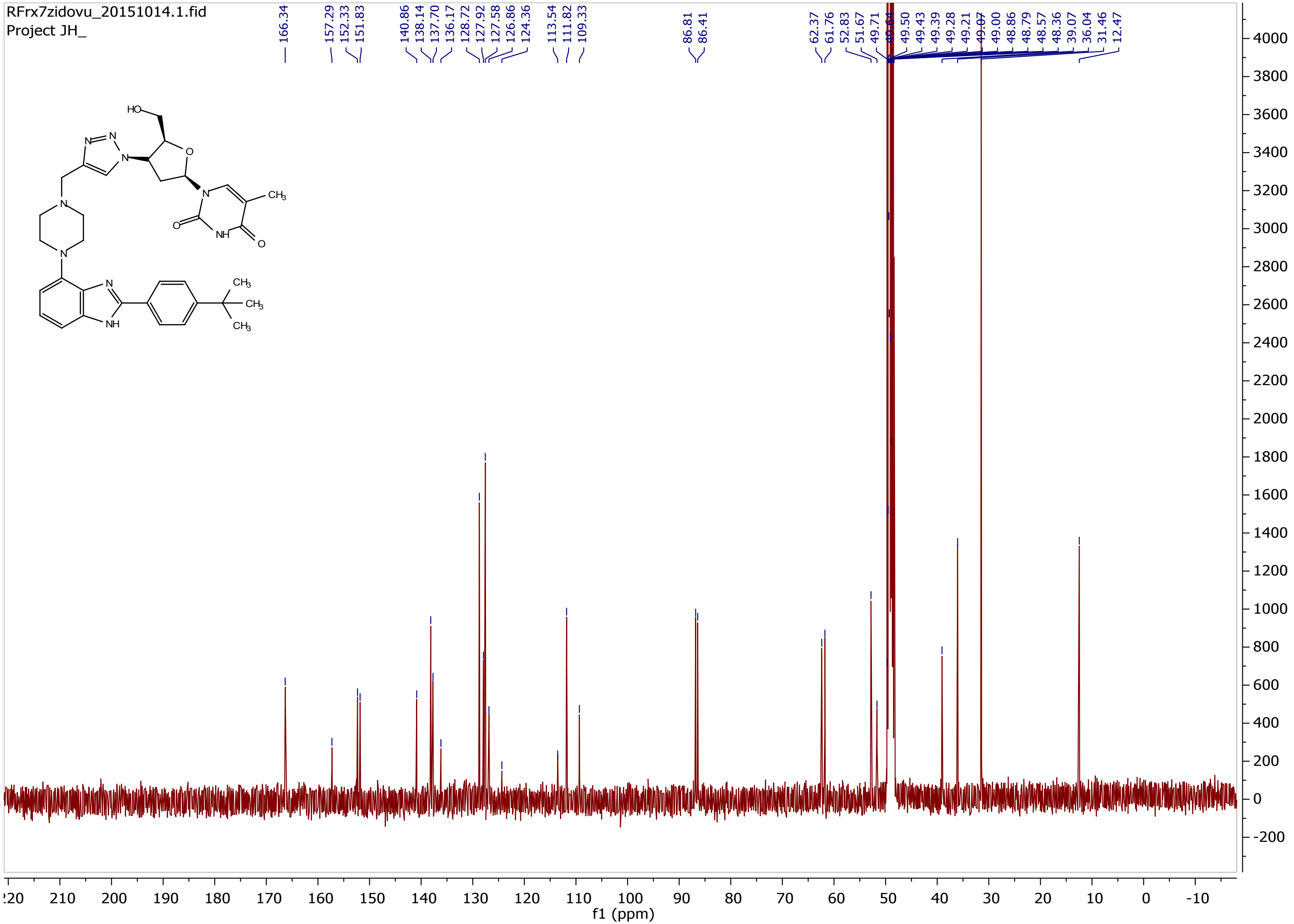

RFrx7zidovu_20151014.1.fid
Project JH_
166.34
157.29
152.33
151.83
140.86
138.14
137.70
136.17
128.72
127.92
127.58
126.86
124.36
113.54
111.82
109.33
86.81
86.41
62.37
61.76
52.83
51.67
49.71
49.64
49.50
49.43
49.39
49.28
49.21
49.07
49.00
48.86
48.79
48.57
48.36
39.07
36.04
31.46
12.47
HO
N
O
CH3
NH
f1 (ppm)

(6S)-2-(4-((4-(2-(4-*tert*-Butylphenyl)-1H-benzo[d]imidazol-4-yl)piperazin-1-yl)methyl)-1H-1,2,3-triazol-1-yl)-6-(hydroxymethyl)tetrahydro-2H-pyran-3,4,5-triol (8e)

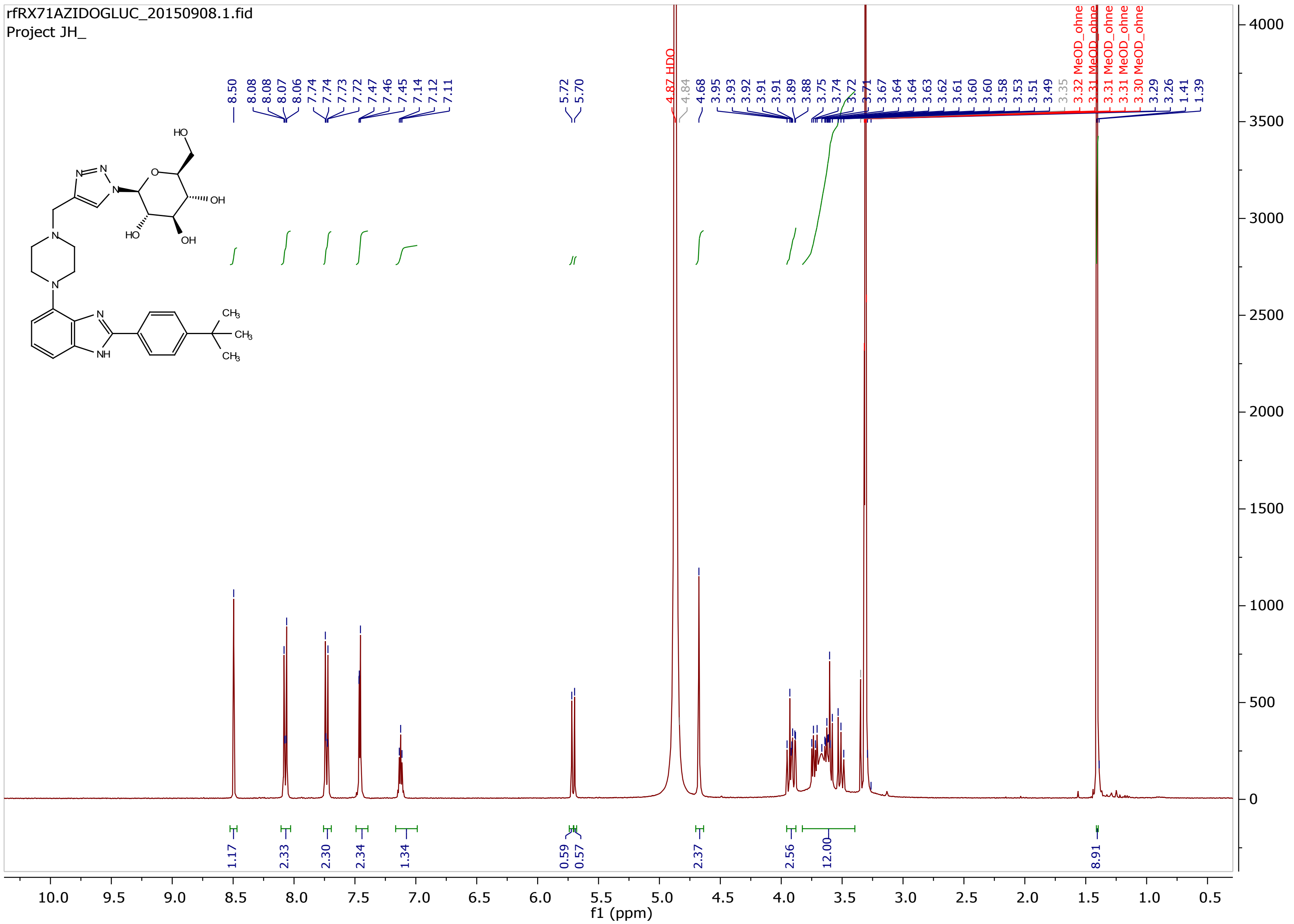

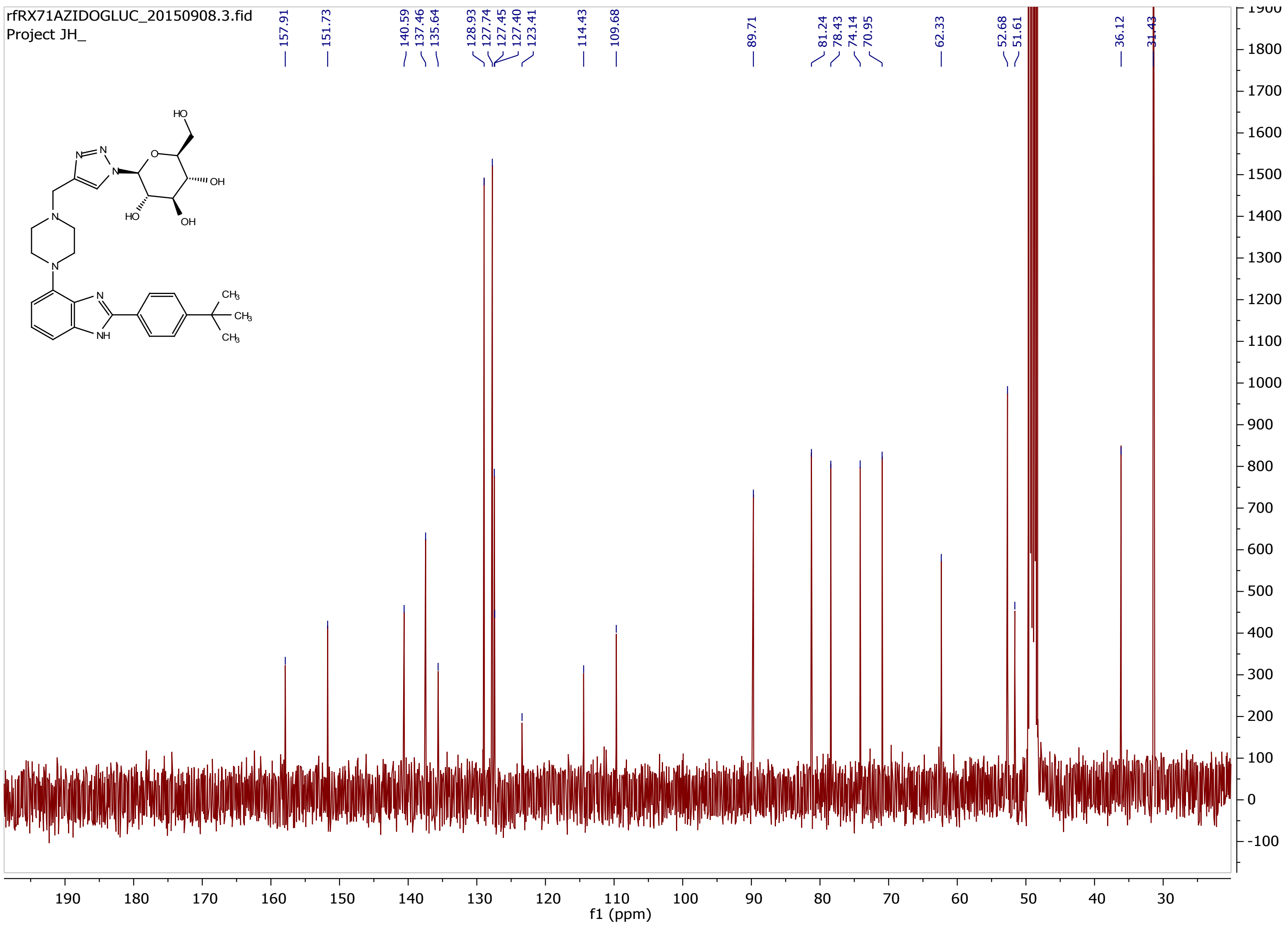

rfRX71AZIDOGLUC_20150908.3.fid
Project JH_
157.91
151.73
140.59
137.46
135.64
128.93
127.74
127.45
127.40
123.41
114.43
109.68
89.71
81.24
78.43
74.14
70.95
62.33
52.68
51.61
36.12
31.43
f1 (ppm)

N-((2R,3S,4S,5R,6S)-2-(4-((4-(2-(4-*tert*-butylphenyl)-1H-benzo[d]imidazol-4-yl)piperazin-1-yl)methyl)-1H-1,2,3-triazol-1-yl)-4,5-dihydroxy-6-(hydroxymethyl)tetrahydro-2H-pyran-3-yl)acetamide (8f)

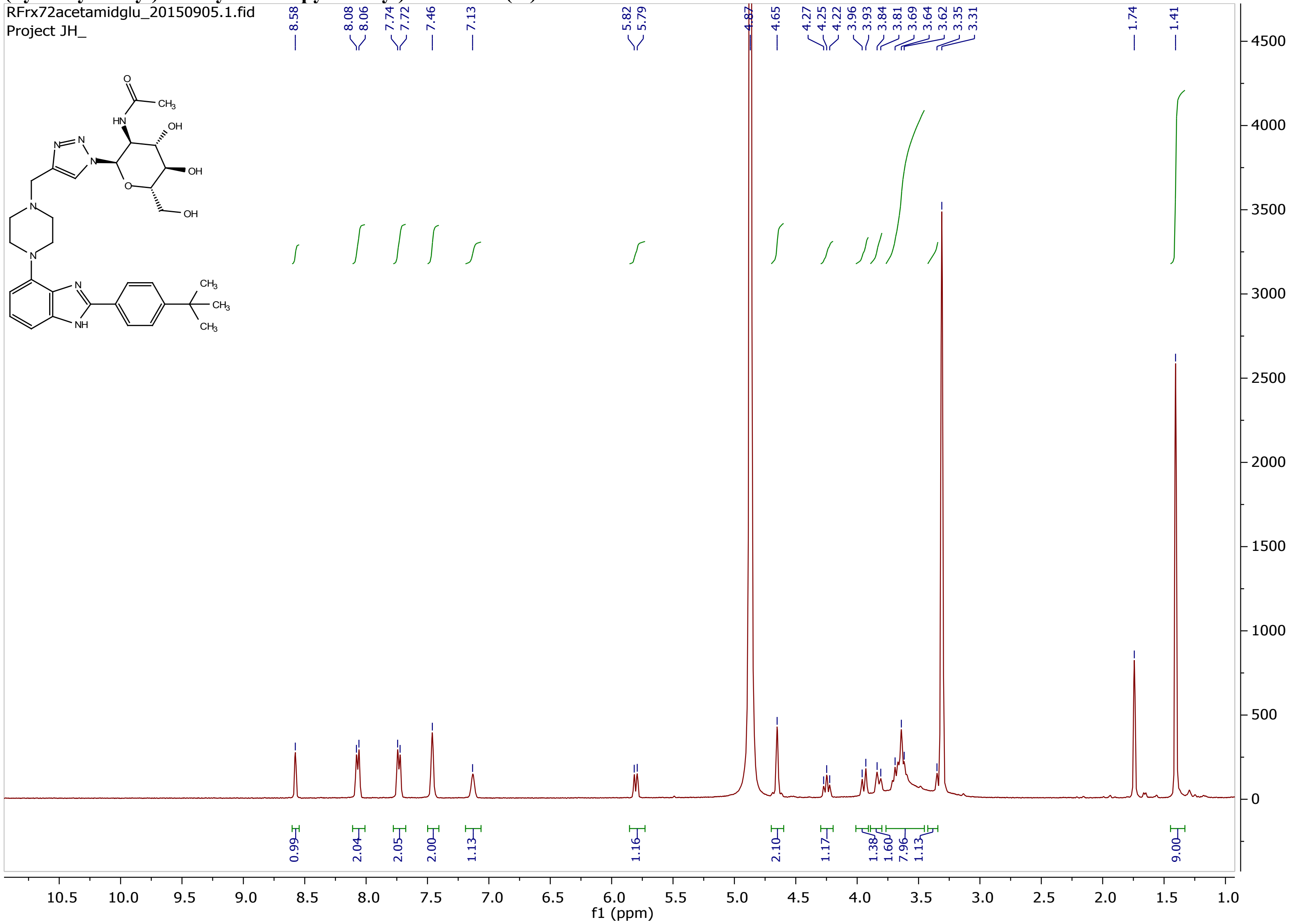

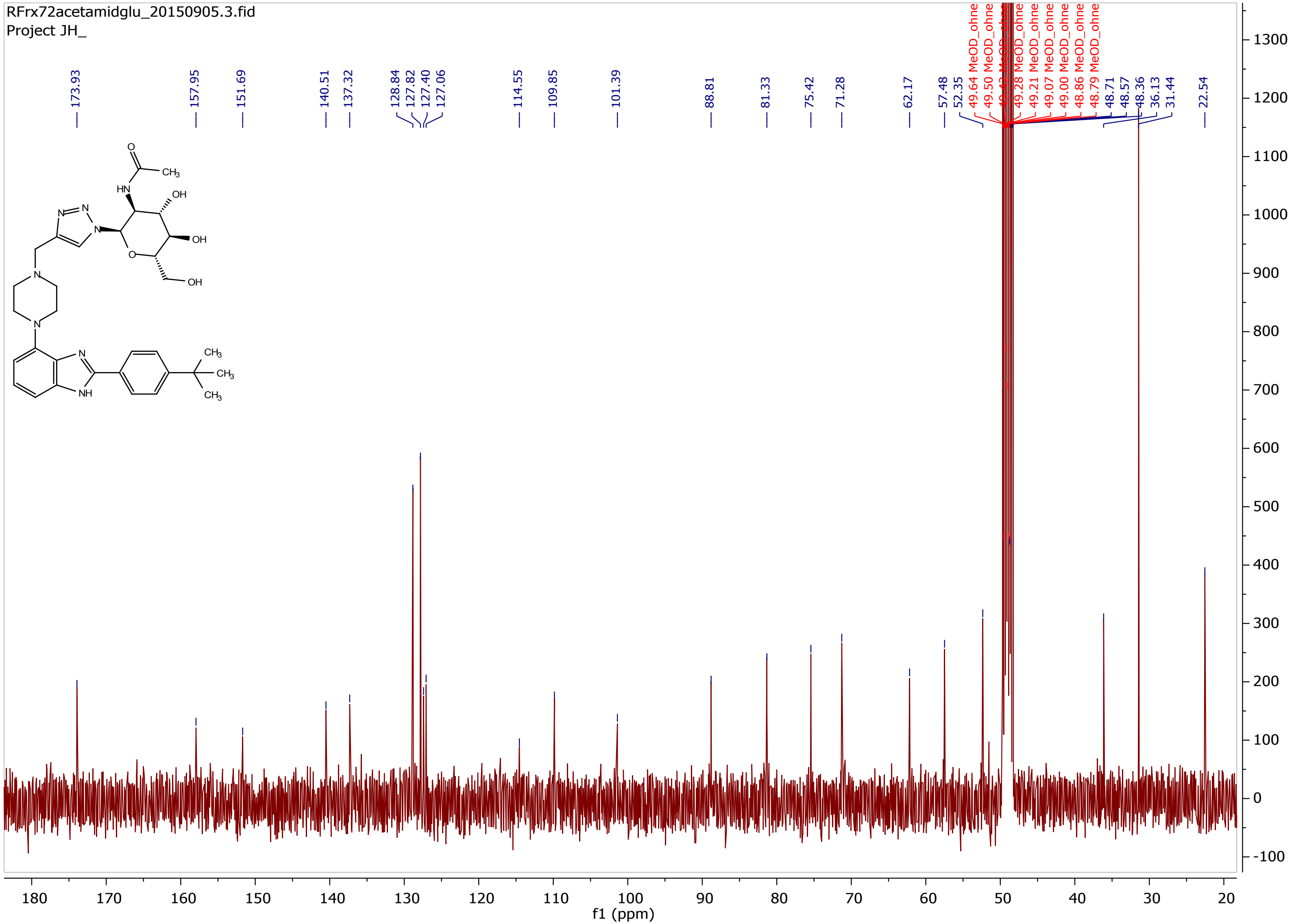

RFrx72acetamidglu_20150905.3.fid
Project JH_
173.93
157.95
151.69
140.51
137.32
128.84
127.82
127.40
127.06
114.55
109.85
101.39
88.81
81.33
75.42
71.28
62.17
57.48
52.35
49.64 MeOD_ohne
49.50 MeOD_ohne
49.28 MeOD_ohne
49.21 MeOD_ohne
49.07 MeOD_ohne
49.00 MeOD_ohne
48.86 MeOD_ohne
48.79 MeOD_ohne
48.71
48.57
48.36
36.13
31.44
22.54
f1 (ppm)

Regioisomeric mixture of 4-(4-((4-(2-(4-*tert*-butylphenyl)-1H-benzo[d]imidazol-4-yl)piperazin-1-yl)methyl)-1H-1,2,3-triazol-1-yl)-N-isopropyl-6-(methylthio)-1,3,5-triazin-2-amine (8j) and 4-(5-((4-(2-(4-*tert*-butylphenyl)-1H-benzo[d]imidazol-4-yl)piperazin-1-yl)methyl)-1H-1,2,3-triazol-1-yl)-N-isopropyl-6-(methylthio)-1,3,5-triazin-2-amine (8j)

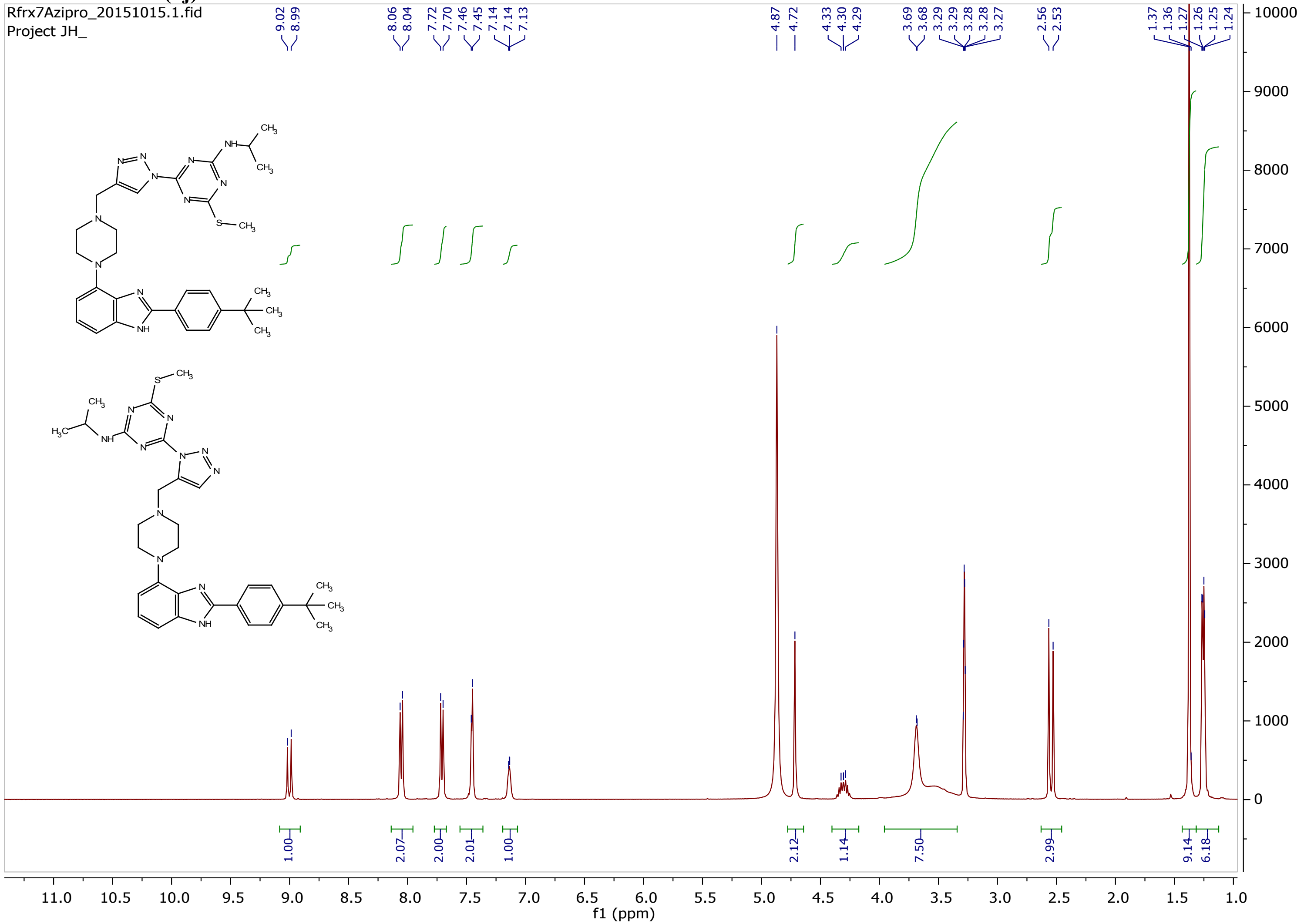

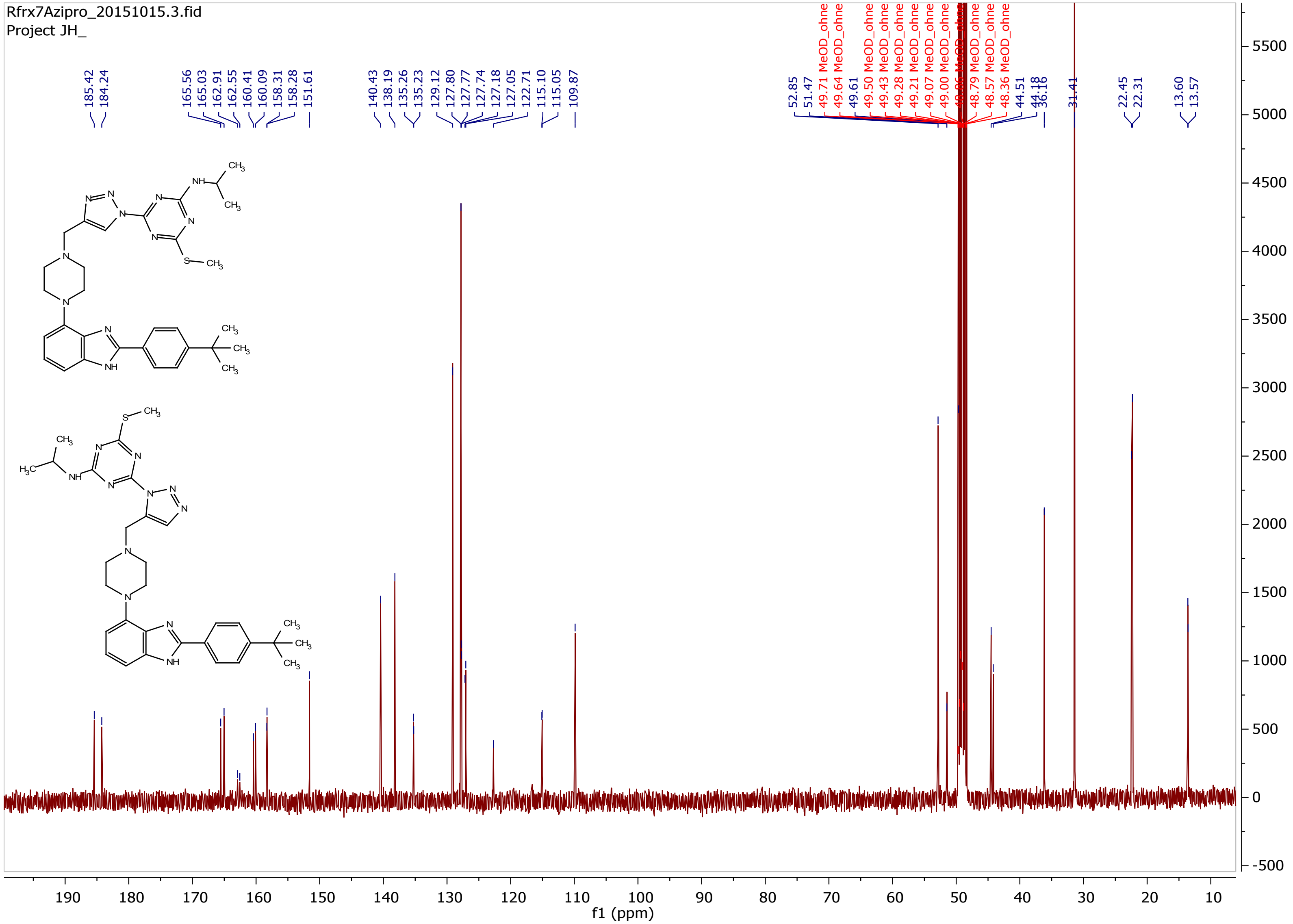

Rfrx7Azipro_20151015.3.fid
Project JH_
185.42
184.24
165.56
165.03
162.91
162.55
160.41
160.09
158.31
158.28
151.61
140.43
138.19
135.26
135.23
129.12
127.80
127.77
127.74
127.18
127.05
122.71
115.10
115.05
109.87
52.85
51.47
49.71 MeOD_ohne
49.64 MeOD_ohne
49.61
49.50 MeOD_ohne
49.43 MeOD_ohne
49.28 MeOD_ohne
49.21 MeOD_ohne
49.07 MeOD_ohne
49.00 MeOD_ohne
48.79 MeOD_ohne
48.57 MeOD_ohne
48.36 MeOD_ohne
44.51
36.16
31.41
22.45
22.31
13.60
13.57
f1 (ppm)

4-(4-((1-(1H-benzo[d]imidazol-2-yl)-1H-1,2,3-triazol-4-yl)methyl)piperazin-1-yl)-2-(4-*tert*-butylphenyl)-1H-benzo[d]imidazole (8h)

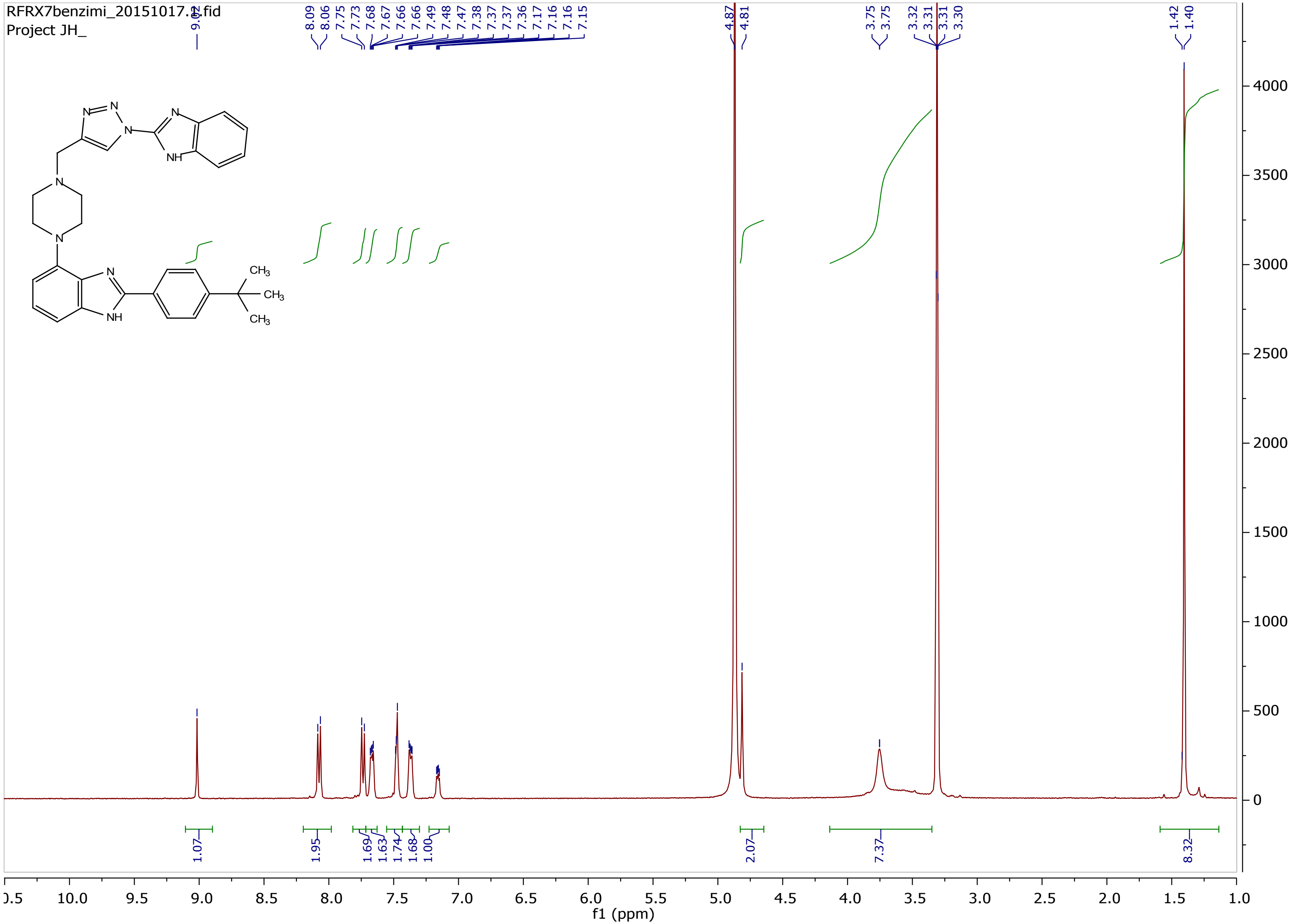

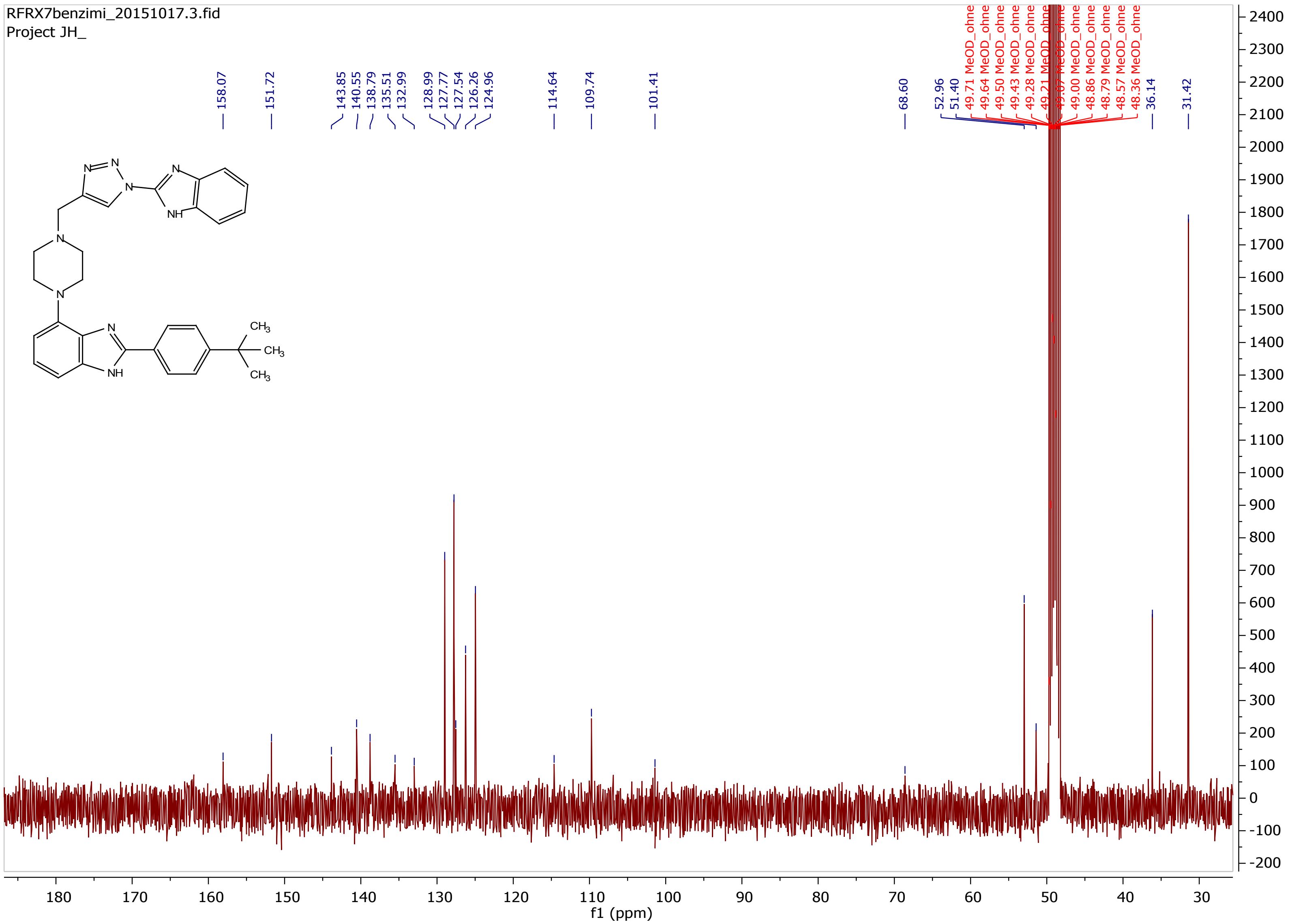

RFRX7benzimi_20151017.3.fid
Project JH_
158.07
151.72
143.85
140.55
138.79
135.51
132.99
128.99
127.77
127.54
126.26
124.96
114.64
109.74
101.41
68.60
52.96
51.40
49.71 MeOD_ohne
49.64 MeOD_ohne
49.50 MeOD_ohne
49.43 MeOD_ohne
49.28 MeOD_ohne
49.21 MeOD_ohne
49.00 MeOD_ohne
48.86 MeOD_ohne
48.79 MeOD_ohne
48.57 MeOD_ohne
48.36 MeOD_ohne
36.14
31.42
f1 (ppm)

2-(4-*tert*-Butylphenyl)-4-(4-((1-(4-fluorophenyl)-1H-1,2,3-triazol-4-yl)methyl)piperazin-1-yl)-1H-benzo[d]imidazole (8i)

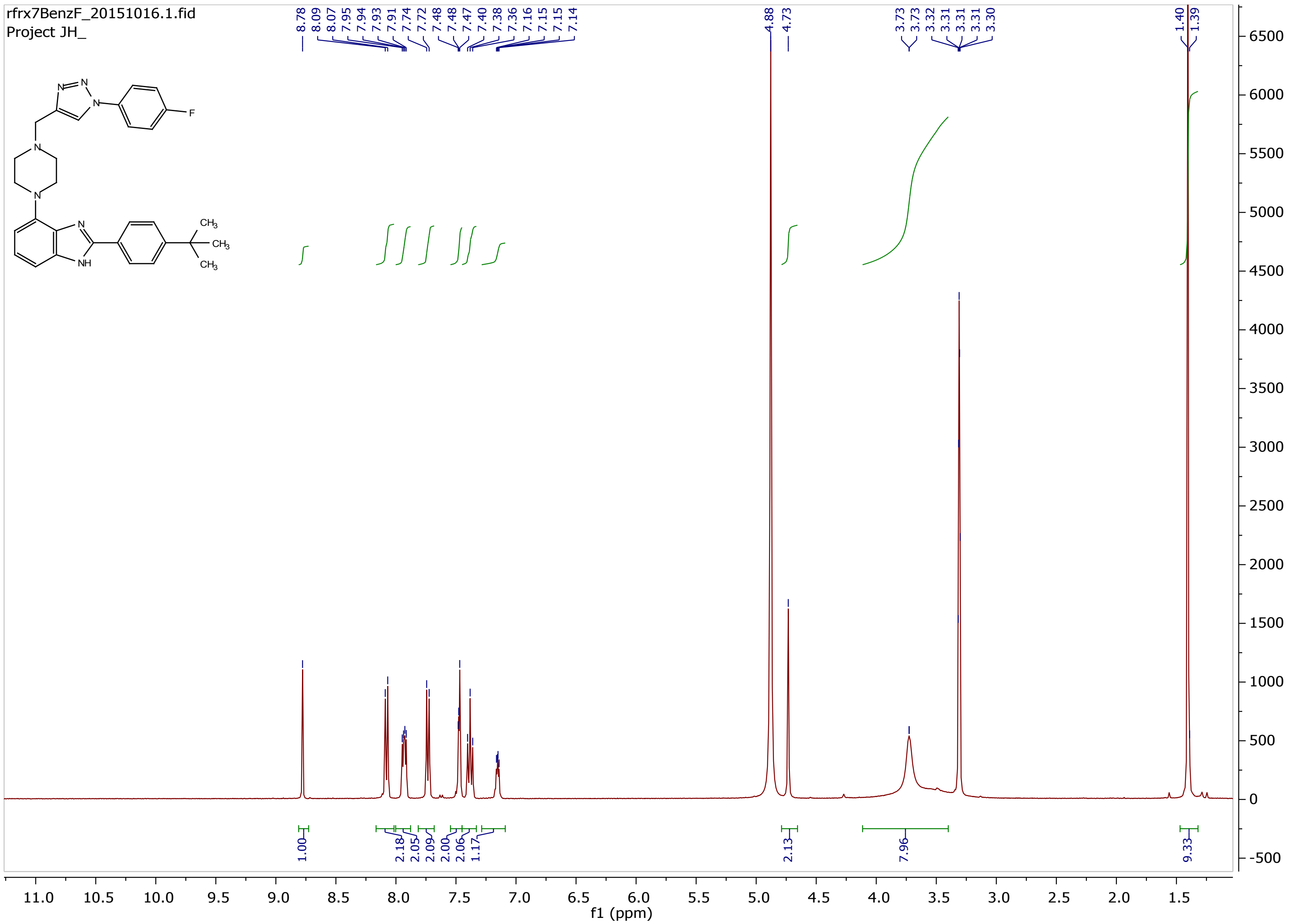

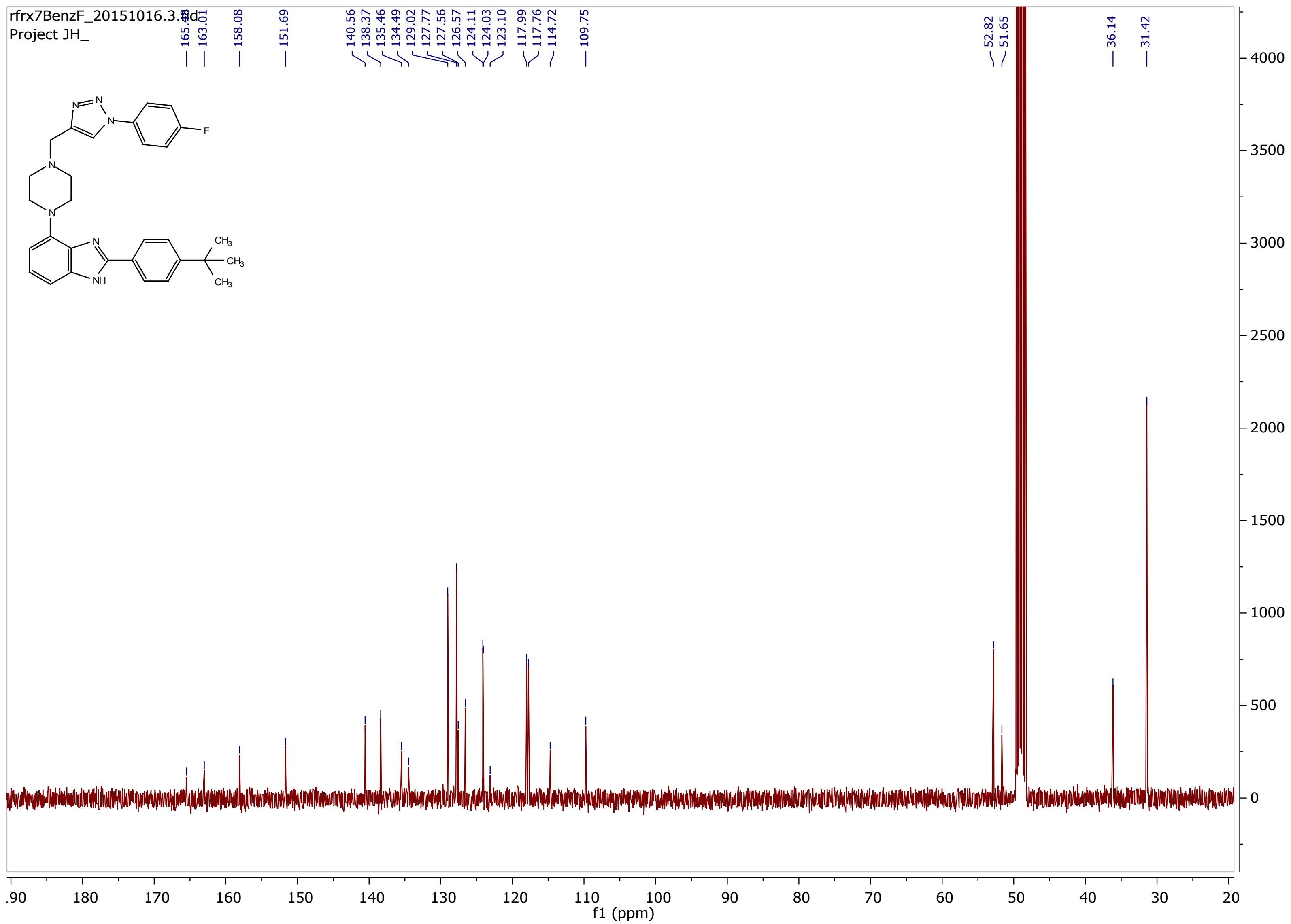
Project JH_
163.01
158.08
151.69
140.56
138.37
135.46
134.49
129.02
127.77
127.56
126.57
124.11
124.03
123.10
117.99
117.76
114.72
109.75
52.82
51.65
36.14
31.42
f1 (ppm)

Regioisomeric mixture of (2S,3S,4S)-5-(4-((4-(2-(4-*tert*-butylphenyl)-1H-benzo[d]imidazol-4-yl)piperazin-1-yl)methyl)-1H-1,2,3-triazol-1-yl)-4-(hydroxymethyl)tetrahydro-2H-pyran-2,3,6-triol (8d) and (2S,3S,4S)-5-(5-((4-(2-(4-*tert*-butylphenyl)-1H-benzo[d]imidazol-4-yl)piperazin-1-yl)methyl)-1H-1,2,3-triazol-1-yl)-4-(hydroxymethyl)tetrahydro-2H-pyran-2,3,6-triol (8d)

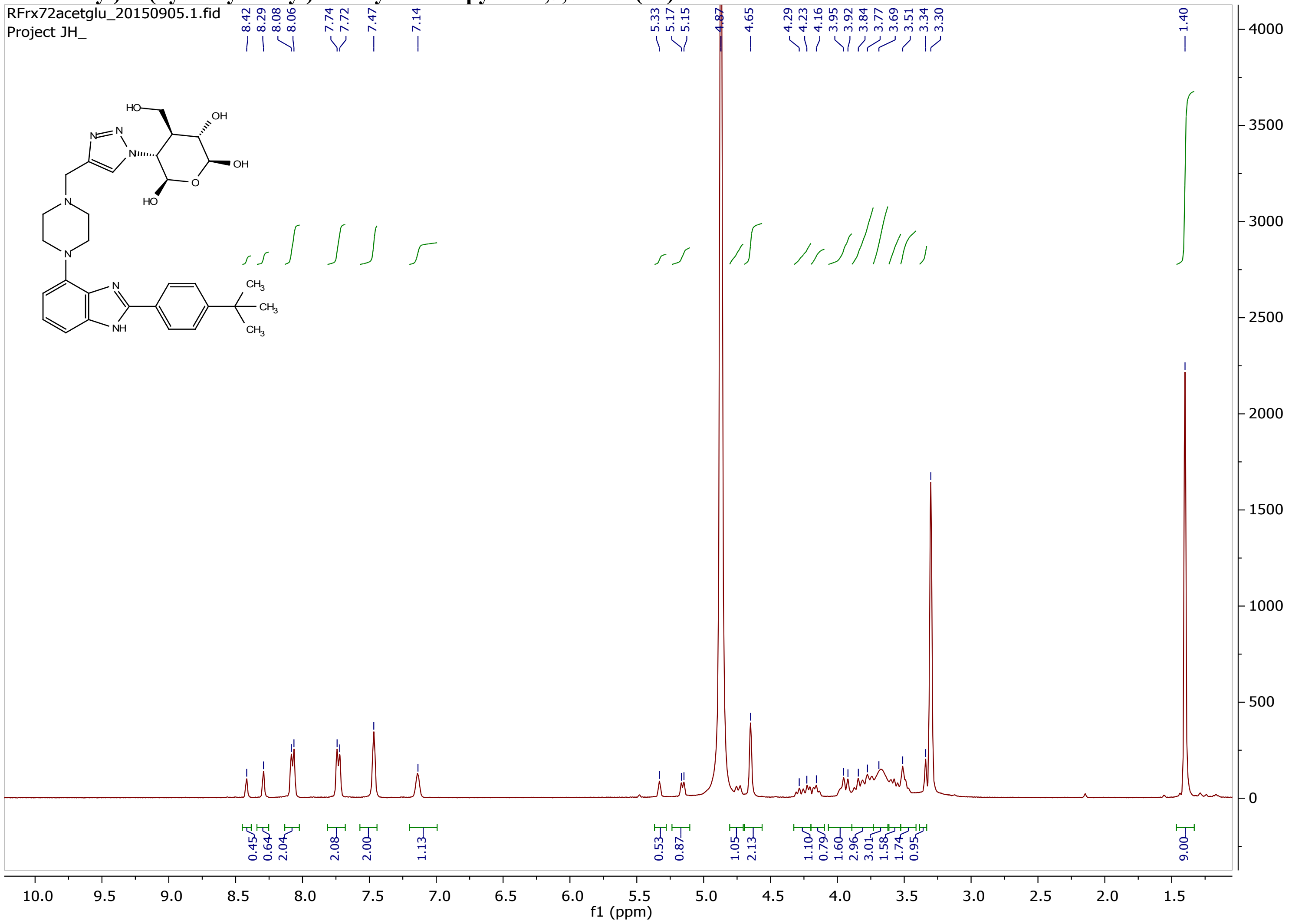

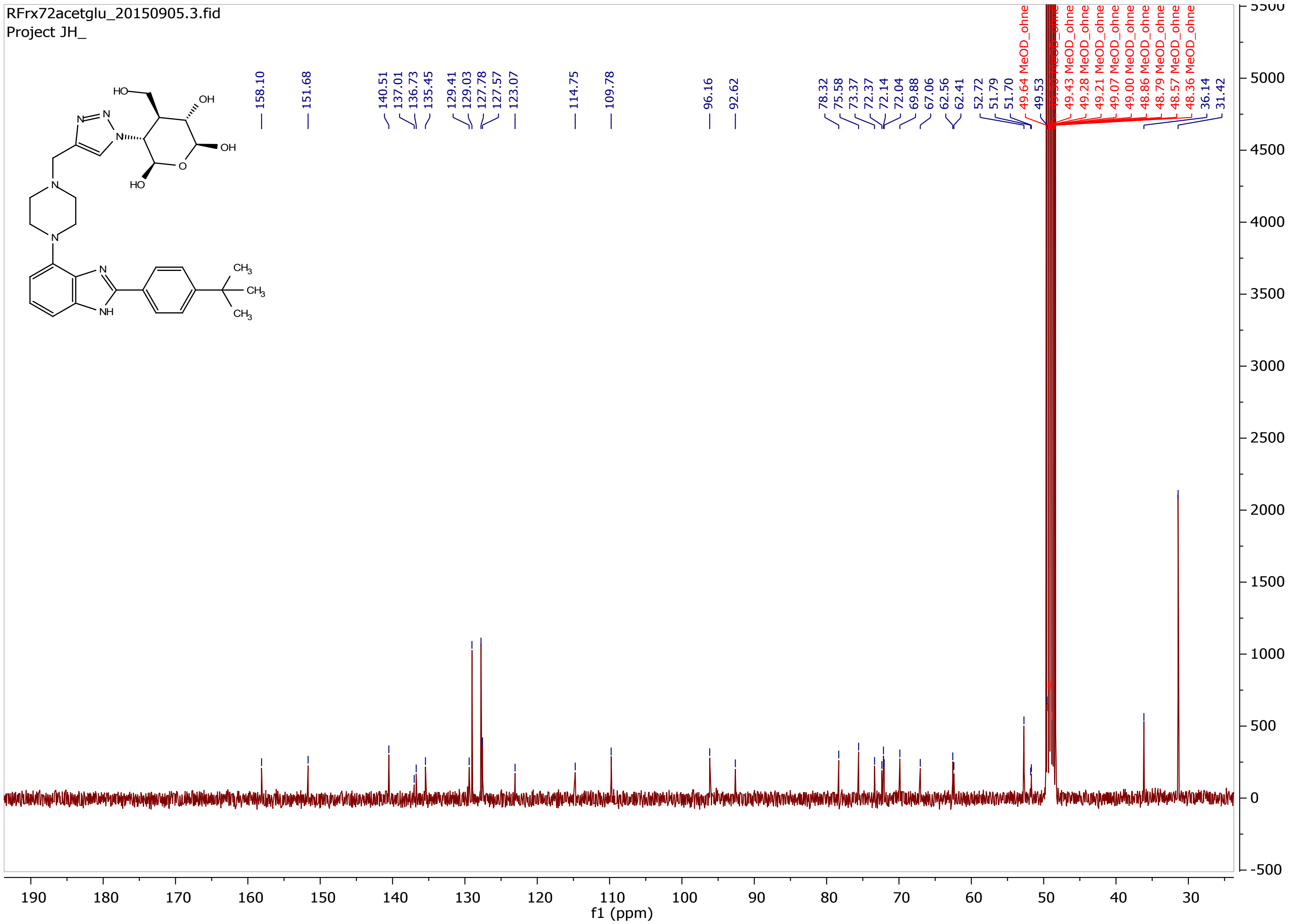
RFrx72acetglu_20150905.3.fid
Project JH_
158.10
151.68
140.51
137.01
136.73
135.45
129.41
129.03
127.78
127.57
123.07
114.75
109.78
96.16
92.62
78.32
75.58
73.37
72.37
72.14
72.04
69.88
67.06
62.56
62.41
52.72
51.79
51.70
49.64 MeOD_ohne
49.53
49.43 MeOD_ohne
49.28 MeOD_ohne
49.21 MeOD_ohne
49.07 MeOD_ohne
49.00 MeOD_ohne
48.86 MeOD_ohne
48.79 MeOD_ohne
48.57 MeOD_ohne
48.36 MeOD_ohne
36.14
31.42
HO
OH
N
N
OH
HO
O
N
N
CH3
CH3
CH3
NH
f1 (ppm)
